# Application of Nanofluids in Gas Turbine and Intercoolers—A Comprehensive Review

**DOI:** 10.3390/nano12030338

**Published:** 2022-01-21

**Authors:** Ali Almertejy, Muhammad M. Rashid, Naser Ali, Salah Almurtaji

**Affiliations:** 1 Department of Mechatronics Engineering, Faculty of Engineering, International Islamic University Malaysia, Jalan Gombak, Kuala Lumpur 53100, Malaysia; mahbub@iium.edu.my; 2 Kuwait Army, Kuwait Ministry of Defense, Safat 13128, Kuwait; S.r.almerteki33@kuwaitarmy.gov.kw; 3 Nanotechnology and Applications Program, Energy and Building Research Center, Kuwait Institute for Scientific Research, Safat 13109, Kuwait; nmali@kisr.edu.kw; 4 School of Aerospace, Transport and Manufacturing (SATM), Cranfield University, Cranfield MK43 0AL, UK

**Keywords:** gas turbine, intercooler, heat transfer, heat exchanger, thermal efficiency, nanofluid

## Abstract

Today, the optimal use of non-renewable energy sources, reducing pollution, and increasing the efficiency of power-generating cycles are of particular importance. There are several ways to increase the efficiency of gas turbines; one that has recently attracted attention is to use an intercooler. However, the efficiency of the heat exchanger used in intercoolers depends on the type of heat exchanger, the characteristics of the operating fluid and the thermal boundary layers, and the pump speed. Improving the thermophysical properties of the working fluid is a passive method of increasing heat transfer, which has attracted the attention of those researching engineering applications. The current review addresses the latest methods of improving gas turbine efficiency using nanofluids and includes experimental and numerical studies. First, the general principles governing turbines are described, then the commonly used types of heat exchangers are introduced. Finally, studies on the use of nanofluids in heat exchangers are reviewed. The technology of producing nanoparticles that can be used in heat exchangers is also discussed. This review article can provide the reader with comprehensive information on making nanofluids and using them in heat exchangers used as intercoolers.

## 1. Introduction

**Figure 1:**
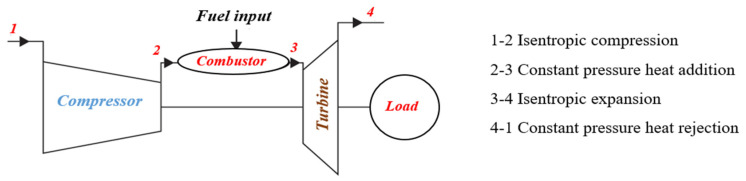
Schematic representation of a simple cycle gas turbine without intercooler [2].

**Figure 2:**
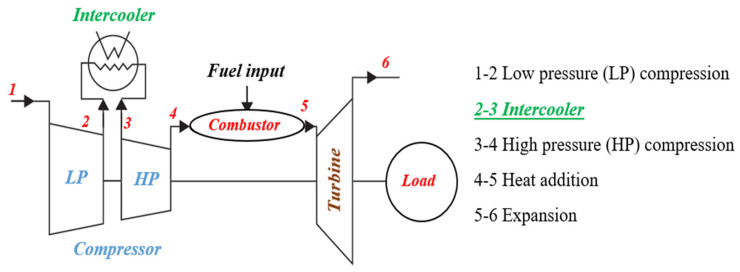
Schematic representation of a simple cycle gas turbine with intercooler [2].

Although power generation from nuclear power plants has been on the rise recently, in most countries electricity is generated from thermal or hybrid power plants with gas turbines the main components. The last 20 years represent a period of rapid advance for gas turbine technology driven by increased demand for this type of installation. Enhancing the efficiency of gas turbines can increase the efficiency of the power plant, reducing both air pollution and the consumption of non-renewable energy sources. There are several ways to increase the efficiency of a gas turbine. One is to increase the efficiency of the gas turbines by using an intercooler to reduce the gas temperature in the low-pressure compressor (LP), reducing the compressor power consumption and increasing the power generated by the turbine. Different heat exchangers are used in the intercoolers used in gas turbines. Schematics of the gas turbine cycle without and with an intercooler are shown in Figure 1 and Figure 2, respectively. The heat exchanger directly affects intercooler performance and gas turbine cycle efficiency. The type of heat exchanger and operational fluid flow regime have most effect on the efficiency of the heat exchanger [1].

There is a need for a comprehensive review paper concerning the application of nano-fluids in the heat exchanger of the intercoolers to improve gas turbine efficiency.

Increasing the heat transfer of heat exchangers is classified as one of two types: active or passive. The active method requires a secondary energy source to change the structure of the hydrodynamic and thermal boundary layers. The passive method does not need an energy source; heat transfer rates are increased by, for example, changing the surface roughness using vortex generators, and/or improving the thermophysical properties of the fluid including the use of nanofluids.

What makes this review paper different from previous reviews is its focus on literature reporting the use of nanofluids in heat exchangers that can be employed in intercoolers. It provides a comprehensive review of the research literature on increasing the efficiency of gas turbines using nanofluids. The paper also reviews methods of fabricating nanofluids and methods for measuring their thermophysical properties.

## 2. Structure of the Literature Review

Factors affecting gas turbine efficiency are introduced in Section 3, and the heat exchangers that can be used in the intercoolers are discussed in Section 4. These sections also present the fundamentals of the performance of gas turbine power plants and heat exchangers, especially surface plate-fin and shell-and-tube heat exchangers. In Section 5, the relevant literature on nanotechnology is reviewed. The effect of using different types of nanofluids on the performance of heat exchangers is presented with special attention given to the effect of design on system effectiveness, pressure drop, and fouling. In Section 6, the review covers the methods of measuring thermophysical properties such as thermal conductivity, acidity, stability, and friction of most types of nanofluids. Finally, after indicating the gaps in the literature, the key findings related to gas turbine technology, heat exchanger, and nanofluid are presented as Section 8, Conclusions.

## 3. Gas Turbine Power Plant Equipment Performance & Improvements

The performance and operational details of gas turbine power plants have been well-covered in the generally available literature, including traditional methods for improving the performance and effectiveness of heat exchangers such as the use of intercoolers and recuperators. Generally, academic research and industrial development in turbomachinery have been extensive and include gas turbine energy generation in industries such as aviation, power generation, and oil and gas. Despite decades of incremental improvements, the thermal efficiencies of simple cycles remain low, and the means to improve these generally involve the provision of heat exchangers, reheat, and intercooler compression.

**Figure 3:**
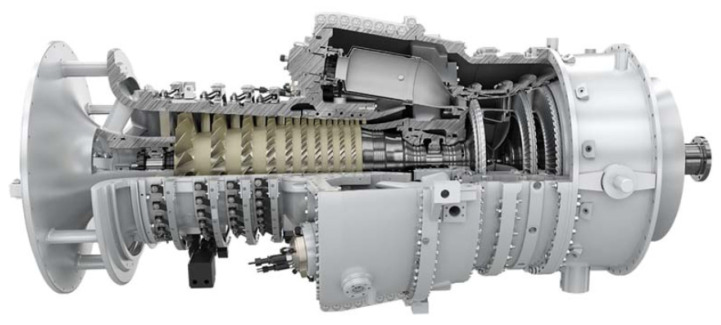
Siemens SGT-100 gas turbine.

Three factors differentiate the industrial gas turbine from the aircraft gas turbine. First, the operational life requirements of an industrial gas turbine are in the order of 50,000 h before the first shop visit or a major overhaul, almost ten times the duration of an aircraft gas turbine. Second, the importance of weight and size limitations on the aircraft propulsion system are not usually critical for industrial power plants. Third, the aircraft gas turbine can use the exhaust gas kinetic energy, whereas it might be wasted and minimized with other types. Figure 3 shows the typical Siemens SGT-100 industrial gas turbine. This unit has been used in such different applications for generating electricity and as a turbo gas engine in aircraft.

In general, gas turbines technology has three main application fields, which are:

First: open cycle gas turbines, which produce only power,

Second: cogeneration systems in which both heat and power are produced,

Third: a combination of both of the above.

The different applications can significantly affect the design, and therefore it is essential to identify the requirements during the design phase.

However, in the last decade, there has been significant investment in introducing aero-derivative gas turbines to industrial applications. These are popular for energy generation due to their reliability, flexibility, and higher efficiency. Advanced aerospace engine technologies have produced gas turbines with lighter weight, smaller size, faster response, and lower emissions than their heavy industrial counterparts. With up to 45% efficiency compared to 35% for heavier gas turbines, they are often good for smaller-scale (up to 100 MW) energy generation plants. The aero turbines are also popular due to their fuel flexibility as they operate on a range of natural gas and liquid fuel combinations [3].

A number of researchers have performed numerical studies on the factors affecting the performance of gas turbines, which will be reviewed and discussed here. Cetin [4] analyzed and optimized gas turbine performance numerically and analytically. He simulated turbine performance factors such as power output, work ratio, thermal efficiency, fuel consumption, rate of gas emission as a function of such operational parameters as compressor inlet temperature, turbine inlet temperature, maximum pressure and temperature, and heat exchanger effectiveness. The results showed that decreasing the air inlet temperature increased the net power of the simple cycle. Also, increasing the inlet turbine temperature improved the plant’s thermal efficiency.

The vital factor affecting the performance of the gas turbine is thermal efficiency. Although the thermal efficiency of open cycle gas turbine systems is very low, they are still the preferred option to supply peak electricity loads because these plants can be brought up to an operational capacity quickly, and their costs are considerably lower than those of other systems [5].

Shi and Che [6] designed and tested a combined cycle power plant operated by natural gas and installed with waste heat recovery. Parametric analyses were performed for the proposed combined cycle to evaluate the effects of several factors, including gas turbine inlet temperature, condenser pressure, fuel gas heating temperature, and the proposed cycle’s performance. When the condenser pressure was decreased for a given turbine inlet temperature, the net electrical efficiency of the proposed cycle increased. Heating the fuel resulted in higher gas turbine efficiency due to the reduced fuel flow. The effect of compressor design parameters such as inlet temperature, pressure ratio, gas specific heat on the gas turbine performance were investigated numerically by Xie et al. [7]. They developed a theoretical model for a closed, simple Brayton gas turbine cycle. The factors affecting the power output and thermal efficiency were investigated. The results showed that decreasing compressor work by reducing the specific volume of inlet air increased the net power of the cycle and increased thermal efficiency.

Kurt et al. [8] numerically investigated the factors affecting the performance of an open cycle gas turbine. Thermodynamic and exergy analyses of the cycle were carried out numerically to identify the best operation and optimization factors. Also, the effect of inlet air temperature into the compressor on the performance of the ideal gas turbine was studied, as was the effect of flow gases into the first stage of the gas turbine. The pressure ratio, thermal efficiency, and power output of the cycle were taken as the main performance parameters. The Brayton gas turbine cycle was analyzed and modeled numerically. Most factors affecting the performance of the cycle were checked directly. Table 1 shows the optimum pressure ratio value according to turbine inlet temperature for maximum net power output at ma = 1 kg/s, with compressor inlet temperature 288.15 K, turbine efficiency ηc = 0.85, and η = 0.88. Table 2 also shows the effect of compressor inlet temperature on gas turbine performance. According to the results obtained from the thermodynamic analysis of the open cycle gas turbine system, the compressor inlet temperature (CIT), turbine inlet temperature (TIT), and pressure ratio (PR) are the parameters that most significantly effect gas turbine performance such as net power output, thermal efficiency, and fuel consumption of the cycle.

**Figure 4:**
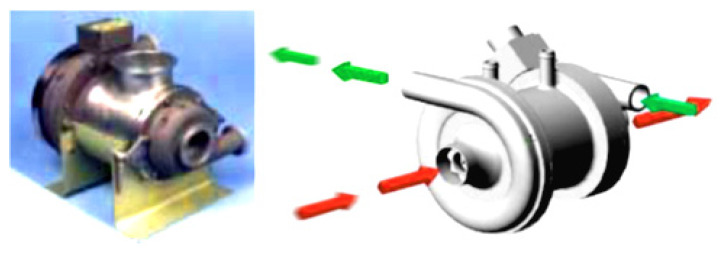
Honeywell turbo-compressor development from the first generation to the latest.

Generally, microturbines generate electrical power at a rate in the range of 5–200 kW, and mini turbines in the range of 200–500 kW. Typically, the thermal efficiency of microturbines is about 20% or less if no recuperator is included in the system. In this regard, Shah [9] designed and tested a compact heat exchanger of selected materials to improve the thermal efficiency of microturbines. He investigated the cost, performance, durability, and other related issues of a compact heat exchanger for use with microturbines. The inclusion of a recuperator exchanger with an effectiveness of 87% raised the efficiency of the gas turbine system to 30% approximately. However, the total cost of the plant was increased such that the cost of the recuperator was 25–30% of the total cost of the unit. Also, in the most regenerative Brayton cycle, which is based on using a recuperator for recovering heat from the turbine’s exhaust and using it to heat the compressed air to the combustion chamber, the performance of the cycle can be improved, increasing the efficiency, and reducing specific fuel consumption. Figure 4 shows the most efficient Honeywell Turbocompressor device used in micro-gas turbine power plant equipment.

Using these techniques with a ceramic recuperator heat exchanger of effectiveness 95% and turbine inlet temperature of about 1750 °C could raise the thermal efficiency of a microturbine system to about 50% [10].

Different factors affect compressor efficiency and energy consumption. The material of the blade or rotor has a significant effect, especially the inlet guide and outlet clearance (diffuser). High-density material used in the blades may increase the energy consumption due to an increase in inertial forces and the required torque. Honeywell has been developing turbomachinery devices, especially the Turbocompressor, since the 1960s for military and aviation applications [10], and now manufactures rotating bearings for turbomachinery equipment capable of providing 80,000 h of continuous operation and 50,000 h of starts/stops without any trouble. Such new technologies directly affect the net power of gas-turbines and work ratio and improve the cycle’s thermal efficiency.

**Figure 5:**
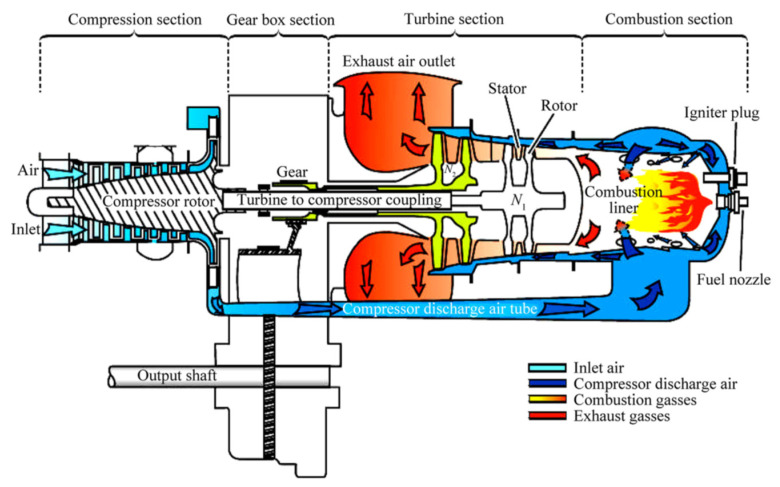
Turboshaft gas turbine engine instruction [11].

A technical investigation of a gas turbine was carried out experimentally and analytically by Barinyima et al. [11]. The study was carried out on the turboshaft engine cycles of a simple gas turbine helicopter engine before and after a recuperator device with high effectiveness was designed, built and inserted into the engine. Specific operational parameters such as inlet air temperature, fuel-specific heat, effectiveness, heat ratio, inlet turbine temperature, compressor efficiency, and turbine efficiency were modeled and analyzed. The percentage changes in important performance parameters such as thermal efficiency, specific fuel consumption, and power output of the modified engine were evaluated and compared to the simple cycle. It was found that to a large extent, the modified engine with unconventional components such as an engine cycle with a zero-stage in front of the low-pressure compressor, a recuperated engine cycle, and an intercooled recuperator exhibit better performances in terms of thermal efficiency and specific fuel consumption than traditional simple-cycle engines. The essential construction of a turboshaft gas engine is shown in Figure 5. It consists of a compression section, gearbox, turbine, and combustion section. As shown, the outlet gases are used to heat air before it enters the combustion section to increase the thermal efficiency of the cycle.

**Figure 6:**
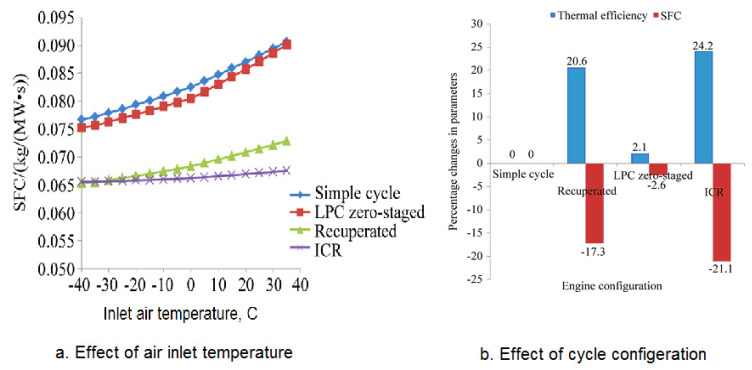
Percentage changes in performance parameters of modified cycles with respect to the simple cycle (ICR—intercooler cycle recuperated) [11].

The inlet air temperature has a big effect on the performance of gas turbine engines based on the cycle engine configuration. Using an intercooler leads to a decrease in the fuel consumption per Megawatt power output, as shown in Figure 6a. On the other hand, Figure 6b shows the gas turbine cycle’s thermal efficiency and specific fuel consumption (SFC) for such types as simple cycle and recuperated intercooler. It shows that the thermal performance of the Brayton cycle as based on the gas engine configuration. Using an intercooler heat exchanger between two compressors gave the highest cycle efficiency.

Zhang and Zhou [12] studied experimentally the spray characteristics of an air-blast atomizer unit of a type that can be used in most multi-point injectors to minimize the emission of flue gases from a gas turbine combustor. The method reduced fuel consumption and increased the net power, directly improving the performance of the gas turbine. In the paper, after their experiments, the authors provided a model for the characteristics of the air-blast atomizer unit. They found using the injector to blast air at high velocity into the fuel perpendicular to the fuel flow decreased the size of the fuel droplets, increased the air/fuel ratio, made the distribution of droplet diameter more uniform, and increased the turbulence in the flow, leading to an increase in the heat transfer in the combustion chamber.

Guven et al. [13] analyzed the exergy performance of a gas turbine with an intercooler and two reheaters. Numerous different fuels, methane, ethane, propane, butane, pentane, hexane, heptane, octane, nonane, decane, undecane, dodecane, tridecane, tetradecane, pentadecane, hexadecane, benzene, toluene, methanol, and ethanol were used in the gas turbine to investigate its energetic and energetic performance. The results showed that methane had the maximum exergy efficiency and the minimum exergy destruction, while methanol had the minimum exergy efficiency and maximum exergy destruction. The study was based on a thermodynamic model using the main performance equations of the gas turbine. It was found that the performance (thermal efficiency and mass-specific power) of a simple-cycle gas turbine increased monotonically with peak cycle temperatures. For a given peak cycle temperature, the performance also depended on cycle pressure ratio or peak cycle pressure. Maximum performance (mass-specific power and thermal efficiency near their maximum values) was achieved in a narrow range of optimum peak cycle pressures.

**Figure 7:**
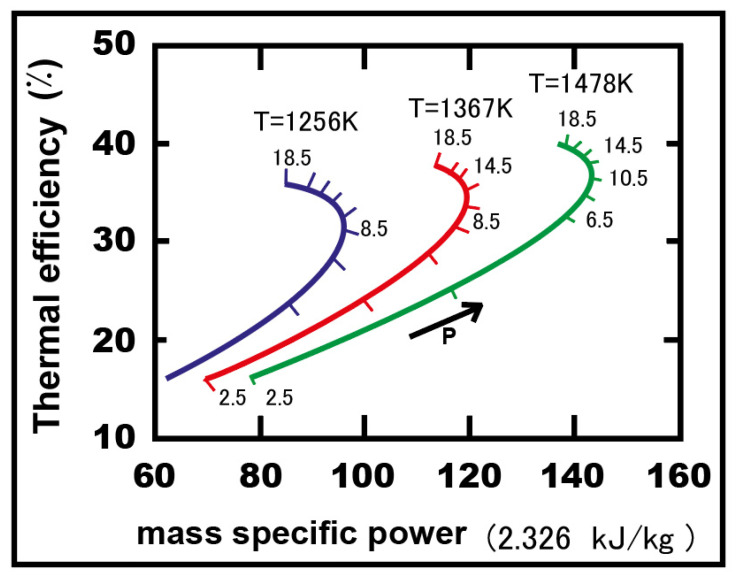
Examples of performance curves of Brayton-cycle gas turbines.

The study carried out by Wang and Pan [14], on improving gas turbine performance power output, thermal efficiency, air-fuel ratio, and heat transfer showed the effect of different important factors on the performance of a Brayton–supercharged cycle. Figure 7 shows the thermal efficiency as a function of mass-specific power for different pressure ratios and air compressor temperatures. The investigators investigated the performance of the Brayton cycle based on a reformulated intercooling-supercharging optimization procedure that was different from the cycle used in conventional intercooled devices. They used a new location for the intercooler device and a supercharged intercooler heat exchanger and, as a result, proposed using supercharged intercooling as a new technology to be applied in gas turbines.

The supercharged intercooled Brayton cycle increased the air peak pressure before it entered the combustion chamber. The peak pressure after the second compressor was increased, which considerably increased both the maximum pressure and the final highest temperature in the cycle. As a result, the thermal efficiency was directly increased due to the increase in the turbine’s power. Generally, the high-pressure intercooled cycle gave a better performance than the simple Brayton cycle.

The intercooled supercharged-cycle gas turbine achieved a new level of performance at these higher pressures: providing a 20% to 30% improvement over the simple cycle in terms of thermal efficiency and mass-specific power.

**Figure 8:**
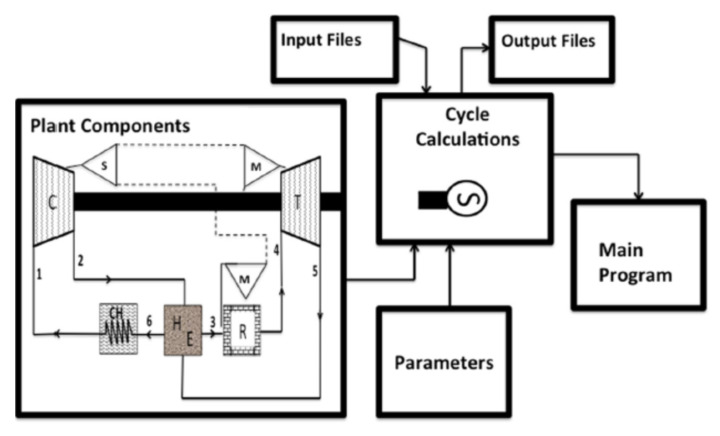
Typical Cycle Intercooler- Recuperated model [15].

Gad-Briggs et al. [15] studied the effect of cycle inlet temperature on the precooler and plant thermal efficiency based on simple and intercooled Brayton cycles. They applied their study to a nuclear power plant with gas and steam turbines where the inlet coolant temperature is important because variation in the coolant temperature results in a consequent variation in the precooler hot gas temperatures, which are cooled before re-entry. The study’s main aim was to analyze the effects of coolant inlet temperature on the heat sink and cycle efficiency. The cycles considered were Simple Cycle Recuperated (SCR), Intercooler Cycle Recuperated (ICR), and Intercooled Cycle without Recuperation (IC). The cycle was modeled, and performance was investigated analytically. The test rig used is shown in Figure 8 and was used to investigate the effects of changes in the inlet compressor temperature. In the case of using the intercooler and recuperator, the thermal efficiency is higher than for the cases of IC alone and SCR. Also, it was found that as inlet compressor temperature increased, the cycle efficiency in all cases decreased.

The result indicated that the IC had the highest hot gas temperature at the inlet to the pre-cooler for compressor inlet temperatures ≤ 50 °C. At a compressor inlet temperature > 50 °C, the SCR had the highest hot gas temperature at the pre-cooler inlet. This indicated that pre-cooler design is critical to both cycles to achieve modest heat sink exit temperatures. Tolerable heat sink temperatures will have a favorable effect on the coolant (helium) temperature at the compressor inlet, enabling both cycles’ design point thermal efficiencies to be maintained. The lowest hot gas temperature was observed with the ICR. The SCR would be suitable in cooler and mild climates, but not in a hot climate, as it would be challenging to justify its potential size due to the presence of a recuperator in addition to a large co-current pre-cooler as the counter-current pre-cooler would yield unfavorable heat sink return temperatures for cycle inlet temperatures above 30 °C.

Guo et al. [16] experimentally investigated the intercooled Brayton gas turbine cycle’s irreversibilities. They studied the effect of the number of heat transfer units distribution, compressor pressure ratio, specific heat ratios, etc., on the performance of an irreversible regenerative closed intercooled Brayton cycle in terms of thermal efficiency, thermodynamic efficiency, dimensionless power, entropy generation number, and Ecological Coefficient Of Performance (ECOP). There are optimal specific heat ratios, cycle pressure ratios, and intercooling pressure ratios for maximum thermal efficiency, maximum thermodynamic efficiency, and maximum ECOP. In general, the optimal specific heat ratio increases as the cycle pressure ratio decreases, and increases slightly as the intercooling pressure ratio increases. The optimal cycle pressure ratio increases, and the optimal intercooling pressure ratio decreases as the specific heat ratio decreases. The effect of the isentropic compressor and turbine efficiency was considered when analyzing power plant thermal efficiency and the ECOP of the intercooled cycle. Also, the effect of the specific heat of gases on the cycle performance was investigated. Decreasing the specific heat of gases increased the intercooler’s pressure ratio, which affected the power output and performance.

Most developments in efficiency are based on increasing compressor pressure ratio, advanced combustion techniques, improved material technology, new coatings, and new cooling schemes. The increase in gas turbine efficiency depends on the pressure ratio and peak temperature ratio [17]. Yang et al. [17] analyzed the performance of a regenerative-intercooled Brayton gas turbine cycle numerically and analytically. The study developed a thermodynamic model of an endoreversible intercooled regenerative Brayton heat and power cogeneration plant coupled to constant-temperature heat reservoirs using finite-time thermodynamics. The heat resistance losses in the hot, cold, and consumer-side heat exchangers were considered as were the performance of the heat transfer coefficients through the intercooler and regenerator. The finite-time exergoeconomic performance of the cogeneration plant was investigated numerically. The analytical study proposed dimensionless formulas for exergetic efficiency.

**Figure 9:**
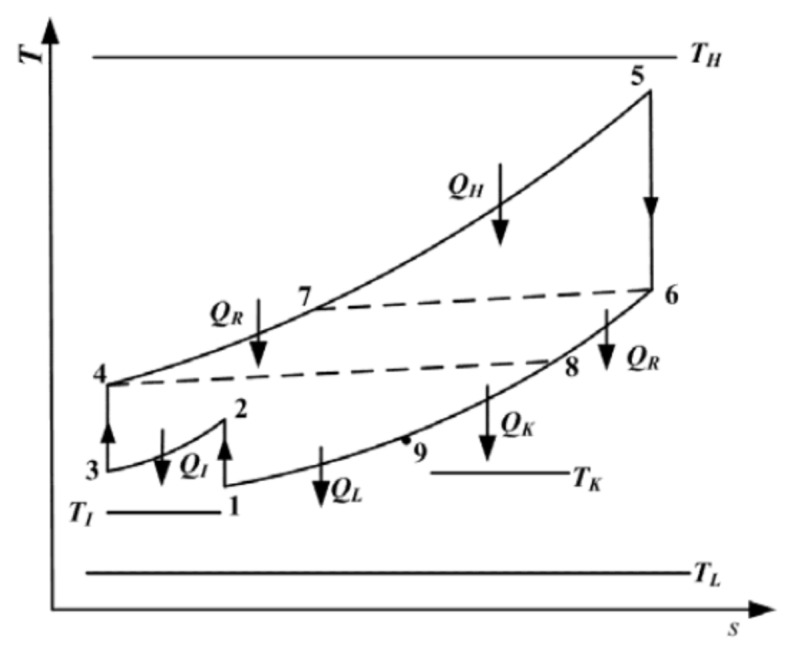
*T-s* diagram for the cycle model [17].

The numerical data showed that there existed an optimal value of intercooling pressure ratio that led to an optimal value of dimensionless profit rate for a fixed total pressure ratio and thermal efficiency of the cycle. The cycle was modeled as shown in Figure 9, where the *T-s* diagram of the heat and power cogeneration gas plant was composed of an endoreversible intercooled regenerative Brayton closed-cycle coupled to constant-temperature heat reservoirs. The lines marked 1–2 and 3–4 are isentropic low and high compressor processes, while process 5–6 is the adiabatic isentropic turbine expansion process. Process 2–3 is the intercooler process, which occurs under constant pressure (isobaric), and process 4–7 is the regenerative heat exchanger (recuperator), also at constant pressure, where heat is extracted from the exhaust gases. Process 7–5 is an isobaric process on the hot-side heat exchanger, whereby heat is absorbed. On the other hand, process 6–8 is an isobaric, evolved heat process in the regenerator, and process 8–9 is an isobaric developed heat process in the consumer-side heat exchanger. Process 9–1 is also an isobaric evolved heat process in the cold-side heat exchanger.

**Figure 10:**
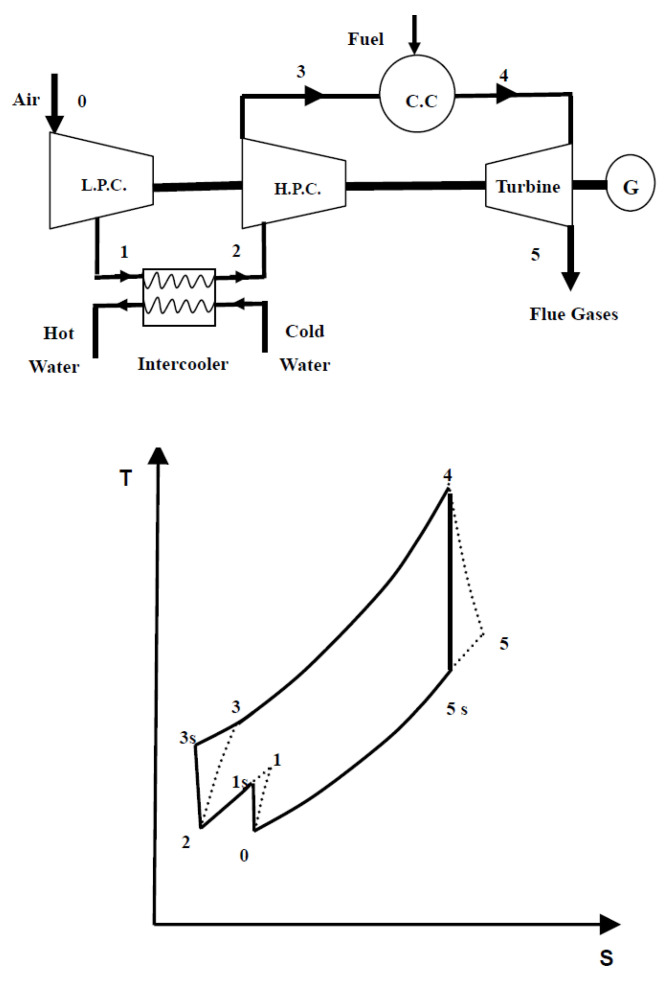
Schematic layout for the simulated intercooled gas turbine cycle [2].

Al-Doori [18] investigated the effect of using an intercooler on the parametric performance of a gas turbine power plant. He simulated the effects of design and operational parameters on power output performance factors such as input compressor work done, the rate of fuel consumption, and thermal efficiency of the plant cycle, including the comparative performance of gas turbine plants with and without intercoolers. It was found that adding an intercooler increased the plant’s power output by decreasing the work done by the input compressor; hence, the thermal efficiency of the gas turbine power plant was increased. The flow chart of the system and cycle as modeled by the author is shown in Figure 10. The system’s main components are a low-pressure compressor (LPC), high-pressure compressor (HPC), turbine, combustion chamber, and intercooler heat exchanger. There is no recuperator in the cycle.

**Figure 11:**
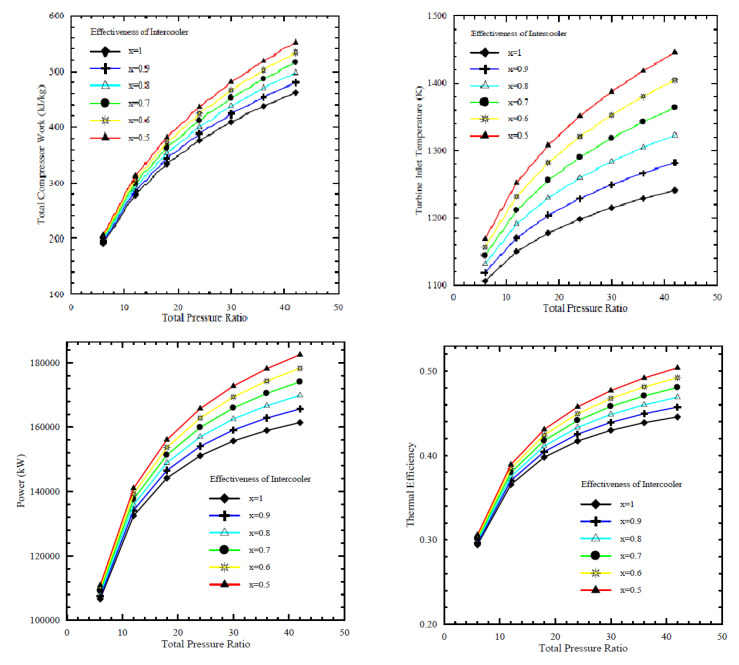
Effect of pressure on the main performance parameters of Brayton cycle with different values of effectiveness for the intercooler [18].

In this study, the Brayton operation cycle was analyzed based on the first law of thermodynamics. Affected parameters were simulated, compressor pressure ratios, ambient temperature, air-fuel ratio, turbine inlet temperature, and cycle peak temperature ratio. The performance of the heat exchanger (intercooler) was analyzed at different effectiveness values, 0.5, 0.6, 0.7, 0.8, 0.9, and 1, and the results are shown in Figure 11. As the effectiveness of the intercooler increased, the compressor’s total work decreased, but increasing the efficacy of the intercooler may reduce the power output and cycle thermal efficiency. Thus, it was recommended that the performance of intercooler effectiveness should be optimized. However, with an increase in compressor pressure ratio, the work done by the compressor is increased. Nevertheless, the thermal efficiency of the cycle is increased due to a significant increase in the power output of the turbine. It follows that the compression processes needs to be optimized.

**Figure 12:**
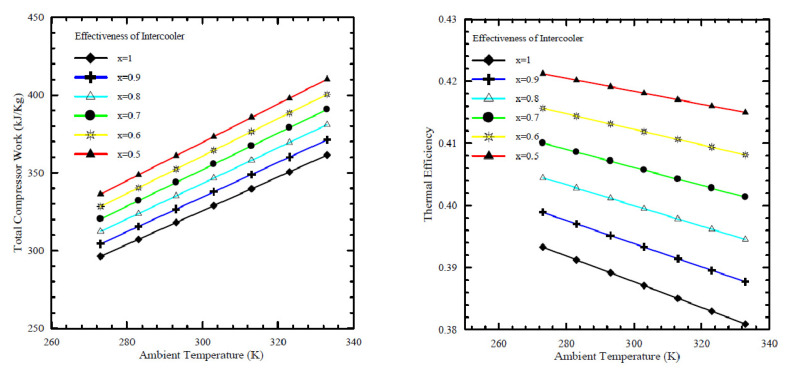
Effect of ambient air temperature to inlet compressor on the performance of Brayton cycle with different intercooler effectiveness [18].

Figure 12, which was obtained from Al-Doori [17], shows that an increase in ambient air temperature at the inlet to the compressor increases the work done by the compressor and decreases the thermal efficiency. It is also seen from the figure that the value of intercooler effectiveness is an important factor. It was concluded that increasing the cycle peak temperature and total pressure ratios can improve the performance of an intercooled gas turbine cycle. The results showed that using an intercooled gas turbine power plant can offer a fuel consumption of 8% better than a simple cycle gas turbine, with a 5–9% increase in power.

New composite materials can be used for manufacturing gas turbine components such as intercoolers, recuperators, compressors and turbine blades, etc. These have many advantages, such as low weight, increased toughness and strength, and can be used at very high temperatures [19]. The use of such material can decrease the mass of a plant by more than 60%. In Ref. [18], a recuperator heat exchanger was manufactured from a carbon/carbon matrix composite and used in a nuclear power plant. The aim was to simultaneously increase the efficiency of the gas Brayton cycle system, which incorporated a recuperator and substantially reduce the system’s mass as the recuperator accounted for nearly half the weight of the energy conversion system. Developing a lighter recuperator that provides better performance than current heat exchangers would prove hugely advantageous. The feasibility of a carbon-carbon (CIC) composite recuperator core was assessed, and a mass savings of 60% with a volume penalty of 20% were estimated. The excellent thermal properties, high-temperature capabilities, and low density of carbon-carbon materials make them attractive for use in the gas Brayton cycle system.

**Figure 13:**
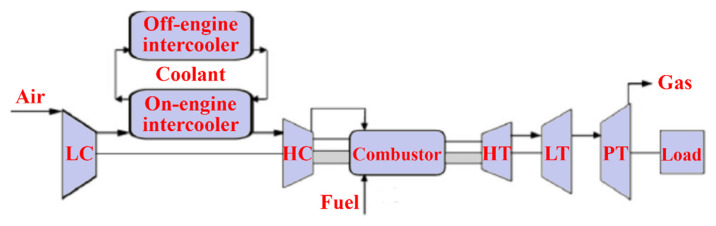
Intercooled-cycle gas turbine schematic diagram.

Wen and Dong [20] investigated intercooler heat transfer and fluid flow using numerical analysis. The study was carried out on an intercooled-cycle gas turbine applied to a marine system. The intercooler’s performance was investigated based on its design and operational parameters. The operation cycle was optimized as a real system. The research also included the thermodynamic calculations for the intercooled-cycle gas turbine, which are important for the design and selection of each component. The main components of the high-power marine intercooled-cycle gas turbine consisted of a three-axis gas turbine, heat exchanger, and propeller. LP, HC, combustor, high-pressure turbine (HT), low-pressure turbine (LT), and power turbine (PT) are the main components of the three-axis gas turbine. However, the heat exchanger device consists of two main sections, an on-engine intercooler, and an off-engine intercooler. The system included a reduction gearbox to connect the propeller to the PT, as shown in Figure 13. The dynamic performance of this kind of engine is mainly dictated by rotor inertia, volume inertia, the thermal inertia of the intercooler system, and the relative physical properties of working fluids used. The results showed that increasing the effectiveness of the intercooler led to a decrease in compressor work. Also, turbine work was decreased due to reduced turbine inlet temperature, decreasing thermal efficiency.

**Figure 14:**
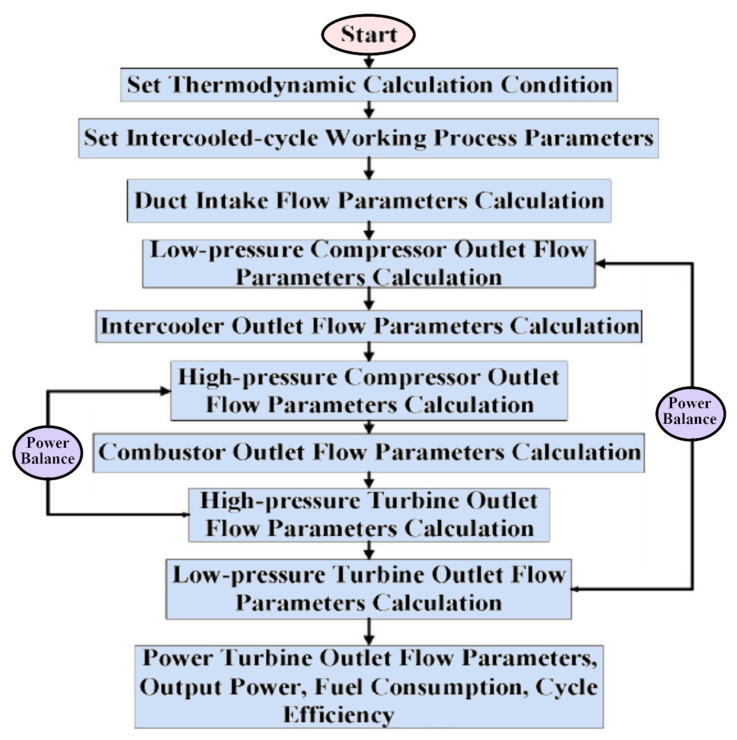
Thermodynamic cycle calculation process.

Ying et al. [21] investigated and optimized the operational parameters of a gas turbine intercooler system based on simulation and experiment. They analyzed the thermodynamic parameters and calculated performance factors for different marine gas turbines using an intercooled Brayton cycle by simulating the intercooled-cycle. The impact of environmental parameters and cold degrees of the intercooler was investigated because these factors significantly impacted this marine engine’s performance. The mathematical model of the intercooled-cycle gas turbine established in this research included relative non-linear simulations built on MATLAB/SIMULINK to perform parameter optimization. Given an intercooler of specific dimensions, the impact of different liquid-side flow parameters on system performance was analyzed based on the simulation model. The results indicated that an increase in flow rates of seawater and glycol-water solution could improve the performance of the intercooled-cycle gas turbine, but the ratio of the flow rates should be chosen according to the atmospheric conditions and seawater temperature according to the optimization rule that was derived. The heat is drawn from the LPC outlet air by the on-engine gas-liquid heat exchanger and transferred into the seawater by the off-engine liquid-liquid heat exchanger. Thus, the compressed air was cooled before entering the HC by an intercooler system, reducing HC power consumption. The total output power of the gas turbine was increased significantly. The relative thermodynamic calculation process is shown in Figure 14, this engine’s on-design parameters, and the software interface is based on MATLAB/GUI.

**Figure 15:**
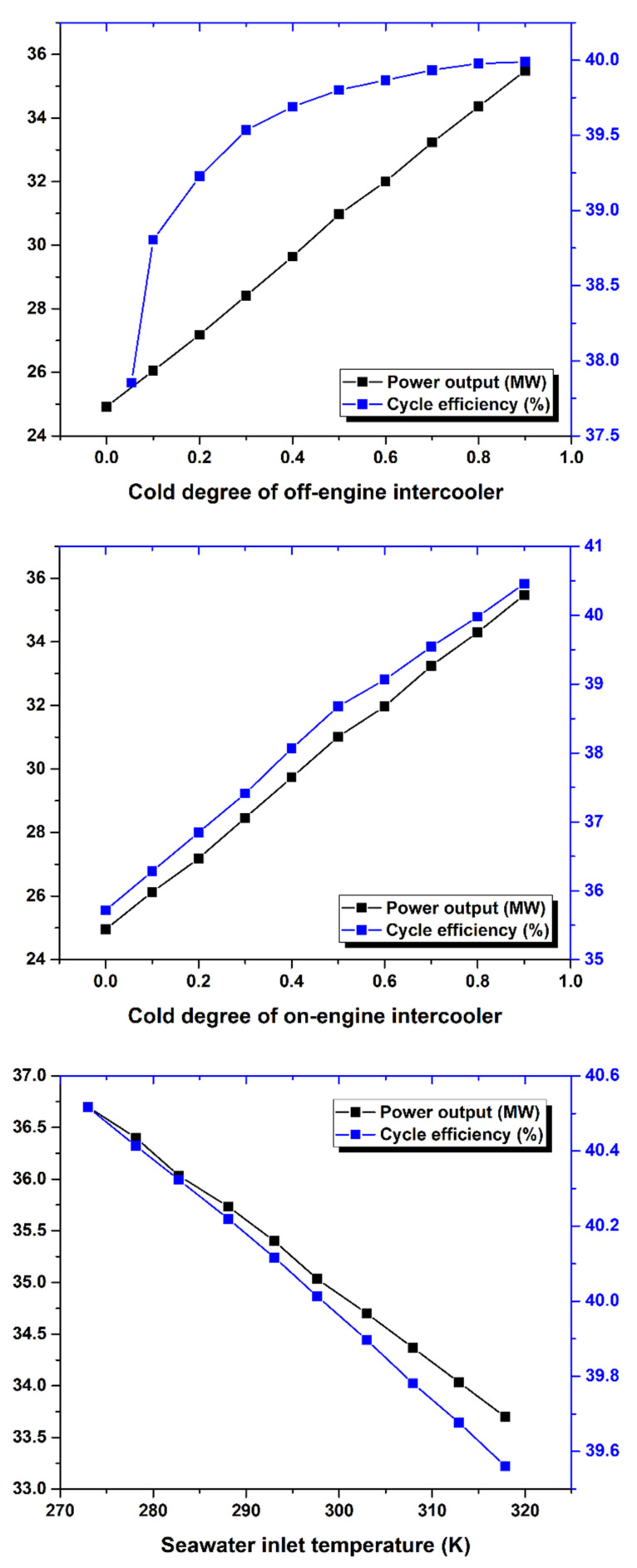
Results from Ying. et al. [21] research work.

As shown in Figure 15, the cold degree of the intercooler significantly affects the Brayton cycle of the marine gas turbine power plant. Increasing the cold degree of the on-engine intercooler led to an increase in power output; hence, the thermal efficiency was increased. However, increasing the seawater inlet temperature to intercooler led to decreased power output and cycle efficiency. So, the cold degree of the intercooler and inlet coolant fluid temperature are critical operating parameters because they directly affect the power output and thermal efficiency of the Brayton cycle.

**Figure 16:**
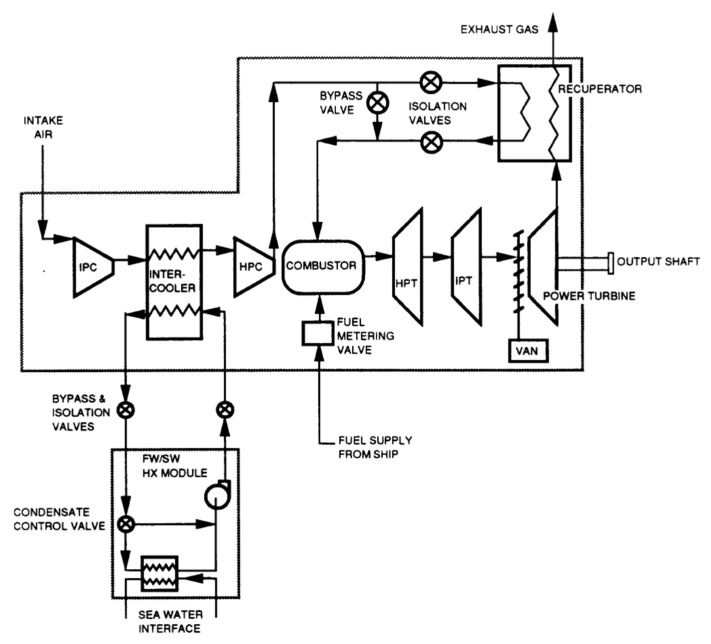
Shows the main parts of the WR-21 gas turbine engine flow chart [22]. IPC—Intermediate pressure compressor, HPC—High-pressure compressor, IPT—Intermediate pressure turbine, HPT—High-pressure turbine.

WR-21 is a gas turbine engine that provides a dense power package at high unit power ratings and is used in many marine applications for the propulsion of large surface vessels. Crisalli and Parker [22] presented an overview description of the WR-21 intercooled recuperated gas turbine engine system designed, built, and tested for the U.S. Navy Surface Fleet. The study developed a program for reducing operational risk and improving the engine’s performance and included an economic analysis of fuel used, and showed that a WR-21 engine reduced propulsion fuel costs, compared to simple cycle high powered engines, by up to 30%. The construction of the WR-21 system and its main parts, functions, and operations were described in the paper and are shown in Figure 16. A similar investigation was carried out by the English, who also discussed how the WR-21 engine was integrated into the Royal Navy Type 45 Destroyer. It was shown the WR-21 was considered an effective cycle gas turbine that could meet the requirements of both conventional propulsion and Integrated Electric Propulsion systems. This engine’s operational and most crucial advantage was presented as easy to use in most types of ship propulsion system configurations. Additionally, the WR-21 was declared to meet future warships’ high-power prime mover requirements and offer an efficient cruise/boost engine in one package. Compared directly to the use of simple cycle high power engines, the WR-21 was declared to provide significant fuel savings. Besides reducing costs, it offered operational advantages in ship range and speed [23].

The WR-21 engine used two primary heat exchangers to improve its performance, intercooler and recuperator devices (ICR). The engine consists of a two-spool gas generator with a free power turbine. Its operational theory was based on the Rolls-Royce RB211 three-spool aircraft engine technology. This marine engine used dual-loop freshwater/seawater intercooler heat exchangers, and a plate-fin recuperator. A digital control device was coupled and applied in the system because that gave much greater freedom to optimize the operation and maintenance of the vessel’s machinery. The main parts of the engine included the following items, see Figure 16: intermediate pressure compressor (IPC), intercooler, high-pressure compressor, combustor assembly, high-pressure turbine (HPT), intermediate pressure turbine (IPT), power turbine, and recuperator.

The WR-21 operation is based on cold air from the ambient environment entering the ship’s intake. It is pumped directly through the intermediate compressor (IPC), after which it enters the intercooler where the air is cooled, with heat from hot air transferred to the coolant (seawater). Thus, the SFC is improved directly if the compressed air exiting from HPC has gained a lot of heat while passing through the intercooler [21]. The cooled air with a higher density is then compressed by the high-pressure compressor (HPC) and passed to the recuperator. In the recuperator, the air is reheated using heat from the exhaust gases from the low-pressure stage of the turbine. This hot air then enters the combustor, mixed with fuel to make a mixture that burns quickly. After that, the hot gases exit from the combustor to first enter the high-pressure stage of the gas turbine (HP), then the intermediate stage (IP). In these two stages, useful work is generated directly, driving the HPC and IPC. Finally, the hot gases leave the low-pressure turbine, enter the variable air nozzle (VAN), and enter the power turbine which extracts useful work to drive the ship. Then, the hot gases exit from the power turbine, partly as exhaust gases which enter the recuperator to heat the compressed air before it enters the combustor, thus improving the SFC. Using the variable air nozzle (VAN) device in the WR-21 gas turbine helps maintain the temperature of gases high even after the turbine has extracted power.

A final conclusion presented by Crisalli and Parker [22] of their investigation of the WR-21 was that performance requirements could be combined to explain how trade-offs could lead to reaching the best overall combination of cost, performance, reliability, and maintainability.

Arai et al. [24] analyzed the performance of a micro gas turbine system using computational fluid dynamics (CFD). They optimized the operational parameters such as the gas flow inside a compressor and a turbine, to achieve high performance. This included optimizing the angle of the impeller blades of the compressor. The results from the numerical analysis indicated that, in their micro gas turbine, the total pressure ratio of the compressor was improved by 30% compared to the same system without the change. Optimizing the angle of the impeller blades directly affected the power extracted from the gas, improving the power output of the cycle, increasing the thermal efficiency, and decreasing the specific fuel consumption.

**Figure 17:**
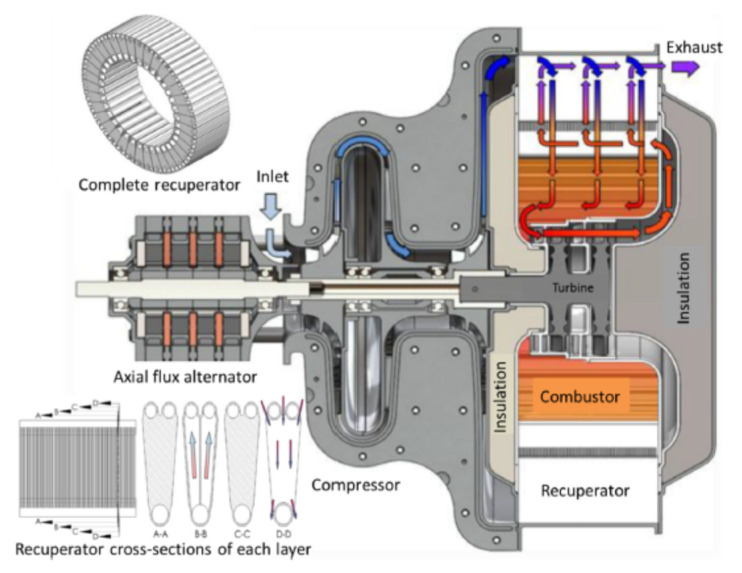
Cross-section of the recuperated ceramic turboshaft engine [25].

The increasing demand for highly efficient, environmentally friendly aero-engines using gas turbine Brayton cycle to recover heat from the exhaust gases through a heat exchanger is important. An efficient recuperator for aircraft engines was designed and optimized by Zhang and Gümmer [25]. The gas turbine blades can work at very high temperatures, reducing fuel consumption, emission gases, and noise, which are essential for rotorcraft power plants. The thermal efficiency reached was more than 60%. The designed recuperator has been manufactured with a ceramic plate-fin heat exchanger, as shown in Figure 17. The recuperator consists of three main parts: a tubular part, a primary surface, and a plate-fin, a type of recuperator suitable for application to rotorcraft technologies. The study concluded that while incorporating recuperators into gas turbine systems has long been considered for aircraft applications, significant advances in heat exchanger technologies are now possible based on using unique materials such as ceramics, superalloys, and composites, which can work in very high gas temperatures and severe thermal environments are available. However, the cost of these materials is relatively high compared to commercially used stainless steel 347. Concerning cost and creep-strength, modified in coloy alloy 803 and Haynes HR-120 seem to be the best alternatives up to 750 °C.

**Figure 18:**
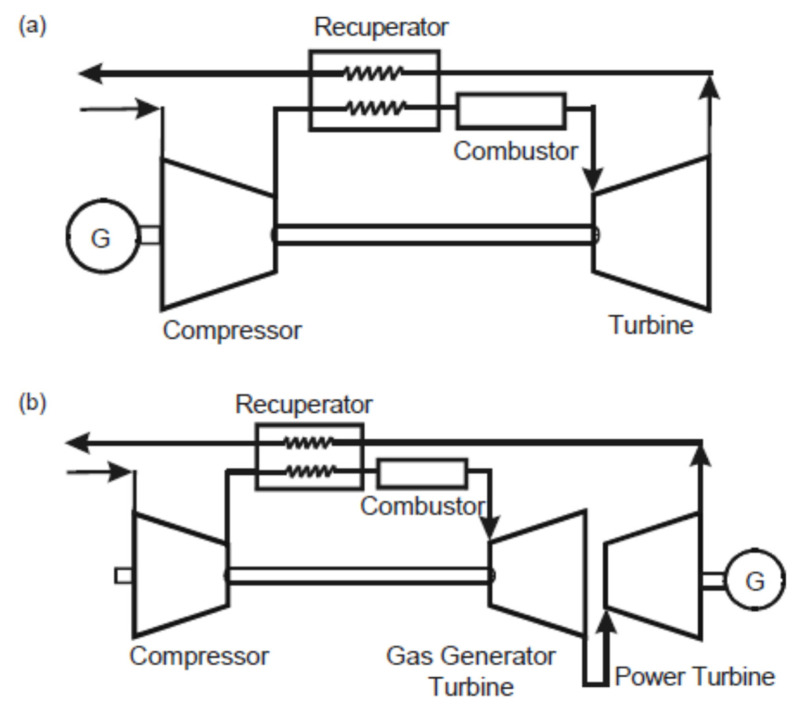
Schematic diagram of the recuperated gas turbine: (**a**) single-shaft. (**b**) Two-shaft, [23].

Most designers of gas turbines try to increase the thermal efficiency of the Brayton cycle by decreasing the fuel consumption by using regenerative cycle gas turbines due to their great potential for improving thermal efficiency. Kim and Hwang [23] analyzed the performance of recuperated gas turbines considering engine configuration and operational strategy. The study was based on increasing the heat recovery from exhaust gases by using a recuperator device for turbines under part-load. The recuperator was a surface plate-type heat exchanger, and Figure 18 shows it in combination with both a single-shaft and a two-shaft configuration. Recuperated gas turbines of both types were considered in this study.

Thermodynamic principles determine the performance of a gas turbine based on the design and operation of the heat exchangers used as recuperators, intercoolers, and gas turbine blades. The heat recovery process in the recuperator is based on its effectiveness and characteristic operation parameters, with utilization of the heat recovery effect during part-load operation being very effective. Therefore, the recuperator cycle may possess similar characteristics to the combined gas turbine/steam turbine cycle, which also employs heat recovery at the exit of the gas turbine.

Ferreira and Nascimento [26] used numerical simulation to investigate the performance of a gas turbine driven by biomass fuel. They computationally simulated the design parameters and performance of simple cycle gas turbine engines with one and two shafts. The study assessed the off-design behavior and performance of the gas turbine engine without accounting for the auxiliary syngas fuel compressor when the gasifier was at atmospheric. The results showed that the performance changed when the engine ran on syngas, with the running line in the compressor characteristic moved towards the surge line.

**Figure 19:**
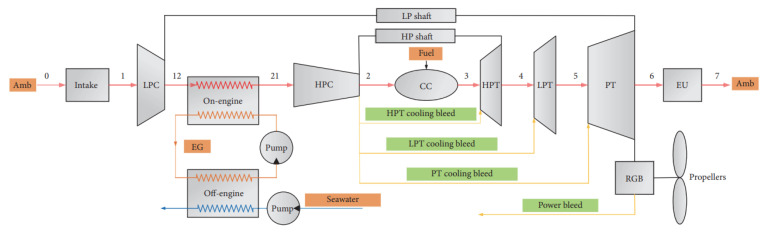
Schematic flow chart of intercooled gas turbine used for marine applications [27].

The intercooled gas turbine equipment can be installed into an existing gas turbine as an indirect heat exchanger. This is considered one of the main methods by which develop high-power marine power units. Ji et al. [27] presented an analysis of a simplified coupled nonlinear system, see Figure 19, and developed a new model for the steady-state of a marine intercooled gas turbine that can be used for simulating off-design performance.

Important variables considered included the scheduling of the ships’ missions, operational flexibility, and mechanical and thermal constraints. The operating diagram of the intercooled gas turbine was optimized using a three-dimensional graphical method. The resulting operating curves revealed that the control strategy at steady-state conditions for the intercooled gas turbine should be variable cycle control [27].

Soares [28] experimentally investigated the factors affecting the WR-21 operation cycle, including ambient temperature, pressure ratio, air inlet temperature, and air mass flow rate. He employed a special control algorithm for analyzing the performance of the intercooler. Generally, he recommended that the coolest air temperature should be maintained above the calculated saturation temperature by controlling the degree of cooling. The study indicated that the exit temperature of the intercooler was increased by reducing the LP compressor surge margin due to decreased mass flow. However, reducing the intercooling reduces the HP compressor speed, and hence the mass flow through the HP compressor is reduced. The power required by the HP compressor is directly proportional to its absolute inlet temperature and decreases as the temperature is decreased.

**Figure 20:**
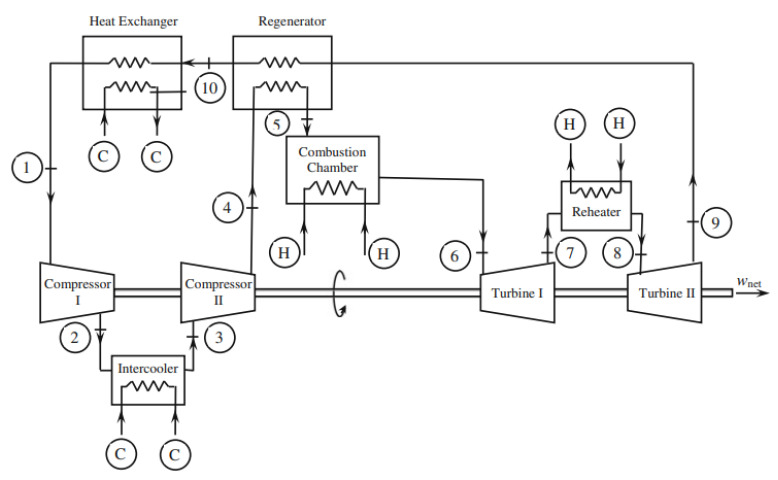
Schematic diagram of a realistic irreversible, regenerative, and reheat Brayton cycle [29].

Maher et al. [29] optimized the parametric performance characterization of an irreversible gas turbine Brayton cycle. They developed a mathematical model based on thermodynamic fundamentals and applied it to a Brayton cycle with a two-stage compressor, intercooler, regenerator, and reheat. The flow chart of the system used is shown in Figure 20. The exclusive effect of each of the operating parameters on each of the performance parameters is given in a general mathematical formulation that is applicable regardless of the values of the rest of the operating parameters and under any condition of operation of the cycle. The operating range of some of the parameters are as follows: minimum cycle temperature ranges between 302 and 315 K, maximum cycle temperature ranges between 1320 and 1360 K, maximum cycle pressure ranges between 1.449 and 2.830 MPa, and conductance of the heat exchanger ranges between 20.7 and 29.6 kW/K.

The different formulas for estimating work output, thermal efficiency, heat input, and entropy generation number in the Brayton cycle with a recuperator can be derived based on thermodynamic principles and given as functions of input factors such as pressure ratio, temperature ratio, recuperator effectiveness, turbine and compressor isentropic efficiencies, pressure drop in the combustor and recuperator and the ratio of specific heat capacities, see Živić, et al. [30]. The formulas based on the pressure ratios and intercooler effectiveness maximize or minimize other performance factors. There were three optimization criteria used in the study: maximum work output, maximum thermal efficiency of the cycle, and maximum gas flow rate. In the case of maximum thermal efficiency, when the recuperator effectiveness is lower than a certain limit, the Brayton cycle is better without a recuperator than with it. In the case of the other two criteria, the Brayton cycle with a recuperator is always better than without it, regardless of the value of the recuperator effectiveness.

A summary of what has been reviewed is presented in Table 3.

## 4. Heat Exchangers

In the previous section, the factors affecting the efficiency of gas turbines were investigated and it was shown that the presence of an intercooler and the condition of the air entering the turbine affect turbine efficiency. One way to increase the efficiency of a gas turbine is to reduce the inlet air temperature, which can be done by intercoolers, which are, essentially, heat exchangers. This section presents the principles governing the general operation of heat exchangers. Then the heat exchangers used in intercoolers, including the shell-and-tube, and plate-fin are reviewed. How to increase the thermal efficiency of heat exchangers is discussed, though using nanofluids as the working fluid is left to Section 5. The thermal design of shell-and-tube heat exchangers can be carried out using sophisticated computer software, but a good understanding of the underlying physical principle of heat exchangers design is needed to use the software effectively [31].

### 4.1. General Operation Theory of Heat Exchangers

**Figure 21:**
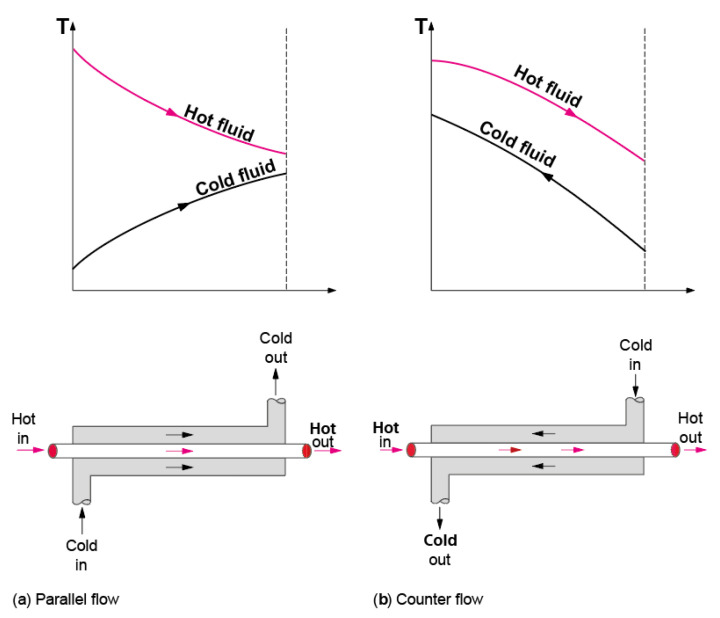
How a simple heat exchanger works.

A heat exchanger (HE) is a tool that transfers heat from one fluid medium to another and is considered an essential component in most heat engines and pumps. The heat exchanger transfers heat without transferring the fluid that carries the heat. The working fluids in heat exchangers may be liquid-to-liquid or gas-to-gas (single-phase) or liquid-to-gas, or gas-to-liquid (two-phase). Of course, heat exchangers can be used where each fluid is two-phase. Generally, the use of efficient heat exchangers leads to the high thermal efficiency of a process. Figure 21 shows the fundamentals of the operation of the heat exchanger.

The efficient heat exchanger (HE) improves the efficiency of machines and decreases energy losses to the surrounding. The most common types of heat exchangers are tube in tube, helical tubular, shell-and-tube, header tube, plate, fin–tube, plate-fin tube, and micro-fin tube heat exchangers. There are many applications of HEs, from power plant condensers and cooling towers to a recuperator in an industrial Brayton cycle, and domestic refrigeration and air conditioning systems [32]. With gas turbine power plants the main applications are intercoolers, recuperators or regenerators, indirect heat exchangers, and the transfer of heat between two flows of working fluids, separated by an impervious wall. A common example of an indirect heat exchanger is an automobile radiator.

The energy consumption in most energy conversion machines is based on their performance using HEs. Generally, the energy consumption of a heat engine can be reduced by selecting an efficient heat exchanger to be used with it. For example, if the boiler’s thermal efficiency in an industrial steam power plant increased 0.5%, this would save considerable amounts of conventional fuel (oil) [33]. There are many studies on the analysis and design of heat exchangers, including heat transfer due to condensation or evaporation. Most investigations have been concerned with the design and simulation of different types of heat exchangers such as tube in tube and shell-and-tube. The aim was to improve the performance of the heat exchangers by such means as using fins and micro-fins. The theoretical simulations were compared to experimental data from numerous articles. These data were used to assess the theoretical models and analytical solutions for linear and non-linear heat transfer problems. Such design and development of finned–tube in tube coil heat exchanger were carried out by, for example, Kakac et al. [34].

In most HE designs, the temperature gradient, the rato of temperature difference to wall thickness (∆T/∆x), is critical. The temperature gradient determines the heat transfer rate and heat flux through the HE. In the given ambient conditions, the correct selection of temperature gradient and pressure drop across the HE maximizes the heat exchanged between the two sides of a heat exchanger, between the two working mediums [34].

The heat transfer area also has a big effect on the performance of the HE. The total heat transfer rate depends directly on the surface area of the HE separating the two fluids. Increasing heat transfer area will invariably increase the heat transfer rate. Thus, in heat exchangers maximizing the overall heat transfer rate means increasing the heat transfer area, which can be achieved by, for example, installing fins on heat exchanger tubes. Such an arrangement permits compact heat exchangers with very large heat transfer areas per unit volume, with a corresponding reduction in volume, weight, footprint, support structure needed, and even cost. In addition, there could be an improved process design, plant layout, and processing conditions, together with lower fluid inventory [35].

**Figure 22:**
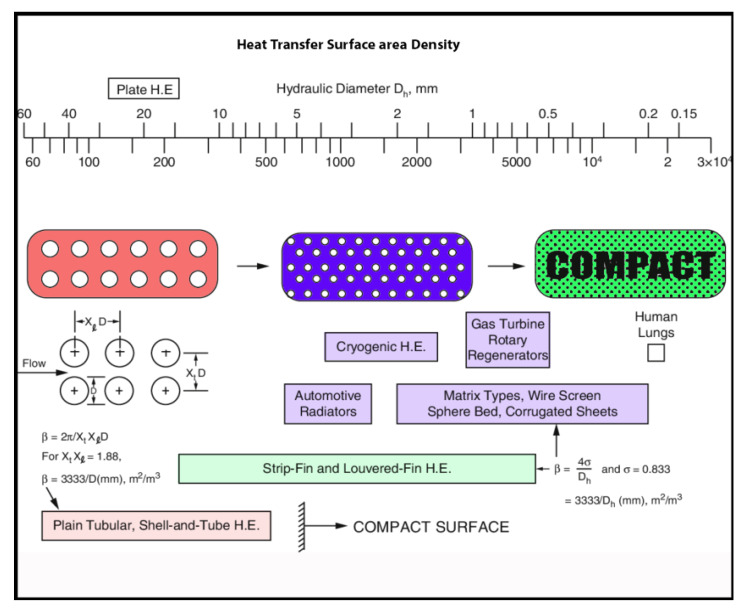
Heat transfer surface area density spectrum of exchanger surfaces.

Generally, gas-to-liquid heat exchangers are compact. The heat transfer surface has a surface area density greater than 700 m^2^/m^3^ and greater than 400 m^2^/m^3^ when operating in a liquid or phase-change stream. The hydraulic diameter, D_h_, defined as 4Ao /P (where Ao is the minimum free-flow area on one fluid side of a heat exchanger, and P is the wetted perimeter of flow passages on that side [32], can be less than 6mm (1/4 in.) for gas streams. Figure 22 shows the relationship between surface area density and hydraulic diameter of most types of heat exchangers. As the surface area density increases, the hydraulic diameter increases, and then the overall dimensions and mass of the applied HE equipment increases [35].

The fluid flow in the channels of exchangers is an essential factor when considering heat transfer coefficients. Generally, the fluid flow rate directly affects the local heat transfer coefficients, the heat capacity increases as mass flow rate increases and decreases as mass flow rate decreases. As an example, in the case of the plate heat exchanger, which has about twice the average heat transfer coefficient (h) on one fluid side or the average overall heat transfer coefficient (U) than that for a shell-and-tube exchanger for water/water applications.

The local heat transfer coefficient (h) is based on the properties and phase of the working medium on both sides of the HE. In the case of gases, the heat transfer coefficient h is generally one or two magnitudes lower than for liquids. But to maximize the heat transfer and minimize the size and weight of a gas-to-liquid heat exchanger, (h × A) values should be the same on both sides of gas-to-liquid and gas-to-phase change HEs. Fortunately, the gas-side heat transfer surface can be made very large especially in the case of circular tubes as commonly used in shell-and-tube exchangers, by using compact extended-surfaces (e.g., plate-fin and tube-fin). In fact different orders of magnitude of surface area are available providing greater flexibility in distributed surface area on the hot and cold sides, meaning lower cost, weight, volume, pressure drop, and driving power are needed.

Low cost, low weight, small size, high efficiency, and high performance are considered the most desirable parameters when designing heat exchangers.

### 4.2. Shell-and-Tube Heat Exchangers

**Figure 23:**
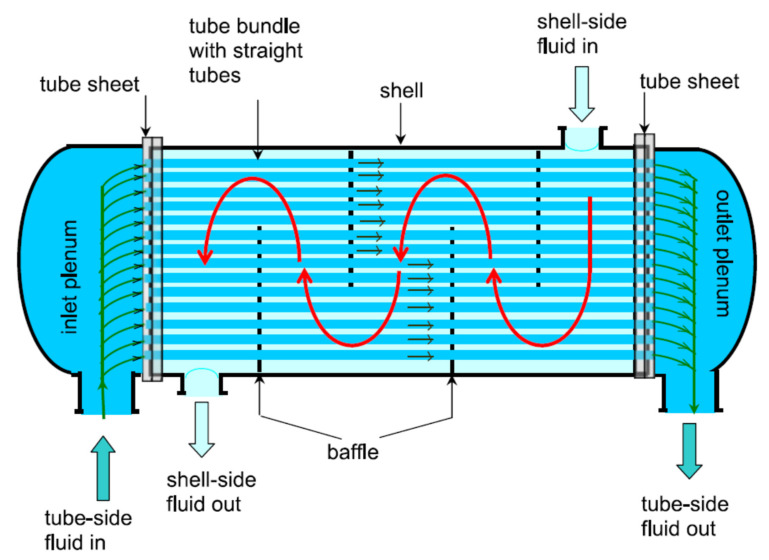
Shows one pass counter-flow shell-and-tube heat exchanger.

A shell-and-tube heat exchanger is a device in which heat is transferred from one fluid stream to another via an intervening solid wall and is a common design of HE. It is widely used in many applications, from large chemical processes to air conditioning systems. Shell-and-tube HEs are commonly used in gas turbine power plants as recuperators, consisting of a tube bundle enclosed in a cylindrical shell, see Figure 23. The principal components of a shell-and-tube heat exchanger are shell, tubes, channel, channel cover, baffles, and tube sheets. One fluid flow through the tubes, and a second flows through the shell, and heat is transferred between the two [36].

The shell-and-tube heat exchanger consists of a series of tubes within a larger volume (typically cylindrical but can be any shape). Ling et al. [36] have presented design guidelines covering the selection and sizing methods for heat exchangers, listing all the important factors that must be considered and the suitability of different types used in industrial processes. However, the study concentrated on two types of heat exchangers only. Figure 23 shows a counter flow design, but co-flow designs are also possible, as are multiple pass designs in which the inner fluid enters and leaves at the same plane with a 180-degree bend at the other, forming a co-flow/counterflow (or vice versa) design.

**Figure 24:**
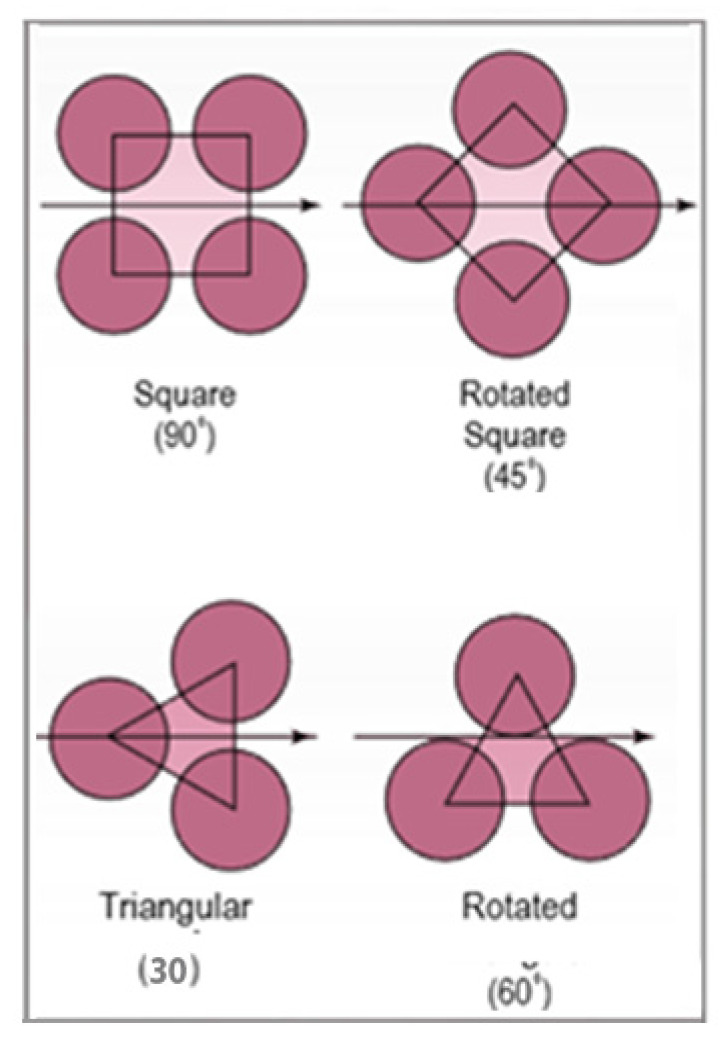
Typical tube layout patterns.

Mukherjee carried out a design study of the effectiveness of shell-and-tube heat exchangers [36], detailing the main components, classifications, and design data. In addition, he described the design procedure for the shell, tubes, tube pitch, baffles, and layout patterns. There are two common tube layout patterns, as shown in Figure 24. Triangular patterns can accommodate more tubes within a shell than square.

Baffles support the tubes and maintain the desired flow velocity for the shell side fluid [37]. There are many types of baffles, plates, and rods, with plate, baffles the most common. These may be single, double, or even triple-segmental.

Different factors affect the performance of shell-and-tube heat exchangers. These include such factors as ambient temperature, working fluid mass flow rate, fluid used, Reynolds number (flow velocity), temperature difference, and tube wall thickness, thermal conductivity and surface area. Also important are fluid flow patterns (parallel, counter, cross-flow). The hot fluid usually flows through the tubes inside the shell, which carries the flow of cooler fluid in the opposite direction (counter flow). Ambient conditions and operational factors may affect the heat transfer coefficients in real heat exchangers.

**Figure 25:**
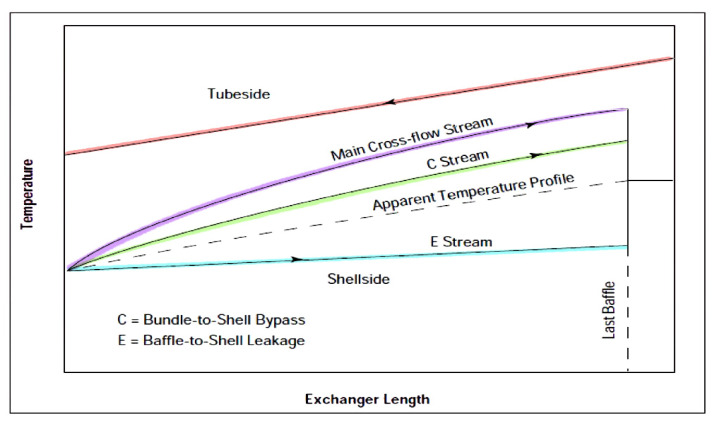
Temperature profile distortion factor due to bypass and leakage.

An important parameter to be taken into consideration is temperature profile distortion. Practically, it has been noted that there are leakage and bypass streams in shell-and-tube HEs with less efficient heat transfer than the main cross-flow stream, reducing the overall heat transfer rate and lowering the temperature profile, as shown in Figure 25. The temperature profiles of the baffle-hole-to-tube leakage, shell-to-bundle leakage, and pass-partition bypass streams will be based on their respective flow leakage fractions, and the fractional heat-transfer area encountered [38].

Suri et al. [39] reviewed and summarized the results of investigations into various types of operation and performance of heat exchangers with emphasis on studies of roughness elements used to enhance heat transfer rate. It was noted that many experimental and analytical studies in the literature concentrated on the analysis of heat transfer coefficient and pressure drop optimization with most investigators comparing and/or analyzed the thermal-hydraulic performance of different shaped heat exchanger tubes. The study helped select the best roughness shapes for increased heat transfer rates and reduced pressure drop losses.

Lunsford [40] investigated practical methods of increasing the performance of shell-and-tube HEs, considering whether the HE had been sized correctly, was performing to specification, and the effect of fouling factors. If a larger pressure drop across the HE was permitted, the heat transfer rate could be enhanced by using augmented surfaces.

Different practical models show how commercial simulation programs and shell-and-tube exchanger rating programs are used to evaluate HE performance problems, including fouling effects. Zueco et al. [41] analyzed and improved the performance of the shell-and-tube heat exchanger using educational software. The program developed used the LMTD and effectiveness NTU methods and may prove pedagogically valuable for engineering and students working in the field of design and manufacture of HEs. The software was developed for use in the design of shell-and-tube heat exchangers. It was tested on practical thermal problems using an ordinary PC and provided accurate results directly and effectively.

**Figure 26:**
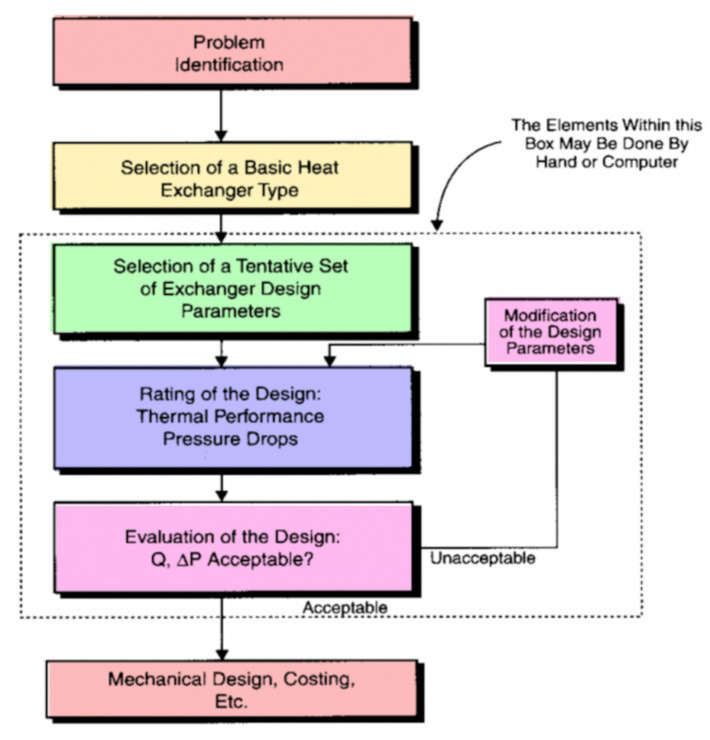
Shows a flow chart for the thermal design of HEs.

Edwards [42] designed and tested different styles of shell-and-tube HEs to produce a concise review of the critical issues involved in their design. He analyzed the thermal design of a shell-and-tube heat exchanger, considering many interacting design parameters such as type of flow, tube arrangement, tube diameter, thickness and length, and shell size. He investigated the effect of those parameters on effectiveness, pressure drop, heat transfer coefficients, heat transfer rate, and fouling rate. The study was carried out numerically using a PC. He claimed that the flow chart shown in Figure 26 could be used for the thermal analysis of HEs.

Bouhairie et al. [43] used ANSYS FLUENT to optimize and analyze the thermal design of a shell-and-tube heat exchanger. The study focused on the flow behavior in the window between baffle compartments to reveal the complexity of shell-side flow dynamics around the tube bundle. The adequacy of the various turbulence models in predicting the window flow field was also evaluated. Three-dimensional, computational fluid dynamic (CFD) simulations of adiabatic flow in a periodic section of the exchanger were conducted. The numerical results were compared to particle image velocimetry measurements in the window region where tubes were absent.

Ghaith et al. [44] investigated how to enhance the thermal design of a shell-and-tube heat exchanger. The study aimed to improve the performance of an industrial shell-and-tube heat exchanger used for cooling raw natural gas, to provide an optimum and reliable thermal design of a single-shelled finned tube HE, to replace the existing two shell-and-tube HE due to space limitations in the plant. The thermal design was based on the effectiveness-NTU method. The shell-side and tube-side overall heat transfer coefficients were determined using the Bell-Delaware method and Dittus-Boelter correlation, respectively. The results showed that the critical area to provide a thermal duty of 1.4 MW was about 1132 m^2^ with tube-side and shell-side heat transfer coefficients of 950 W/m^2^K and 495 W/m^2^K, respectively. The results generated from the mathematical model, using HTRI software, showed a good match in terms of the heat transfer area and tube-side heat transfer coefficient.

Wan et al. [45] investigated thermal failures generated in the tube-to-tube sheet joints of a shell-and-tube heat exchanger, which are greatly affected by residual weld stresses. To ensure structural integrity, it is very important to predict and decrease the residual stresses in the joints between tubes and tube sheets of HEs. X-ray diffraction and finite element method were combined to analyze the residual stress distribution in the tube-to-tube sheet joints and showed that large tensile residual stresses above yield strength could be generated. The residual stresses at the bottom surface and the edge of the tube sheet are relatively small, even becoming compressive. The residual stresses increase as the heat input increases. The duplex welding method was recommended to decrease the residual stress.

Wang et al. [46] conducted research to provide a state-of-the-art development of three kinds of surface heat exchangers: shell-and-tube with helical baffles, primary surface, and air-cooled as used in large air-cooled systems. The research highlighted that shell-and-tube heat exchangers that dominate the market are available in many configurations, including exchangers with segmental baffles. However, there were drawbacks associated with this geometry, including inefficiencies, leading to the development of the shell-and-tube heat exchanger with helical baffles. The basic principle of this design is relatively simple, involving circular or elliptical sector-shaped plates which are arranged in a pseudo-helical baffle system. In this setup, each baffle occupies one quadrant of the heat exchanger shell cross-section area, with an angle of inclination towards the central axis of the shell, resulting in a helix flow path for the working fluid.

### 4.3. Plate-Fin Heat Exchangers

Examples of compact heat exchangers are plate-fin, tube-fin, and rotary regenerators used with gas flow as one or both fluids. Today, printed-circuit heat exchangers are examples of compact heat exchangers used with liquid flows. Compact heat exchangers are not necessarily of small bulk and mass, but are much smaller than conventional heat exchangers of corresponding performance because they incorporate surfaces of high-surface-area density. Compact heat exchangers are widely-used when there is a need to achieve a large heat transfer rate per unit volume. The small flow passages invariably mean the flow will be laminar [47].

The mass flow through plate-fin heat exchangers must be modified and optimized according to the operating conditions and specifications of the heat exchanger used. Increasing the flow rate of cold fluid in the plate-fin tube heat exchanger leads to an increase in both heat transfer rate and pressure drop across the heat exchanger. Using a heat exchanger with offset strip fin geometry is considered one device to obtain the highest performing heat exchange surface, but this is complex to manufacture due to the very difficult brazing process.

In the case of using highly compact HEs, the final shape must be designed to achieve uniform flow distribution through a large number of passages [35]. It should have a large frontal surface area, low flow length, and low header path. When selecting compact HEs, there are some important design and operating factors to be considered: invariably, at least one of the fluids will be a gas which means these heat exchangers have a low D_h_, the fluid pumping power, and the pressure drop (∆p) are very important as this will affect heat transfer rate and power consumption of the system, temperatures are limited compared to shell-and-tube exchangers. The working fluids must be clean and noncorrosive because a big problem with compact heat exchangers is fouling, the formation and accumulation of scale on the paths of fluids, leading to decreased heat transfer rate and an increase in the pumping force required. Fouling is also significant in plate-tube fin heat exchangers, especially with very fine circular and noncircular fins. Highly flammable or toxic fluids must be avoided due to the possibility of leakage. Non-fouling fluids, such as clean air or gases, light hydrocarbons, and refrigerants, are recommended for these HEs. The problem is compounded because mechanical cleaning is very difficult. Chemical cleaning is one practical solution, as is thermal baking with subsequent rinsing, particularly in the case of very small units. This precludes the use of compact plate-fin heat exchangers in heavy fouling conditions.

The selection of proper materials for constructing a recuperator is very important because of the required heat transfer, mechanical expansion, chemical reactions, etc., that occur when working at very high temperatures. The effect of oxidization of water vapor in the recuperator plays a crucial role affecting the heat transfer coefficient of the HE. Pint et al. [48] studied experimentally the effect of the water vapor found in exhaust gases on the oxidation resistance of materials commonly used for recuperators, stainless steel (SS) grade 321, Inconel 625, Haynes 214, and PM2000. The study found that SS321 had relatively low oxidation resistance at a temperature of 700 °C and recommended the use of stainless steel material of HE only for hot fluid inlet temperatures of less than about 675 °C. The report recommended using very much more expensive superalloys for manufacturing those heat recuperators used at very high temperatures or used with water on either side to minimize overall operating costs.

Lara-Curzio et al. [49] designed and built a recuperator that operated at a temperature of 843 °C and was used with a micro-turbine of 60 kW rated output. Different materials and alloys were tested and subjected to high thermal stresses, and the material selected was a superalloy of aluminum mica, i.e., the final material selected for manufacturing microturbines was a ceramic.

There is a strong relationship between the material of a heat exchanger and its performance. For HEs operating at more normal conditions, the high thermal conductance required demands the use of materials such as copper, aluminum, iron alloys, etc., which have both the high thermal conductivity and mechanical strength required. These materials should also be resistant to chemical degradation and wear. Cost and manufacturing processes are two important factors that affect the selection of materials from which HEs are made.

The plate HE can be used with gas/liquid working fluids. The gas medium may be pure air, and the liquid can be water, oil, or any other liquid. Fabio et al. [50] modeled and designed ceramic plate HEs with special features for use in different applications, manufacturing the HEs using a welding process. These ceramic plate HEs achieved a very high thermal effectiveness, reaching 95%. The investigation also explored working fluids and recommended that the working fluid should be of low viscosity to decrease the pump power needed for circulation. However, it was also found that the very smooth material used for the plate HE decreased the pressure drop and reduced flow distribution problems, allowing a working fluid of greater viscosity.

Modern plate HEs, as described above, are assembled as a single unit, forming criss-cross flow channels of intricate geometry, characterized by the formation of contact points between the opposite walls located at corrugation crossings. The effectiveness of such a plate HE is due to the plates’ corrugated form, which has a major impact on heat transfer and hydraulic behavior. Arsenyeva et al. [51] investigated the design of an efficient plate HE by analyzing heat transfer within channels of complex geometry typical of those formed by thin metal plates with the aim of predicting the influence of pressure drop, temperature, and heat load on the performance of the HE. It was found that the geometry of the corrugations had a major impact on the heat transfer process. The study concluded that it is difficult to achieve optimal performance with the plates currently on the market and as such, further research is required into the design of these exchangers.

Bichnevicius et al. [52] investigated the performance of a louvered plate-fin heat exchanger, comparing traditionally manufactured and additively manufactured HEs based on the same design. The additively manufactured HEs were produced by a laser-based powder bed fusion process, also known as selective laser melting. The study found additively manufactured HEs varied in performance even when printed using the same digital model. It was concluded that there is a need to allow for variation in surface roughness, geometric deviations, and other potential defects when designing HEs, resulting in improved thermal performance of future heat exchangers produced in this way.

Plate-fin geometry for maximizing heat transfer through HEs was investigated by Karvinen and Karvinen [53]. They presented a new method for determining plate-fin geometry for maximizing heat flux. The method is based on approximate analytical solutions of conjugated heat transfer correlations. The result showed that certain thermal and geometrical properties of the fin and the flow are very important factors due to their direct effect on the size of the HE. The values of heat transfer coefficients were given for rectangular, convex parabolic, triangular, and concave parabolic fin shapes for natural and forced convection, including laminar and turbulent boundary layers. It was concluded that there was no need to evaluate convection heat transfer coefficients separately because they are already included in these variables. Easy-to-use design rules were presented for finding the geometry of fixed volume fins that gave the maximum heat transfer.

The heat transfer performance and space efficiency of compact heat exchangers with an emphasis on wavy fin exchangers was investigated by Kim and Rhee [54]. These heat exchangers typically have high thermal performance due to the dynamic flow pattern imposed by the fin geometry. This is influenced by the corrugation angle, which affects the heat transfer effectiveness and the pressure drop depending on the flow, which can be steady laminar, unsteady laminar, or turbulent. This was a numerical study to determine the optimum cross-cut length of wavy fin heat exchangers with various corrugation angles but with a fixed space ratio and a steady laminar flow of the working fluid, water. The research found that the optimum cross-cut length for wavy fin heat exchangers varied with the corrugation angle and concluded that an increase of corrugation angle had a negative impact on the overall thermal performance in laminar flow. In addition, it was also found that there was an increasing pressure drop as the corrugation angle increased.

Manglik et al. [55] investigated the effect of fins on the performance of a fin plate HE, how the fin geometry affected the laminar forced convection of air in the inter-fin passages. The study was carried out for steady-state laminar flows conditions. The authors investigated different configurations of HEs such as rectangular, trapezoidal, and triangular plate-fin channels. The aim was for the fins’ presence to increase the HE’s performance rate by increasing the heat transfer area.

Some fin arrangements can result in a reduction in convective heat transfer coefficient, as in the case of low fin density cores with longer fins of relatively low-conductivity material (e.g., stainless steel). However, with increasing fin density and shorter fins, the convection performance is virtually the same as that with 100% fin efficiency; the same is the case when fins are made of very high conductivity metal such as copper. These results provide design insights for optimizing the conjugate fin-conduction and fluid-flow convection performance in plate-fin compact heat exchangers.

Han et al. [56] numerically investigated fin-and-tube heat exchanger performance focusing on fluid flow and heat transfer characteristics using oval and circular tubes. The study involved mathematical simulations of three tubes, each with wavy fins and louvered fins. The study aimed to ascertain the effect of different shaped tubes on the fluid flow and the heat transfer characteristics of finned tubes. The study concluded that an oval tube with fins exhibits more efficient heat transfer performance with lower pressure loss because of improved flow characteristics. The study also found that the use of fins with a larger circular tube gave higher heat transfer coefficients for a smaller total heat transfer area. The authors also found that the heat transfer rate of the smaller circular tube with fins was better than for the larger circular tube with wavy fins. The overall conclusion was that an oval fin-tube reduced flow resistance and improved the heat transfer capacity of the heat exchangers, with a corresponding improvement in fin efficiency. For example, the heat transfer rate of the oval tube with fin was up to 4.95% better than the larger circular tube with louvered fin, while the pressure loss decreased by up to 31.8%. The study also found that the fins with the larger circular tube had higher heat transfer coefficients but smaller total heat transfer area. The heat transfer rate of the louvered fin indicated that the heat transfer coefficient was more important than the heat transfer area.

Nasrabadi et al. [57] numerically investigated the design of plate-fin HEs with consideration of industry applications, using a new design program based on MATLAB. This heat exchanger is the main component in micro gas turbines as a recuperator to improve the thermal efficiency of the microturbine by up to 30%. The paper presents a literature survey of different types of gas-to-gas heat exchangers, emphasizing fin-tube, fin-plate, shell-and-tube heat exchangers, and regenerators. The effect of thermo-hydraulic parameters on the efficiency of the three heat exchangers was also investigated. The effects of these heat exchangers on the efficiency of a 100 kW microturbine were studied, and the heat exchanger with the highest efficiency was selected. The algorithm for designing and modeling the selected heat exchanger showed that the fin plate heat exchanger was the optimum choice for gas turbine power plants.

Tarrad et al. [58] used a simplified correlation to predict the air-side heat transfer coefficient of a plate-fin HE. The development of the formula was based on implementing the Buckingham-pi theorem. The physical dimensions of the HE’s plate, fin and tube geometry were introduced into the correlation to demonstrate their effects on the air-side heat transfer coefficient. The use is limited to air dry bulb and wet bulb temperatures in the range 16–40 °C and 13–20 °C, respectively, for flows through the tube banks. The study found that a crimped spiral fin had a higher heat transfer and pressure drop than the other types of fin. The total mean absolute errors of the predicted overall heat transfer coefficients and heat duty were 10% and 13%, respectively. The air-side performance of the heat exchanger with mixed fins was shown to give a better performance than the fin with delta-wing vortex generators, while the slit fin offered the best heat transfer performance at high Reynolds numbers.

Yang et al. [46] analyzed the heat transfer performance of plate-fin heat exchangers using well-validated CFD software, concentrating the selection of plate-fin surfaces on the estimation of the relative performance of an offset strip fin and a plain fin. The study indicated that it was not possible to attain a uniform heat transfer coefficient over all the surfaces in the fin channel, which is the basis of conventional concepts and which strongly restricts fin performance analysis. Using actual fin effectiveness, the authors measured fin performance. They confirmed the numerical results that a specific fin thickness-to-height ratio and fin density distribution combine to produce the best fin performance for a given mass flux. The authors noted that the optimal thickness-to-height ratio decreased with increasing Reynolds number.

Zhou and Yu [59] numerically investigated the heat transfer characteristics of a falling film type plate-fin heat exchanger as applied to cryogenic air separation units. The study presented and analyzed a mathematical model for a falling film plate-fin condenser/reboiler HE. The authors considered both laminar falling film evaporation and condensation in their model. They also considered the interaction between mass transfer and interfacial shear stress and how it related to the heat exchanger. The liquid film flow and heat transfer characteristics of oxygen and nitrogen fluids in the plate-fin HE was analyzed under given conditions by solving the relevant numerical model based on an iteration method. The authors considered different important factors such as liquid film thicknesses, local heat transfer coefficients of oxygen and nitrogen, the total local heat transfer coefficient, and the effects of inlet mass flow rate of oxygen liquid over the base plate to that over the fin surfaces on the wetted length of the heat transfer surfaces, on the heat transfer performance.

**Figure 27:**
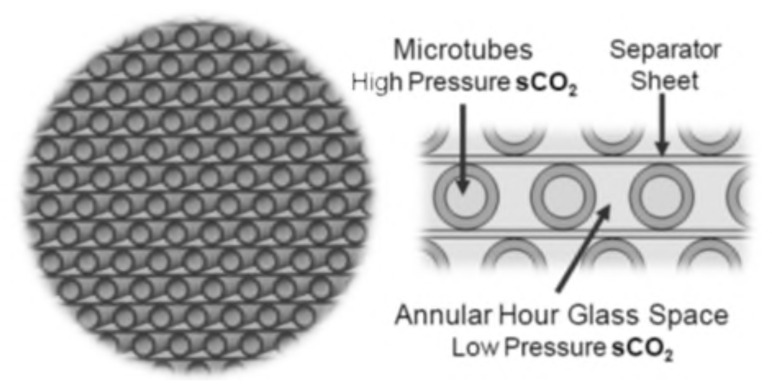
Recuperator tube bundle cross-section.

Chordia et al. [60] designed and tested a high-temperature plate-fin HE to be used as a recuperator for a supercritical carbon dioxide (sCO_2_) gas turbine power plant. The recuperator was required to operate at high temperatures with high-pressure differentials, 169 bar, between streams of sCO_2_. The plate-fin HE was designed to incorporate many features that optimized material use, improved reliability, and reduced cost. The air-to-sCO_2_ HE was a cross-flow, counter-current, and micro-tubular design (see Figure 27). Both the prototype recuperator and air-to-sCO_2_ HEs were characterized. Measured results for the recuperator confirmed the predictions of the heat transfer models.

**Figure 28:**
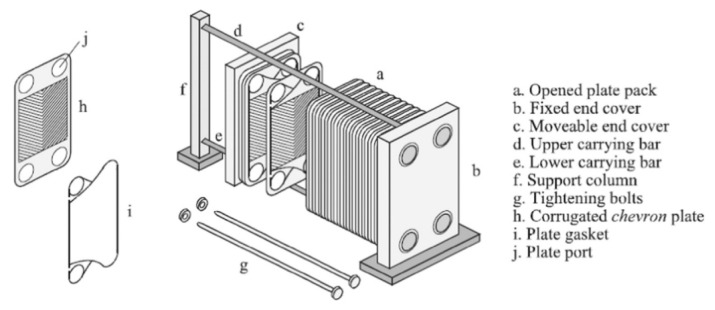
Typical structure of plate-fin heat exchanger with chevron plates [61].

Abou Elmaaty et al. [61] reviewed plate HEs used as recuperators with gas turbines. A good HE has a high heat transfer rate at low pumping power at minimum cost. Figure 28 shows a typical structure of a plate-fin HE with chevron plates. The paper discussed the integration of different considerations such as the structure of the plate HE, thermal performance, heat transfer enhancement, and the advantages and limitations of plate HEs. The authors concluded that the plate-fin HE worked efficiently in both single and two-phase flows, though two-phase flow still requires considerable further research. These authors also discussed the performance of corrugated plate HEs when using nano-fluids, see Section 5 below. Also, the paper summarized how this type of heat exchanger is widely used in many different engineering fields due to its simplicity in assembly/disassembly, ease of maintenance, and greater flexibility than other types of heat exchangers. Because its heat transfer area and cooling flow can be increased or decreased easily, it is commonly used for enlargement and upgrading. One important application is as an intercooler and/or recuperator in gas turbine systems.

A summary of what has been discussed in this section is presented in Table 4.

## 5. Introducing Nanofluids in Heat Exchangers

Most previous work has sought to improve the performance of heat exchangers by increasing effectiveness by adding fins and micro-fins, changing the shape and/or construction materials of the HE. However, a significant recent development is to use a nanofluid, as the working fluid to improve the performance of an HE [62,63,64,65,66,67,68,69]. The coolant properties are important factors affecting the overall performance of a HE, such as when it is used as an intercooler [70]. Nanofluids that consist of nanoparticles suspended in base fluids can give superior thermal conductivity and heat transfer performance compared with conventional coolants, such as water and ethylene glycol, which have lower thermal conductivities [71,72]. The increase in total heat flow rate in the presence of nanoparticle concentrations is explained as due to an increased collision rate between nanoparticles and the walls of HE channels.

**Figure 29:**
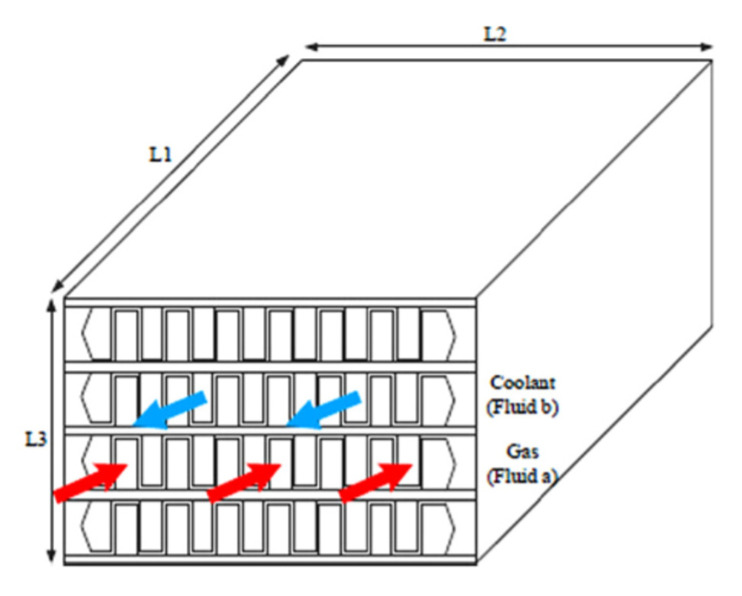
Schematic representation of reverse flow plate-fin [75].

**Figure 30:**
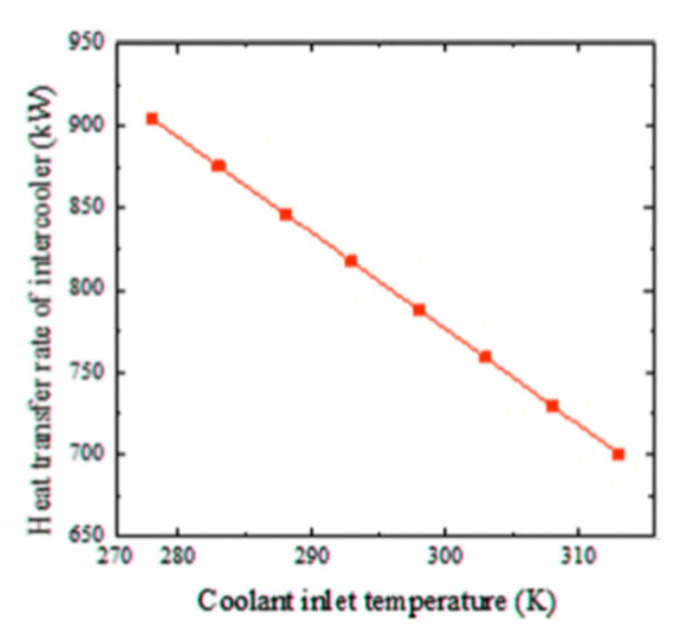
Heat transfer rate of intercooler vs. coolant inlet temperature.

Zhao et al. [73] investigated nanofluids as a coolant in the intercooler of a gas turbine Brayton cycle. Aluminum oxide (Al_2_O_3_) and copper (Cu) nanoparticles [74] in a water base were used in an intercooler in a marine gas turbine. The performance of the nanofluids was assessed using the effectiveness-number of transfer units method to evaluate flow characteristics and heat transfer performance of the intercooler. The thermophysical properties of nanofluids were taken from previously published studies. The study investigated the effects of nanoparticle volume concentration, flow Reynolds number, coolant inlet temperature, and gas side operating parameters on the intercooler’s flow and heat transfer performance. The flow patterns of the plate HE is shown in Figure 29. A sample set of the more important results is shown in Figure 30.

**Figure 31:**
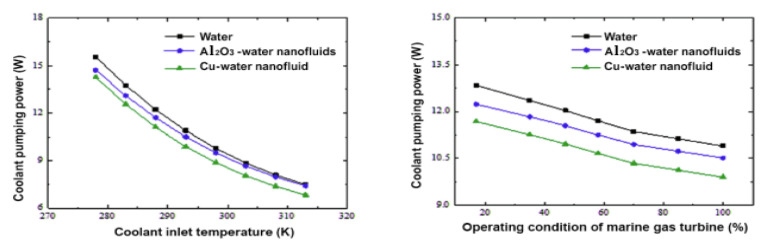
Coolant pumping power vs. coolant inlet temperature under part load.

Generally, the performance of nanofluids follows the same general rules as other coolants; see Figure 30, which shows that as the inlet temperature of the nanofluid is increased, the heat transfer is decreased due to reduced heat transfer between air and nanofluid. The results obtained by Zhao et al. [73] show that the nanofluids improved the heat transfer performance compared to water, as shown in Figure 31. Figure 31 also shows that Cu–water nanofluids required less pumping power under the same operating conditions than Al_2_O_3_–water nanofluids, which required less pumping power than water.

An important operating parameter is the concentration of nanoparticles in the base fluid and the proportion of base fluid in the water. The overall heat transfer performance of the intercooler can be increased by increasing the nanoparticle volume concentration and coolant Reynolds number.

**Figure 32:**
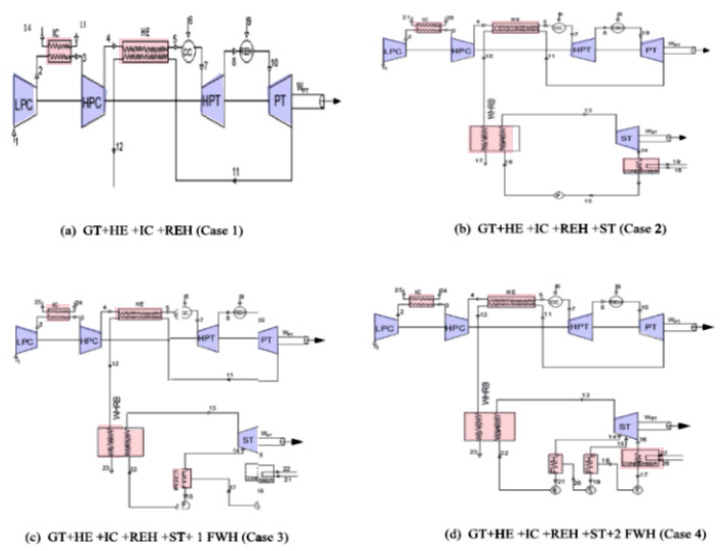
Schematic diagrams for Brayton gas turbine cycle in different modifications.

A similar investigation by Abam et al. [76] investigated experimentally the variation of intercooler effectiveness on exergo-economic and exergo-sustainability with different modifications of Brayton cycles used to generate electricity, see Figure 32. A component by component exergy-cost balance was established by the investigation.

The numerical analysis showed a steady improvement when proceeding from Case 2 to 3 to 4. Adding a feedwater heater (FWH) and intercooler to the modified Brayton cycles, especially in Cases 3 and 4, reduces the EFF by 3.7% for Case 3 and 4.9% for Case 4. Similarly, the exergy waste ratio was also lowest in Cases 3 and 4. The reduction in environmental impact was most significant in Case 4, as seen by the high exergy efficiency values. The findings indicate that the intercooler and other modifications such as the feedwater heaters had marginal effect. Cases 3 and 4 were the most sustainable with significant values of ESI at higher effectiveness.

As shown above, the heat transfer performance of HEs can be improved by using nanofluids as a coolant. Bahiraei et al. [77] reviewed the literature up to 2018 on the use of nanofluids on the heat exchangers and summarized investigations conducted on the use of nanofluids in heat exchangers such as plate, double-pipe, shell-and-tube, and compact heat exchangers and produced some fascinating insights into the use of nanofluids in heat exchangers and showed a rapid development in the characteristics of the nanofluids used. Kumar et al. [78] reviewed the application of nanofluids to plate heat exchangers to improve thermal performance by introducing new substances with enhanced properties as working fluids.

**Figure 33:**
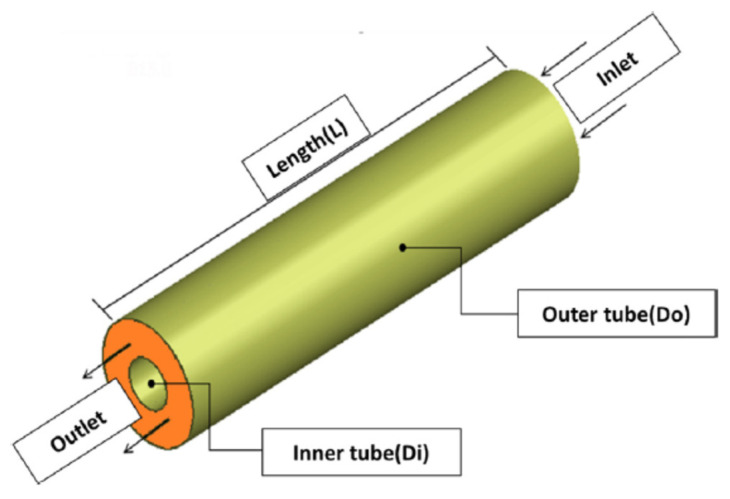
Geometric model of the concentric annular heat exchanger, [79].

Hosseini et al. [79] investigated turbulent heat transfer through a concentric annular heat exchanger, see Figure 33, containing a distilled water-based nanofluid with clove-treated multi-walled carbon nanotubes (C-MWCNTs). The authors tested the thermal and hydrodynamic properties via numerical simulation (finite volume method) for uniform and constant heat flux boundary conditions.

When preparing the homogeneous MWCNT nanofluid, the authors did not use the conventional corrosive, hazardous acid-based functionalization methods but treated the MWCNTs with clove buds in a single pot using an environmentally friendly free radical grafting reaction. The investigators analyzed the C-MWCNTs using Fourier transform infrared spectroscopy. Then they performed numerical simulations to predict the nanofluids’ convective heat transfer and hydrodynamic performance considering three concentrations of C-MWCNTs: 0.075, 0.125, and 0.175 wt.%. The effective thermo-physical properties of the nanofluids were obtained experimentally and used when solving the governing three-dimensional fluid dynamic equations using the k-ϖ turbulence model. The result showed good agreement between simulated and experimental results. Also, the results indicated that the addition of a small fraction of C-MWCNTs into the distilled water significantly enhanced the convective heat transfer coefficient relative to distilled water. On the other hand, the authors indicated that the friction factor did not vary considerably for the nanofluids over the concentration range used. In general, it was concluded that the simulation model could be used to successfully predict the convective heat transfer and hydrodynamic characteristics of C-MWCNT/distilled water nanofluids in a concentric annular heat exchanger.

**Figure 34:**
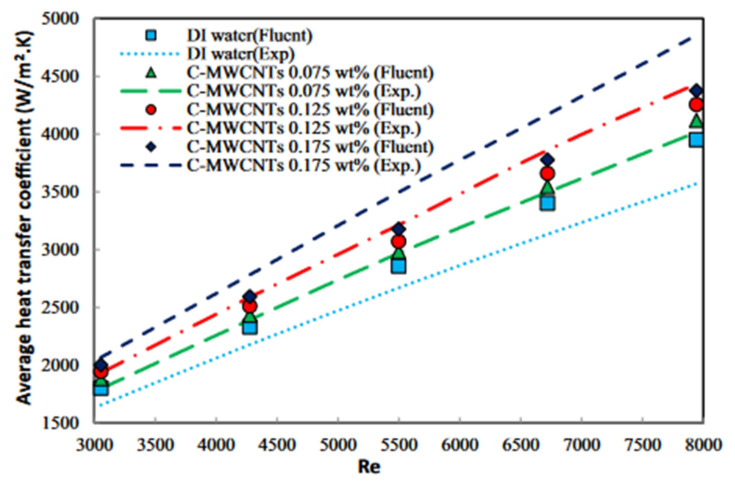
Variation of the average heat transfer coefficient with respect to the Reynolds number for C-MWCNT/distilled water nanofluids in a concentric annular heat exchanger.

As shown in Figure 34, the effect of varying the Reynolds number on the average heat transfer coefficients for C-MWCNT/distilled water nanofluids with different nanoparticle concentrations. The results were obtained with constant wall heat flux boundary conditions. As expected, the average heat transfer coefficient increased as the Reynolds number increased with average heat transfer coefficients greater for higher concentrations of C-MWCNTs.

The results showed that the pressure drop, and friction factor were slightly higher for the C-MWCNT/distilled water nanofluids than distilled water. At a C-MWCNT concentration of 0.175 wt.%, the friction factor and pressure drop were increased by 0.028 and 20.41%, respectively. The small increase in friction factor was attributed to the slightly higher viscosity of C-MWCNT nanofluids than distilled water.

The use of internal fins in HE tubes is an accepted practice for improving heat transfer. Baba et al. [80] reported an experimental study of forced convective heat transfer in a double tube counter flow HE using multiple internal longitudinal fins and Fe_3_O_4_–water nanofluid as the working medium. The convective heat transfer coefficients and pressure drop were investigated for the nanofluid flowing in a horizontal circular tube with internal longitudinal fins under turbulent flow conditions (5300 < Re < 49,200) and with the volumetric concentration of Fe_3_O_4_ nanoparticles in the range 0 < φ < 0.4%. The results showed that the heat transfer rate increased by 80–90% for the same operating conditions when the finned tube replaced the non-finned tube in the HE. The Nusselt number for the Fe_3_O_4_–water nanofluid increased as the Reynolds number increased, as did the friction factor, but the pressure drop was higher in the finned tube HE than the non-finned tube HE due to the presence of the fins, which introduced resistance to the flow.

Yahiaoui et al. [81] have recently argued that the convective heat transfer can be improved by adding particles to the base working fluid, essentially using a nanofluid. Abu-Nada et al. [82] added that there had been significant research on the use of nanofluids in thermal systems over the past two decades. Nanofluids can enhance the heat transfer rate due to their superior thermal properties, positively impacting heat transfer when suitable nanoparticles are suspended in water or other liquid. This process can enhance heat transfer in forced convection applications, but the effect depends on the volume fraction of the nanoparticles. Enhanced heat transfer is possible because nanofluids typically have higher thermal conductivity than conventional liquids, enhancing heat transfer rates in engineering systems such as HEs.

Kumar et al. [83] investigated the effect of chevron angle on heat transfer performance of a plate heat exchanger using a ZnO/water nanofluid. The experimental investigation focused on the impact of change in the symmetric angles (β). The tests used chevron angles 30°/30° and 60°/60° and mixed 30°/60° on the plate HE. The particle volume concentration of the ZnO/water nanofluid being used as coolant was in the range 0.5–2.0%. The experiments investigated the effect of the different chevron angles on performance factors, including heat transfer rate ratio, heat transfer coefficient ratio, overall heat transfer coefficient ratio, pumping power ratio, exergy loss, exergetic efficiency, and total entropy generation. The experimental results showed that there was an optimum enhancement in heat transfer rate ratio, heat transfer coefficient ratio, and optimum reduction in exergy loss obtained at angles of 60°/60° for 1.0% particle volume concentration of ZnO/water nanofluid.

**Figure 35:**
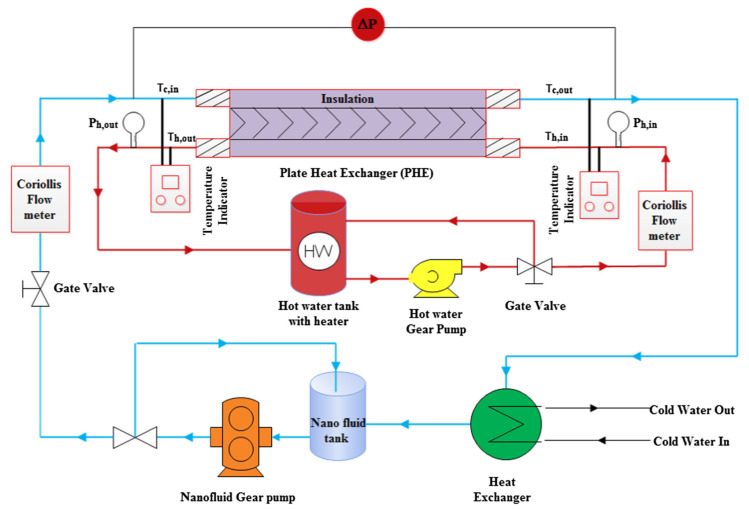
Schematic of experimental apparatus used by Kumar et al. [83].

The experimental test rig used by Kumar et al. [83] consisted of two loops: cold and hot fluid. The cold fluid loop consisted of four components: nanofluid tank (25 L), gear pump, gate valve, and Coriolis flowmeter, see Figure 35. A 4 kW heater was used in the hot loop to heat the nanofluid, followed by a hot fluid pump, a gate valve, and a second Coriolis flowmeter. Water was the working fluid in this loop. First, a nanofluid solution of the requisite concentration was prepared, and the nanofluid tank filled with it. The volume flow rate of nanofluid was varied from 0.5 to 2.0 lpm, while the volume flow rate of pure hot water was kept constant at 3 lpm.

Heris et al. [84] studied the heat transfer performance of CuO-water nanofluids and found that the nanofluid enhanced heat transfer performance compared to water. In a later study, they examined the heat transfer performance of laminar flow forced convection for Al_2_O_3_/water nanofluid within a circular tube with constant wall temperature. The study again found that the presence of nanoparticles enhanced the heat transfer process, with the heat transfer coefficient increasing with increased concentration of nanoparticles in the nanofluid.

**Figure 36:**
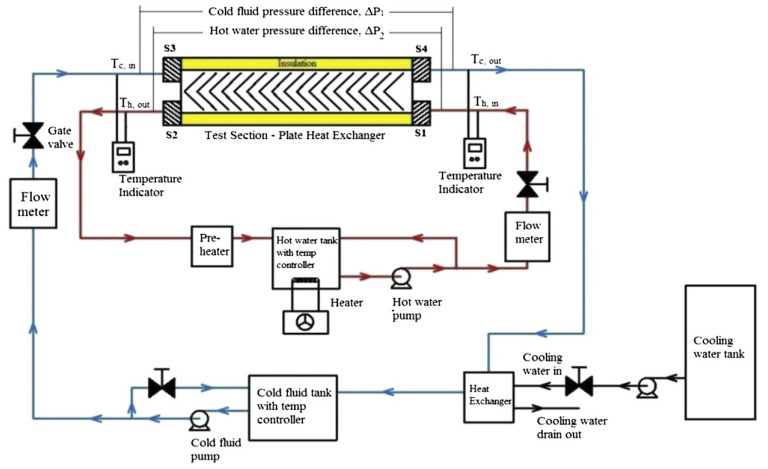
Schematic of the experimental test rig used by Tiwari et al. [85].

**Figure 37:**
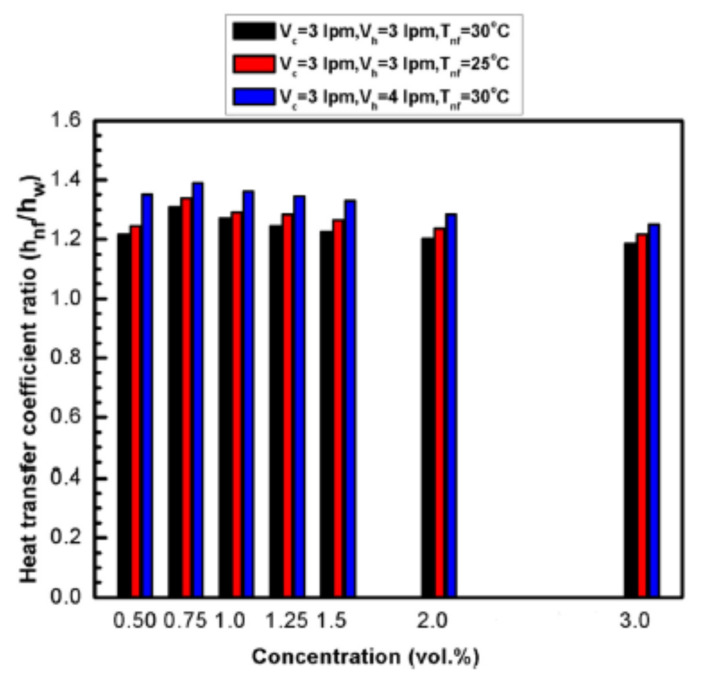
Heat transfer coefficient ratio vs. CeO_2_/water nanofluid concentration [85].

Tiwari et al. [85] investigated both numerically and experimentally the heat transfer performance and pressure drop when using CeO_2_/water nanofluid in a chevron-type corrugated plate heat exchanger, see Figure 36. A wide range of CeO_2_/water nanofluid concentrations were used (0.5, 0.75, 1.0, 1.25, 1.5, 2.0, and 3% by volume) to discover the optimum concentration for CeO_2_/water nanofluid. The study also used a range of fluid flow rates (1.0, 2.0, 3.0, 3.5, and 4.0 lpm). Different CeO_2_/water nanofluid operating temperatures were used. The maximum heat transfer improvement over base CeO_2_/water nanofluid was determined. The investigators determined the thermophysical properties of the nanofluid in a separate series of experiments. Figure 37 shows that using the nanofluid at an optimum concentration of 0.75 vol.%. there was an increase of 39% in the heat transfer coefficient compared to water, As expected, the heat transfer coefficient of the nanofluid increased both with an increase in the volume flow rate of the hot water and nanofluid, and with decrease in the temperature of the nanofluid. The pressure drop with the nanofluid was approximately the same as for water alone up to optimum volume concentration, suggesting that such nanofluids may be extremely useful in practical applications.

**Figure 38:**
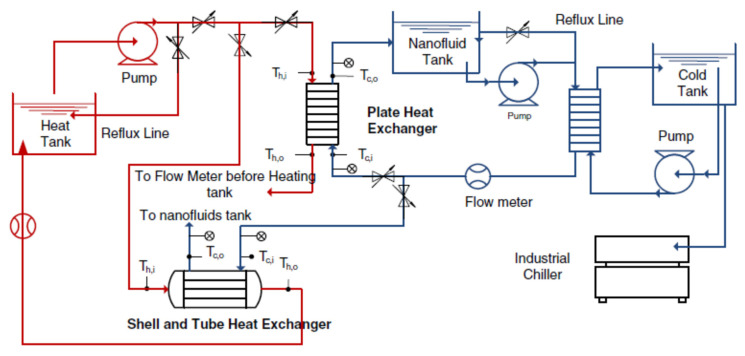
Schematic of test rig used to determine heat transfer coefficient and pressure drop in the study of Anoop et al. [86].

Anoop et al. [86] also evaluated nanofluids as the working medium in heat exchangers. This study investigated the thermo-physical properties and thermal performance of nanofluids in plate-fin and shell-and-tube HEs. Different volumetric concentrations of 2%, 4%, and 6% of silicon dioxide–water (SiO_2_–water) with nanoparticles of diameter 20 nm were in a base fluid of pure water. The overall heat transfer coefficient and pressure drop of water vs. nanofluid concentration in laboratory-scale plate-fin and shell-and-tube heat exchangers were assessed experimentally. The test rig used to measure the heat transfer characteristics of the nanofluid is shown in Figure 38. Hot and cold flow loops were set up to operate with counterflow laboratory-scale plate-fin and a shell-and-tube HEs. The plate-fin HE consisted of a corrugated stainless steel plate (70 × 200 mm) embossed with a herringbone pattern. There was a 2 mm gap between each plate. The shell-and-tube HE consisted of 28 single-pass tubes with an inner diameter of 5 mm. The nanofluids flowed through these tubes as the cold medium. Hot water was circulated through the shell side with a diameter of 50 mm. The study also considered fouling on the heat exchangers’ contact surfaces.

The results showed that heat transfer coefficients for nanofluids depended on the flow rate through the heat exchangers and nanofluid concentration. Anoop et al. [84] also measured the pressure drop across with a heat exchanger and showed that replacing water as the working fluid with the nanofluid increased the pressure drop, which is considered a major disadvantage to using nanofluids in industrial applications.

**Figure 39:**
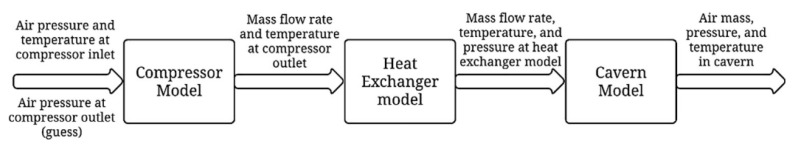
Flowchart with input and output data, [87].

Malekan et al. [87] investigated the effect of applying a magnetic field on the heat transfer properties of a magnetic nanofluid in a small-scale double pipe heat exchanger. The study used Fe_3_O_4_/water nanofluid as the secondary fluid in the specially designed double pipe heat exchanger. Figure 39 shows the flow chart for input and output parameters. The results showed that the cavern temperature decreased by increasing the nanofluid’s mass flow rate. The convective heat transfer coefficient of the nanofluid increased when the volume fraction of the nanoparticle increased, enhanced by turbulent flow induced by the magnetic field. However, increasing either the volume fraction or magnetic field increased the pressure drop and friction factor on the nanofluids side.

An experimental investigation by Maddah et al. [88] investigated the thermal performance of a double pipe heat exchanger using Al_2_O_3_-TiO_2_ nanofluids as a working medium and found that using the nanofluids significantly enhanced the performance of the heat exchanger. The heat exchanger was loaded with an Al_2_O_3_-TiO_2_ hybrid nanofluid under turbulent flow conditions. The authors used an exergy approach to study and evaluate the system and experimental data to verify the exergy analysis of the nanofluid-loaded double pipe heat exchanger.

The test variables were: nanofluid concentration in the range of 0.2 to 1.5 vol.%, Reynolds number from 3000 to 12,000, and twist ratio between 2 to 8. Statistical analyses of the experimental results confirmed that using the Al_2_O_3_-TiO_2_ hybrid nanofluid rather than conventional water as the heat transfer fluid in the HE improved exergy efficiency. As expected, increasing the nanoparticles volume concentration and the Reynolds number improved the thermal performance of the HE, which also improved the exergy efficiency.

Nakhchi and Esfahani [89] presented a numerical analysis of the heat transfer for Cu-water nanofluid as the working medium flowing through a circular duct, which was fitted internally with cross-cut twisted tape. They applied the k−ϵ turbulence model in three dimensions to simulate the plain tube heat exchanger. Reynolds numbers were in the range 5000 to 15,000 and volume fraction of nanoparticles was from 0 to 1.5 vol.%. The numerical simulation indicated that using nanofluids with higher Re numbers increased the heat transfer rate between the two sides of the HE due to increased convective heat transfer and thermal conductivity. In addition, a higher Re value was better for maintaining the homogeneity and stability of the nanofluid. The results showed that the heat transfer coefficient increased by up to 23.20% for a nanoparticle volume fraction of 1.5%. Finally, it was shown that the thermal performance increased with increase in the volume fraction of nanoparticles.

Bahiraei et al. [90] investigated the effect of employing a new environmentally friendly biological nanofluid containing functionalized graphene nanoplatelets on the thermal and hydrodynamic characteristics of a countercurrent spiral heat exchanger. The cold water flowed on one side of the heat exchanger, while the hot nanofluid or hot base fluid flowed on the other. The results showed that the heat transfer rate, overall heat transfer coefficient, and performance index (ratio of heat transfer rate to pressure drop) increased as either Reynolds number or nanoplatelet concentration increased. The performance index for the nanofluid increased over 140% when increasing the Reynolds number from 1000 to 3000. On the other hand, while the effectiveness and number of transfer units decreased with increasing Reynolds number, the effectiveness of the HE was greater than 0.85 in all cases investigated. Additionally, the nanofluid was doubly efficient at higher values of Reynolds numbers due to a decrease in fouling. However, the pressure drop across the heat exchanger was higher when using the nanofluid due to its greater viscosity. This was especially noticeable at high Re values and when the nanofluid was cold.

Ionescu and Neagu [91] numerically studied the effect of using an Al_2_O_3_–water nanofluid on the performance of a cross-flow micro heat exchanger. The study numerically modeled the physical properties of water and the nanofluid, such as density, viscosity, thermal conductivity, and specific heat capacity using Finite Element Method-based Comsol Multiphysics software. The system to be modeled was a cross-flow micro heat exchanger manufactured from 50 stainless steel plates. Each of these plates had 34 rectangular cross-section microchannels of depth 100 μm and width of 200 μm. The nanofluids had volumetric concentrations of Al_2_O_3_ of 0.5%, 1%, and 1.5%, and its temperature was in the range of 293–333 K. The numerical analysis of the cross-flow micro HE, confirmed by experiment, showed that total heat flow rate, pressure drop, and friction factor increased with increase in nanoparticle volume concentration and thus the pumping power required to drive flow through the HE channels was increased. Compared to pure water, a volumetric concentration of 1.5% Al_2_O_3_ increased the Fanning friction factor by nearly 2% for a 1 L/min flow rate. At the maximum flow rate of 1 L/min, there was an enhancement of 2.1% in the total heat transfer rate for a nanofluid with a volume concentration of 1.5%.

**Figure 40:**
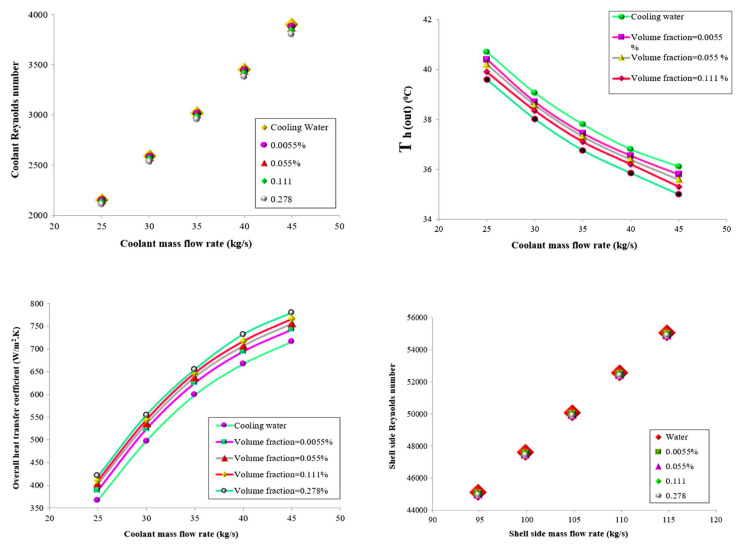
Performance of HE with CNT-water nanofluids.

Hosseini et al. [92] studied and investigated the performance of a CNT-water nanofluid in low volume concentrations (0.0055%, 0.055%, 0.111%, and 0.278%) as the coolant in a shell-and-tube intercooler in an LPG absorber tower, see Figure 40. The authors simulated the HE using ASPEN HTFS+ 7.3 software. They determined the heat exchanger thermal performance and analyzed and compared the results with cooling water. The results indicated that increasing the nanofluid and the hot fluid mass flow rates increased the heat transfer rate. With CNT volume fraction of 0.278%, the overall heat transfer coefficient and heat transfer rate increased by about 14.5% and 10.3%, respectively, compared to pure water. This result led to a reduction in the heat transfer area of the HE, which was considered a positive advantage due to consequential reductions in heat loss, pumping power, weight, and cost. However, the pressure drop increased with increasing CNT concentration due to increased viscosity.

Albadr et al. [93] also investigated heat transfer through a heat exchanger using Al_2_O_3_ nanofluid at different concentrations in the range (0.3–2.0 vol.%). This was an experimental study on forced convective heat transfer. The investigators conducted experimental work on a horizontal shell-and-tube HE under turbulent and counter flow conditions. The Al_2_O_3_ nanoparticles used in the study had a mean diameter of 30 nm. The results showed that the convective heat transfer coefficient of the nanofluid was slightly higher than that of the base fluid at the same mass flow rate and inlet temperature. The heat transfer coefficient of the nanofluid increased with an increase in both mass flow rate and volume concentration of nanoparticles in the Al_2_O_3_ nanofluid. However, increasing the volume concentration increased the friction factor and hence increased the pressure drop due to an increase in the viscosity of the nanofluid.

The main aim of using nanofluids as a working medium in HEs is to reduce energy dependence. Using nanofluids in the industry should have an immediate significant impact on current energy usage and the environment and sustainable development.

A nanofluid of aluminum oxide and copper oxide in ethylene glycol base fluid with a volumetric concentration in the range of 2% to 50% was used by Zamzamian et al. [94] to study the effect on forced convective heat transfer coefficient in plate-fin and double pipe counter flow HEs for turbulent flow. The inner diameter of the inner pipe of the double pipe HE was 12 mm and was manufactured from copper with a thickness of 1 mm. The total length of the heat exchanger was 70 cm. The outer shell was prepared from green pipes, with internal diameter of 50.8 mm. The plate heat exchanger was modeled on a common domestic radiator, 40 cm in height, 60 cm in length, with small rectangular fins. The theoretical calculations and measured data showed a significant increase in convective heat transfer coefficient when using nanofluids compared to the base fluid. As the nanoparticles concentration increased, so did the convective heat transfer coefficient.

Rao et al. [95] experimentally investigated the heat transfer rate of nanofluids in a shell-and-tube HE applied to a gas turbine power plant. They considered both single and multi-tubes under turbulent flow conditions with forced convection. In this study, alumina nanoparticles were prepared using the Sol-Gel method. Alumina nanoparticles of mean diameter 22 nm were mixed in different volumetric concentrations (0.13%, 0.27%, 0.4%, and 0.53%) with water as a base fluid using an ultra-sonicator. The properties of the nanofluids were calculated. The effects of inlet temperature and mass flow rate of the nanofluid on the heat transfer performance of the HE were investigated. The result showed that the heat transfer rate increased with decreasing particle size in the nanofluid. The authors also demonstrated that the heat transfer rate improved compared to conventional fluids.

**Figure 41:**
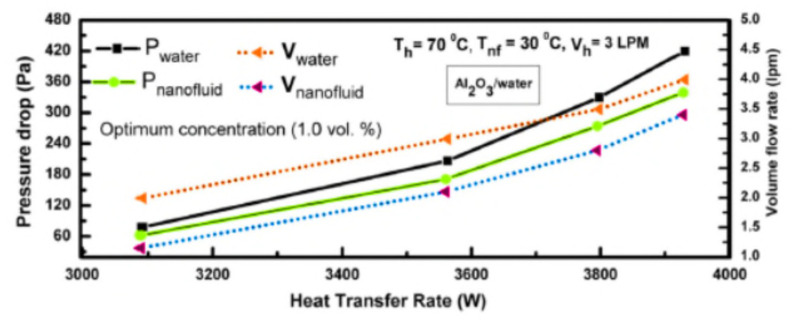
Experimental measurements of pressure drop across plate HEs with nanofluids as coolants.

Recently, the miniaturization of plate HEs and increasing energy efficiency have become focal points for research and development. Figure 41 shows the heat transfer rate vs. pressure drop based on different operating conditions of nanofluids from a practical study of plate HEs by Tiwari et al. [96].

Most published reports on the use of nanofluids in HEs concluded that nanofluids improved the heat transfer rate sufficiently for them to be an essential development. Increasing the nanofluid concentration in the working medium improved the heat transfer rate and effectiveness of the HE, but at the cost of increasing the viscosity of the working fluid, which led to an increase in the friction factor and increased pressure drop. It follows that the nanofluid concentration should be optimized and controlled, relatively high concentration should be used with higher values of Reynolds number. Using nanofluid technology should enable a decrease in the mass and volume of HEs, with consequent improvement in the energy loss factor.

Table 5 summarized the reviewed literature on the effects of nanofluids on heat transfer performance of various HEs.

## 6. Thermophysical Properties of Nanofluids: Fundamentals and Characteristics

Maxwell’s theory was the first theory to attempt to explain why the thermal properties such as heat transfer rate of the working fluid could be improved by adding millimeter or micrometer-sized particles [96]. This could produce important advantages such as reduced size and cost of HEs. However, one disadvantage related to the use of such particles is their settling, which, for larger particles, can be relatively rapid and may result in complete separation of the two phases, causing a decrease in the thermal conductivity and clogging due to sedimentation. Another problem that can occur from using nanofluids containing larger particles is the need to increase pump pressure to maintain the flow of the working fluid [97].

Recently there has been considerable research into using nanofluids in heat transfer devices, where the nanofluids are produced using nanoparticles with a size below 100 nm in a base fluid [97]. Generally, nanoparticles such as CNTs, Fe_2_O_3_, Al_2_O_3_ can be added to different base fluids such as water, oil, and glycol [98]. Han [99] carried out a numerical review of many of the nanofluids used in 2008 to improve the thermal properties of heat transfer devices. Han measured the thermal conductivities of some of the most common materials used as nanoparticles. The results are shown in Table 6.

Surfactants are used to stabilize the nanoparticles in the base fluid to counteract the natural tendency of the nanoparticles to aggregate [100]. The surfactants are absorbed on the surfaces of the nanoparticles so that the surfactant molecules create a barrier preventing agglomeration and particle solubility, thus guaranteeing long-term stability [99]. However, surfactant action at high temperatures remains a big concern.

Eastman [98] measured the thermal properties of different nanofluids. He concluded that the thermal conductivity of water was increased by 20% by adding 5 vol.% CuO nanoparticles. However, the heat transfer coefficient of water under dynamic flow conditions increased more than 15% with less than 1 vol.% CuO particles.

Xuan and Roetzel [101] investigated the enhanced heat transfer mechanisms obtained through the use nanoparticles materials. They used two different approaches for deriving heat transfer properties of the nanofluid, taking into account the effect of transport properties and thermal dispersion. The results showed that convective heat transfer is increased by using a nanofluid rather than a pure material due to increased thermal conductivity and Prantl number. A similar paper by Pozhar [102] was based on the estimated viscosity of nanofluids obtained using a simulator. He used a simplified version of the Pozhar-Gubbins expression to calculate the viscosity. The success of the Pozhar-Gubbins when predicting nanofluid viscosity is because the theoretical expression accounts accurately for the nanofluid structure. However, using Pozhar-Gubbins to calculate viscosity gives a high error with non-homogeneous nanofluids when the transportation factor is high.

**Figure 42:**
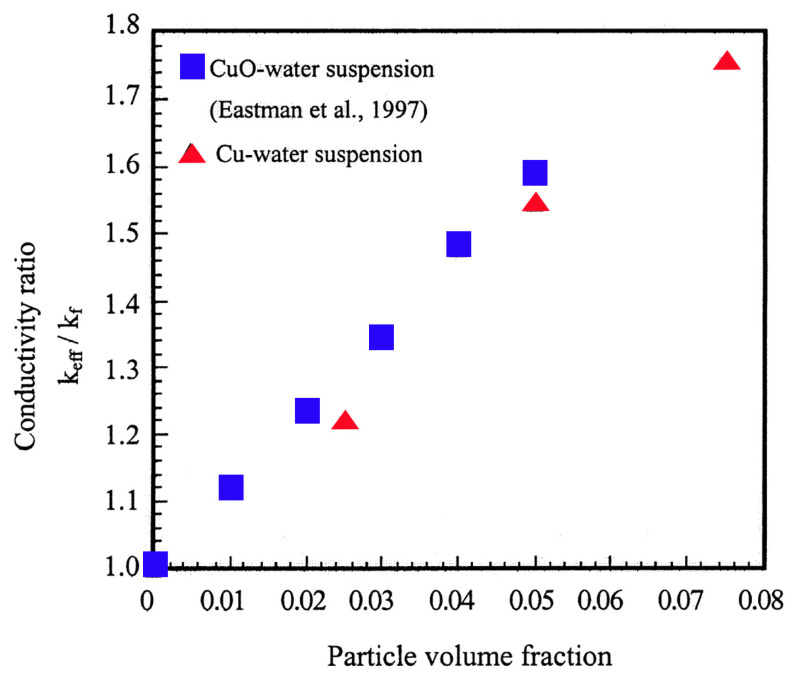
Effect of particle volume fraction on the thermal conductivity of a nanofluid.

Preparation of nanophase particles (average diameter < 100 nm) is the first and critical step in preparing the nanofluid to improve conventional fluids’ heat transfer performance. Xuan and Li [103] presented a procedure for preparing a nanofluid based on a suspension consisting of nanophase powders and a base liquid. Different samples of nanofluids were prepared. Numerous photographs were taken for analysis and assessment of the stability and evenness of the suspension. A hot-wire apparatus was used to measure the thermal conductivity of nanofluids with suspended copper nanophase powders. Factors such as the volume fraction, the dimensions, shapes, and properties of the nanoparticles were discussed. The measured results showed that an increase in particle volume fraction of nanoparticles increased the thermal conductivity directly due to the increase in the fluid’s viscosity as a result of the decrease in distance between the particles. Figure 42 shows the outcome of their results.

An investigation by Özerinç [104] of the heat transfer performance of nanofluids concluded that classical heat transfer concepts were not valid for the analysis of nanofluids, and the mechanisms that enhanced the thermal conductivity of nanofluids were not yet fully understood. The results obtained by Özerinç showed that the thermal conductivity of nanofluids is a function of several parameters including particle volume fraction, particle size, and fluid temperature. Özerinç indicated that the heat transfer improvement due to using nanofluids was due to an increase in the thermal conductivity due to an increase in kinematic viscosity, which leads to an increase in thermal dispersion due to the random motion of the nanoparticles.

A model developed by Khanafer et al. [105] to analyze the heat transfer performance of nanofluids inside an enclosure takes into account the solid particles’ dispersion. The transport equations were solved numerically using the alternating direct implicit procedure with the finite-volume approach. The results showed that the variances within different models were due to the problems of sediment and stability. The results also showed that the presence of suspended nanoparticles increased the heat transfer rate at any given Grashof number, and the nanofluid heat transfer rate increased with an increase in the nanoparticle volume fraction. A similar numerical study by Bhattacharya [106] presented an estimate of the effective thermal conductivity of different nanofluids. The model simulated Brownian dynamics and appeared to have been very effective compared to other similar methods because the values obtained were very accurate.

The thermal conductivity of nanofluids produced by adding single and hybrid nanoparticles to water, including CNTs, copper nanoparticles (CuNPs) and gold nanoparticles (AuNPs), and hybrids such as CNT–CuNP or CNT–AuNP were investigated experimentally by Jana et al. [107]. The results showed that mono-type nanoparticle suspensions, e.g., CuNPs, produced the greatest improvement in the thermal conductivity. The stability of the nanofluids was estimated by UV–vis–NIR spectrophotometer, and it was observed that the stability was influenced by characteristics of the nanoparticles, such as size and shape [108].

Beck et al. [109] investigated the effect of nanoparticle size on the thermal conductivity of alumina nanofluids experimentally. They measured the thermal conductivity for different diameter particles in the range 8–282 nm suspended in water or ethylene glycol. The results showed that, for a given temperature, the thermal conductivity enhancement by these nanofluids increased as the particle size increased. The thermal conductivity was higher than glycol for water as a base fluid. Another study by Longo et al. [110] for measuring the thermal conductivity and the dynamic viscosity of an Al_2_O_3_–water nanofluid (with particle fraction 1–4 vol.%) below the ice point (0 °C), and TiO_2_–water nanofluid (with particle fraction 1–6 vol.%) in the temperature range (1–40 °C). Both sets of measurements were made at atmospheric pressure.

A series of experiments were carried out to measure thermal conductivity and rheological properties of graphite/oil nanofluids by Wang et al. [111]. A Scanning Electron Microscope and Transmission Electron Microscope were used to observe particle size and morphology of a graphite nanofluid. The thermal conductivity of the graphite/oil nanofluids was measured using a transient hot-wire method. It was found that parameters such as milling time, dispersant concentration, graphite concentration, temperature, and standing time significantly affected the thermal conductivity. Moreover, the rheological properties were examined to reveal the microstructure lay in the nanofluids, which could supply a direct explanation of the enhancement of the thermal conductivity of the nanofluids. The results showed that the enhanced thermal conductivity of the nanofluids depended strongly on the volume fraction (vol.%) of graphite and increased nonlinearly with increasing loading but had a weak relationship with the temperature of the nanofluid. Promisingly, there when the graphite was increased from 0.68 to 1.36 vol.%. was an increase in thermal conductivity from 11% to 36%.

Sundar et al. [112] experimentally investigated the properties of hybrid composites of diamond and nickel (ND-Ni) nanoparticles. They prepared the nanoparticles by dispersing caboxylated nanodiamond nanoparticles in ethylene glycol and then mixing in nickel chloride and, at a reaction temperature of 140 °C, using sodium borohydrate as the reducing agent to produce the ND-Ni nanoparticles. Subsequent measurements showed that the degree of thermal conductivity enhancement increased as the wt.% of particles in ethylene glycol increased. The viscosity of the nanofluid also increased as the wt.% of nanoparticles increased and as the temperature decreased. With a 3.03 wt.% of ND-Ni nanoparticles dispersed in water and ethylene glycol, the nanofluid produced an increase in thermal conductivity of 21% and viscosity by 13%.

A simultaneaous study by Cieśliński et al. [113] measured the contact angle of sessile droplets for water–Al_2_O_3_, water–TiO_2,_ and water–Cu nanofluids. When preparing the nanofluid, they used ultrasonic vibration for 30–60 min to successfully disperse the nanoparticles at a concentration of 0.01, 0.10, and 1.00 wt.%. The contact angle was established directly using a KR-SS DSA10 goniometer or building digital images of droplets and using a geometrical method. The results showed that the droplet contact angle of the nanofluids depends on surface roughness, type of substrate, and material and concentration of the nanoparticles. There have been many previous experimental studies concerned with measuring the physical properties of nanofluids and these have demonstrated many factors can affect the results, including temperature, homogeneity, pressure, and stability. Thus, Loya et al. [114] used a new approach, Molecular Dynamics, to simulate the behavior of nanoparticles dispersed in a base fluid.

**Figure 43:**
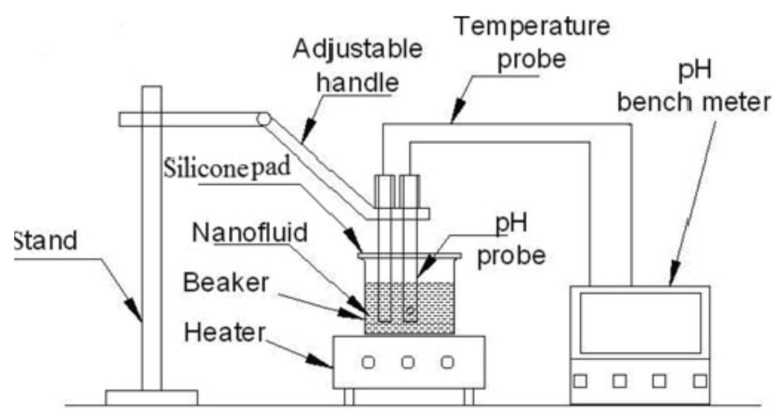
Experimental apparatus for measuring the pH of nanofluids [115].

The pH of a nanofluid can be a significant factor when determining fluid friction, viscosity, and rheological behavior. The test rig built by Konakanchi et al. [115] to measure the pH of three nanofluids is shown in Figure 43. In this study, the pH levels of aluminum oxide (Al_2_O_3_), silicon dioxide (SiO_2_), and zinc oxide (ZnO) nanoparticles dispersed in a mixture of propylene glycol and water were measured in the temperature range of 0 °C to 90 °C. The average particle diameters were between 10 nm and 70 nm. From these results, they derived new correlations for estimating the pH value.

**Figure 44:**
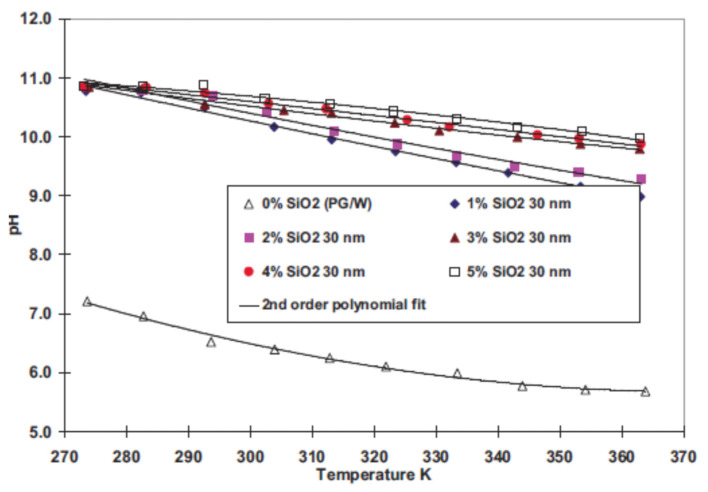
pH as a function of temperature for SiO_2_ nanoparticles in PG/W (60:40) base fluid for particle concentration ranging from 0 to 5.0 vol.% [115].

Figure 44 shows a sample of the experimental results obtained. With increase in temperature of the nanofluid, the pH decreased. The vol.% of nanoparticles in these nanofluids ranged from 0 to 10%. The experimental data showed that the contact angles of the nanofluids depended on their pH value. Also, knowing the pH accurately is important to prevent chemical corrosion of the materials.

Following the validation of the method, the pH values of different nanofluids were measured. These showed that the pH of nanofluids decreased with an increase in temperature and increased with an increase in volumetric particle concentration. For the same nanofluid at a fixed volumetric concentration, the pH was higher for larger particles. From the experimental data, empirical models were developed for the three nanofluids to express the pH as a function of temperature, volumetric concentration, and the size of the nanoparticles.

Buonomo et al. [116] investigated the effect of temperature and sonication time on nanofluid thermal conductivity for cases where the ultra-sonication technique was employed to prepare mixtures of different concentrations of Al_2_O_3_ with water as the base fluid. The measurements were carried out using the nano-flash method, which the authors claimed gave short measurement times, easy sample preparation, and high accuracy. The results showed that the stability and thermal conductivity enhancements obtained with Al_2_O_3_-water nanofluids depended on sonication time and the energy supplied to the fluid during its preparation.

Parametthanuwat et al. [117] experimentally investigated the thermal properties and behavior of 0.5 wt.% silver in a silver nanofluid. The nanoparticle-based nanofluids contained oleic acid and potassium oleate surfactant (OAK^+^) with concentrations of 0.5, 1.0, and 1.5 wt.%. The experiments were conducted at temperatures from 20°C to 80 °C. It was shown that the thermal properties of a nanofluid with 1.0 wt.% OAK^+^ were improved by about 28% at 80 °C compared to deionized water. In general, the results showed that the thermal performance when using nanofluid was improved over that of the base fluid by 80% approximately. The rheological data showed that the viscosity of the nanofluid depended significantly on temperature. As the shear rate increased, the shear stress of the nanofluid increased; however, the viscosity of the nanofluids first decreased and then stabilized.

**Figure 45:**
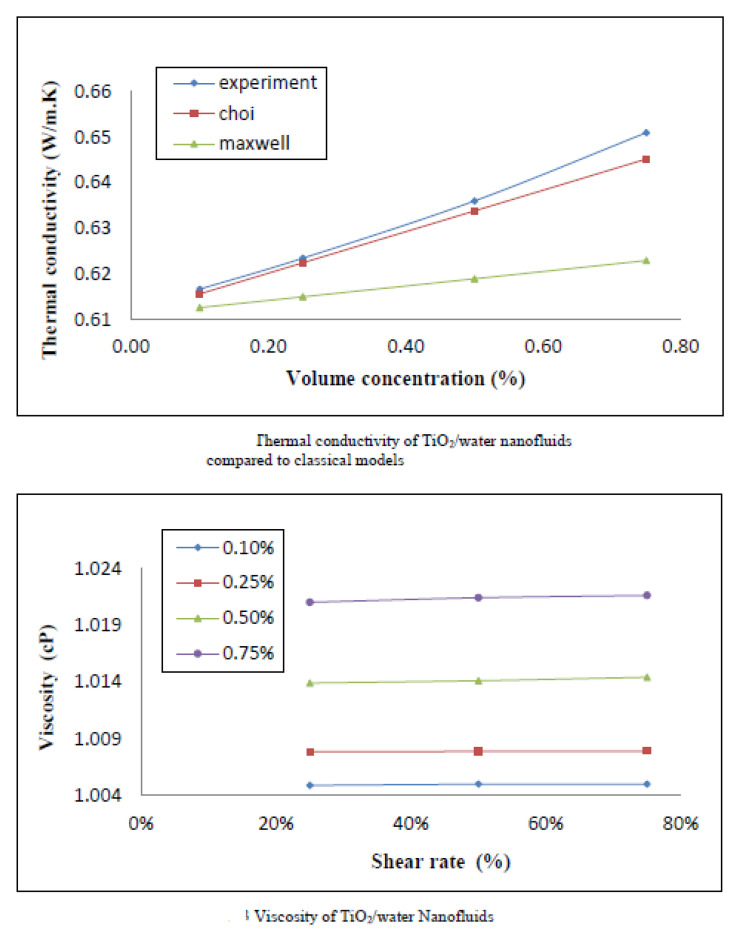
Thermal conductivity of TiO_2_/water nanofluid as a function of vol.% of nanoparticles, and viscosity as a function of shear rate% [118].

The heat transfer behavior of TiO_2_-water nanofluids with volume concentrations of 0.10, 0.25, 0.50, and 0.75 vol.% was investigated by Arulprakasajothi et al. [118]. The nanofluids were prepared using the two-step method through a tube heat exchanger. Stability, thermal conductivity, and viscosity were measured. Figure 45 presents the results obtained, showing the effect of volume concentration on thermal conductivity and viscosity. As expected, an increase in the concentration of nanoparticles increased the thermal conductivity and the convective heat transfer coefficient due to an increase in the Nusselt number. The experimental results showed that the Nusselt number increased with increase of particle volume fraction. The Nusselt number enhancement reached 13.2% over the base fluid for 0.75 wt.%. Figure 45 also shows the obtained relation between shear stress and viscosity. As nanoparticle concentration increased, the viscosity increased, indicating that the shear force increased.

Yerrennagoudaru et al. [119] carried out a series of CFD simulations and experiments that showed the thermal properties, especially thermal conductivity, of heat transfer mediums, were improved by using nanoparticles with diameters in the range of 70 to 230 nm. They investigated the use of water-based nanofluids that used magnesium oxide, copper oxide, titanium oxide, and iron oxide nanoparticles in a simple double pipe heat exchanger. These investigators used the two-step method for preparing the different nanofluids as this was, and is, the most commonly used for preparing nanofluids. This method is very proper and economical for producing nanofluids using the nanopowder technique for industrial production levels. The procedure for this method can be summarized as: obtaining the nanoparticles, nanofibers, nanotubes, or other nanomaterial as a dry powder produced by chemical or physical methods. Then the nanosized powder is suspended in a base fluid.

A new model for simulating the effective thermal conductivity of aluminum oxide (Al_2_O_3_) and titanium dioxide (TiO_2_) suspended in water and ethylene glycol was developed by Shukla et al. [120]. The authors introduced convective heat transfer due to the Brownian motion of spherical and non-spherical particles. It was concluded that the effective thermal conductivity of nanofluids is a function of volume fraction, nanoparticle diameter and shape, and temperature. The theoretical results suggested that a more pronounced increase in the effective thermal conductivity could be achieved using non-spherical nanoparticles having a larger volume-specific surface area.

Eshghinejadfard and Thévenin [121] numerically investigated heat transfer in particulate flow around an obstacle. They applied the Lattice Boltzmann Method (LBM) to analyze the heat transfer when a nanofluid flowed around an immersed sphere, using two and three-dimensional configurations in their simulation. They estimated the hydrodynamic force and energy exchange between the particle and the fluid through the direct heating immersed boundary (IB) method. The simulated results produced by this thermal IB-LBM accurately predicted the particle motion of nanofluids.

Bouguerra et al. [122] revisiting the influences of pH values and surfactant concentrations on the thermal conductivity of Al_2_O_3_-water nanofluids. The effect of production factors on the measured pH and stability of the Al_2_O_3_-water nanofluids was investigated. The results showed that the thermal conductivity is controlled mainly by the pH of the mixture, and maximum thermal conductivity can be achieved without adding surfactants. However, the nanofluid properties are best optimized by a combination of pH value, surfactant, particle size, and particle concentration, with the surfactant limited to less than about 0.03 wt.%.

**Figure 46:**
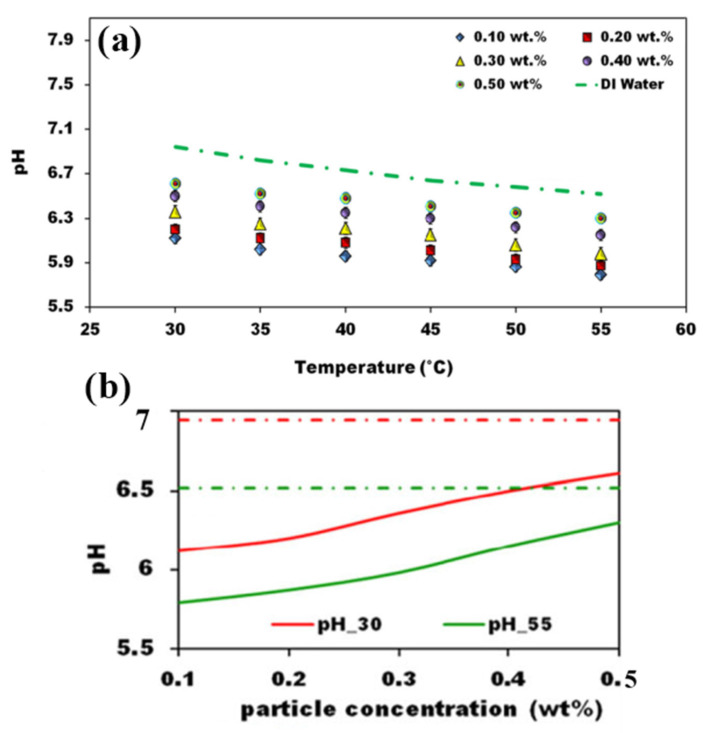
Changes in pH, where (**a**) shows the variation of the pH value of Al_2_O_3_ nanofluid at different nanoparticles concentrations, and (**b**) demonstrate the changes in the magnitude of the pH value at two different temperatures.

The effect of temperature and particle concentration on the pH of different nano colloids was investigated experimentally by Katiyar et al. [123]. Measurements showed that the pH of nano colloids is a strong function of temperature and the concentration of the dispersed phase, as shown in Figure 46. Charge transport mechanisms leading to changes in the effective proton population indicated that the stability of nanofluids is based on the concentration rate used and working temperature.

Tertsinidou et al. [124] presented a new technique for measuring the thermal conductivity of nanofluids and experimentally investigated how adding nanoparticles or nanotubes to a fluid improved the heat transfer performance and affected the viscosity. The nanofluids were CuO, TiO_2_, or Al_2_O_3_ nanoparticles in ethylene glycol, or TiO_2_, Al_2_O_3_ nanoparticles or MWCNTs in water. All measurements were performed at a temperature of 298.15 K. The results showed that, in general, the combined changes in physical properties that accompany the suspension of nanoparticles in fluids meant that the heat transfer benefits were all rather modest, even when they occurred.

Pryazhnikov et al. [125] in a similar paper reported measured thermal conductivity at room temperature for fifty nanofluids of various concentrations. The nanofluids were based on water, ethylene glycol, or engine oil as base fluid, and contained SiO_2_, Al_2_O_3_, TiO_2_, ZrO_2_, CuO, and diamond nanoparticles. The volume concentration of the nanoparticles ranged from 0.25 to 8.0 vol.%, and the particles size ranged from 10 to 150 nm. In agreement with Özerinç [104] it was concluded that classical theories do not accurately describe the thermal conductivity of nanofluids; instead, the nanofluid thermal conductivity coefficient is a complicated function of the particle concentration, particle size and material, and base fluid. Measured thermal conductivity coefficients almost always exceeded the values predicted by Maxwell’s formula. However, while nanofluids with sufficiently small particles may have thermal conductivity coefficients even lower than those predicted by the Maxwell theory, the thermal conductivity coefficient increased with increasing particle size in all cases. The authors found no direct correlation between the thermal conductivity of the nanoparticle material and the thermal conductivity of the nanofluid containing those particles. The base liquid also significantly influenced the effective thermal conductivity of the nanofluid. The lower the thermal conductivity of the base fluid the higher, relatively, the thermal conductivity coefficient of the nanofluid.

Poplaski et al. [126] measured the heat transfer properties of nanofluids containing Al_2_O_3_, CuO, and TiO_2_ nanoparticles, using a heat pipe. The concentrations of TiO_2_ and CuO were 25 vol.%, and for Al_2_O_3_, 35 vol.%. The maximum thermal heat transfer coefficient enhancement was 83%, 79%, and 76% for Al_2_O_3_, CuO, and TiO_2_, respectively. They also reviewed recent studies, focusing on the use of different nanofluids.

A new method for simulating the impact on thermophysical properties (particularly heat transfer and pressure drop) of different nanofluids was studied experimentally and numerically by Abdelrazek et al. [127]. They examined the momentum, thermal diffusivity, and thermal conductivity of Al_2_O_3_ and SiO_2_ in distilled water based on turbulent forced convection heat transfer. The volumetric concentrations were 1%, 2%, and 3%, with flow inlet temperature 30 °C. A two-dimensional model was used for numerical analysis based on the ANSYS-Fluent package. The standard k–ε turbulence model solved the continuity, momentum, and energy equations for a flow maintained in a Reynolds range between 6000 and 12,000. The results obtained were validated experimentally and showed that Al_2_O_3_ nanofluid enhanced the convection heat transfer coefficient more than other nanofluids of the same concentration. In contrast, Cu-distilled water nanofluids showed the lowest enhancement despite Cu having the highest thermal conductivity. As expected, the results showed that the kinematic and dynamic viscosities had the greatest effect on pressure drop in the fluid domain.

Das et al. [128] investigated the thermal conductivity and stability of nanofluids containing chemically synthesized nanoparticles for use in advanced thermal applications. This study presented an easy method of synthesizing, via a chemical route, silver (Ag) and copper (Cu) nanoparticles with a narrow size range in an aqueous medium under atmospheric condition at ambient temperature. They used Fourier transform infrared spectroscopy, field emission scanning electron microscopy, energy-dispersive x-ray spectroscopy, high-resolution transmission electron microscopy, UV–visible spectroscopy, and dynamic light scattering measurements. The experimental results showed the absence of any metal oxide layer around those nanoparticles that remained stable under ambient conditions. The behavior of some of the physical properties of the nanoparticles to be used for synthesizing effective nanofluids in a suitable base fluid via the chemical route were measured. These included particle size, concentration, stability, and conductivity. The thermal conductivity of the nanofluids with different nanoparticles was measured by the transient hot-wire method. The results indicated an increase in thermal conductivity with an increase in nanoparticles concentration, provided the initial nanoparticle concentration was below some limiting value, which depended on the type of nanofluid. The measurements showed that thermal conductivity increases markedly with an increase in temperature. It was suggested that these nanofluids are more suited for high-temperature applications.

**Figure 47:**
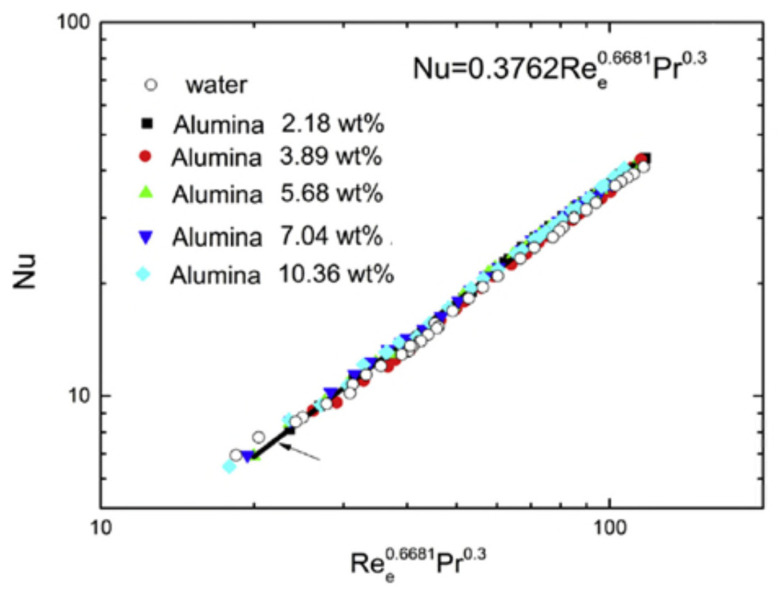
Nusselt number vs. Re Pr factor.

A similar study by Buschmann et al. [129] focused on the accurate measurement of the convective heat transfer coefficient of alumina nanofluid. The results showed that the Nusselt number increased as the number of nanoparticles in the base fluid increased; hence the convective heat transfer coefficient also increased. This was especially true in the case of Newtonian nanofluids, see Figure 47. These results demonstrated no anomalous phenomena in thermal conduction and forced convection-based heat transfer of nanofluids.

Bouguerra et al. [130] measured the thermal conductivity and dynamic viscosity of alumina/water-based nanofluids at concentrations, φ, ranging from 0.2 to 2.0 vol.% using a simple measurement simulator. The results showed that five dispersion regimes were identified based on the pH values for intermediate values of the volume concentration. The well-dispersed regime characterized by a local maximum of the thermal conductivity and an absolute minimum of the dynamic viscosity was not found for 2.0 vol.%, while the chain-like agglomeration regime was not observed for 0.2 vol.%. Although there are already many models for predicting the thermal conductivity of nanoparticle suspensions, most of them only consider the cases for high nanoparticle volume fractions (often 1.0 vol.%). The results obtained by Bouguerra et al. showed that for optimized nanofluids, comparable efficiency may be achieved at concentrations as low as 0.2 vol.%.

The prediction of the thermal conductivity of water-based oxide nanofluids with low volume fractions was investigated experimentally by Jin et al. [131]. The results showed that the famous multi-sphere Brownian model showed large deviations when predicting thermal conductivity for particle concentrations below 0.1 vol.%. The predicted thermal conductivity can be in good agreement with experimental data for the particle volume fractions ranging up to 10 vol.%. Liu et al. [132] studied the effect of SiO_2_, TiO_2_, and Al_2_O_3_ nanoparticles in water as the base fluid on the convective heat transfer coefficients. A double layer concentric glass tube was used for the study, allowing no contact between coolant and hot fluid. It was found that by adding nanoparticles, the convective heat transfer coefficient of water can generally be enhanced. A 45% increase was achieved with 0.5 vol.% of Al_2_O_3_ nanoparticles at an intermediate Reynolds number of around 4100. It was found that reducing nanoparticle size and increasing the nanofluid flow rate increased the improvement in heat transfer. It was concluded that the enhancement depended on the stability of the dispersed nanoparticles as characterized by their overall mean diameter and zeta (ζ) potential.

The stability of nanofluids is one of the major factors determining effectiveness, so the sedimentation and stabilization of alumina nanoparticles (size ~50 nm) in an ethanol-water mixture with and without dispersant was investigated experimentally by Abdullah et al. [133]. The sodium salt of polymethacrylic acid was used to measure the stability. The agglomeration of nanoparticles reduced the stability of nanofluids and consequently adversely affected their relevant properties. The investigators used sedimentary photographs and ζ -potential techniques to analyze the stability of the nanofluids and pH behavior. The results showed that sedimentation, ζ -potential, and photographic techniques corresponded well with each other. At low concentrations (0.03 mL), the stability of nanofluids was high due to the low rate of sedimentation of the nanoparticles.

**Figure 48:**
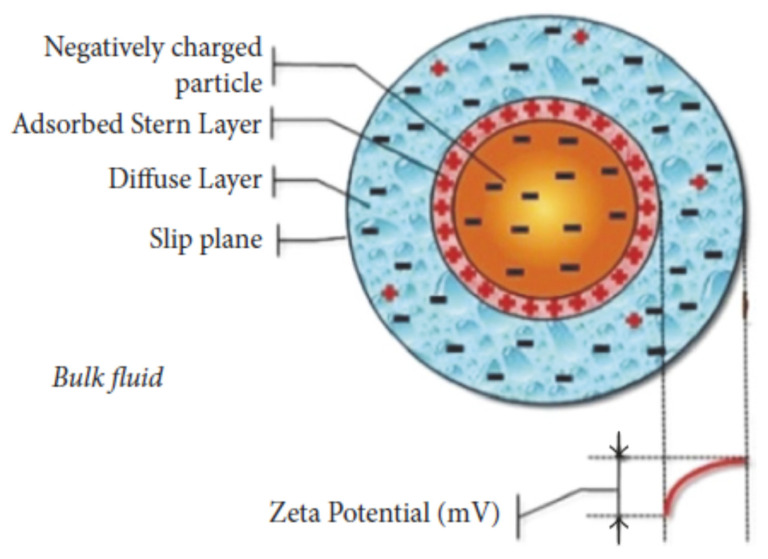
Zeta potential for nanoparticle [135].

Shah et al. [134] investigated the effect of bath ultrasonication time on the thermal conductivity, viscosity, and ζ -potential of Al_2_O_3_ nanofluids with concentrations of 0.2, 0.3, 0.4, and 0.5 vol.%. It was observed that the thermal conductivity of the nanofluids increased nonlinearly with increased sonication time/energy. At the same time, viscosity decreased, suggesting that the thermal properties of Al_2_O_3_ nanofluids are enhanced as the sonication time increased, demonstrating that Al_2_O_3_ nanofluids are amongst the most thermostable heat transfer fluids compared to conventional cooling fluids. The ζ -potential analysis was confirmed as an effective method of evaluating the stability of nanofluids through observation of their electrophoretic behavior, see Figure 48.

Nimdeo et al. [136] investigated the effect of temperature on the thermophysical properties of nanofluid suspensions using non-intrusive dynamic measurements. The study estimated thermal diffusivity based on temperature-position data for Al_2_O_3_-deionised water nanofluids. Enhancement in thermal diffusivity occurred with increasing temperature and concentration of Al_2_O_3_ nanoparticles in the nanofluid. The results showed that microscale mechanisms responsible for the anomalous behavior of thermal diffusivity could explain the measurement of ζ -potential, pH and effective viscosity of a dilute Al_2_O_3_ suspension at various temperatures. The measurements indicated that, the specific sizes and shapes, of the solid alumina particles had a significant effect on the improvement of the thermophysical properties of the nanofluid at a given temperature and Al_2_O_3_ concentration. The proposed non-intrusive measurement technique provided reliable measurements of the nanofluids’ thermal properties and gave results that were in agreement with measurements reported in the literature.

Chakraborty et al. [137] numerically investigated the effect of surfactant on the thermo-physical properties and heat transfer performance of a Cu-Zn-Al nanofluid. Both an anionic (Sodium dodecyl sulfate (SDS), concentration: 200–800 ppm) and a non-ionic surfactant (Tween 20, concentration: 28–70 ppm) were used. The results showed that the addition of the surfactants into the nanofluid suspension reduced surface tension and viscosity, which are highly desirable for better wettability and improved contact between the coolant and a hot surface. The maximum enhancement in thermal conductivity was 20.9% higher than for water in the case of Cu-Zn-Al nanofluid with 600 ppm SDS concentration. However, for the stability analysis, the highest ζ -potential value of −52.7 mV was observed for Cu-Zn-Al nanofluid at 800 ppm SDS. The highest cooling rate for the Cu-Zn-Al nanofluid was 174.8 °C/s with 600 ppm SDS, which is 30.7% higher than for water.

Wakif, et al. [138] numerically investigated the thermal properties of an alumina-water nanofluid for various sizes of spherical alumina nanoparticles (e.g., 30, 35, 40, and 45 nm) and different volumetric fractions (e.g., 0.01, 0.02, 0.03, 0.04 vol.%). They used Buongiorno’s mathematical model with simplified Maxwell’s equations and the Oberbeck-Boussinesq approximation for two-phase transport phenomena. Also, it was assumed that the rheological behavior of alumina-water nanofluid and related flow was Newtonian, incompressible, and laminar. The equation eigenvalues were found numerically using powerful collocation methods, such as the Chebyshev-Gauss-Lobatto Spectral Method and the Generalized Differential Quadrature Method. Furthermore, the thermo-magneto-hydrodynamic stability of the nanofluid system and the critical size of convection cells were highlighted graphically in terms of critical thermal wave numbers. The results indicated that stability increases for very small particles and increases in the nanofluid temperature but decreases as concentration and PH increase, especially at low temperatures.

### Carbon Nanotubes

The previous section covered literature relevant to the thermophysical properties of different nanofluids. In this section, the main focus is on Carbon Nanotubes, because these nanoparticles have very high conductivity and have recently attracted the attention of many researchers.

Carbon nanotubes (CNTs) are tubular carbon-based allotropes that can be produced from graphitic carbon and used in nanofluids for heat transfer applications. There are different types of CNTS, single-walled graphene cylinders (SWCNTs), double-walled (DWCNT) consisting of two nested cylinders, and multi-walled carbon nanotubes (MWCNTs) of several nested cylinders [139,140]. CNTs can be produced by decomposition of carbon-containing compounds such as C_2_H_2_, CH_4_, C_2_H_4_ or C_2_H_6_, over metal particles (catalysts), with growth occurring at the base or the tip of the tube in a needle-like structure. This procedure is based on using catalytic chemical vapor deposition. Chemical reaction time directly affects the length of the carbon tube, while tube diameter is determined by the diameter of the particle on which growth occurs [141]. The enhancement in thermal conductivity of working fluids used in thermal systems by introducing SWCNTs with a nanocoating of Ni was discussed by Wright et al. [142]. It was found that the nanofluid with coated nanotubes had a higher overall heat transfer coefficient due to the increase in the wall thermal conductivity. Wright et al. [142] fabricated nanofluids using self-assembly techniques and then measured the physical properties experimentally. This study considered nanofluid flow as frictionless transport and showed that properties such as thermal conductivity, viscosity, stability, acidity, and pH depended on nanoparticle concentration the techniques used to produce them.

Asadi and Alarifi focused on the effects of ultrasonication on the thermophysical properties of MWCNTs-water nanofluids [143]. Their results showed that increasing the ultrasonication time to 60 min leads to decreasing the dynamic viscosity of the samples. Based on their results, the maximum increase in fanning friction factor ratio is less than 3%, which indicates that adding MWCNTs to water does not significantly affect the required pumping power.

CNTs manufactured by catalytic decomposition typically show large thicknesses, often consisting of large particle aggregates. CNT production techniques were extensively researched, including arc-discharge, laser ablation, pyrolysis, and enhanced plasma vapor deposition. The main problem was the poor resulting dispersion, with high instability, which can lead to the agglomeration of the nanoparticles. To minimize the effects of poor dispersion, steric barriers (surfactants) are typically employed (Eastman [144]).

Goodarzi et al. [145] investigated the influence of different functional covalent groups on the thermophysical properties of a MWCNT-water nanofluid. Cysteine (Cys) and silver (Ag) were covalently attached to the surface of the MWCNTs. To determine the thermal properties, different water-based nanofluids such as Gum Arabic treated MWCNTs, functionalized MWCNTs using cysteine (FMWCNTs-Cys) and silver (FMWCNTs-Ag), were employed as coolants in a counter flow, corrugated plate HE. It was found that increasing the Reynolds number, Peclet number, or volume fraction improved the heat transfer characteristics of the nanofluid. At volume concentrations of 1.0%, there was minimal difference in the increase in heat transfer coefficients obtained at minimum and maximum Peclet numbers: the claimed enhancements were, 41.3073% and 41.3058% respectively.

The investigations conducted on plate HEs show how well nanofluids performed in terms of heat transfer characteristics. Based on the obtained results, it can be concluded that heat transfer coefficients increase with an increase in the volume fraction of nanoparticles, at least up to a certain percentage. Nevertheless, a few studies are not consistent with this finding and have concluded that heat transfer efficacy is enhanced by reducing the concentration of nanoparticles, but increasing Reynolds number and Peclet number. Because of their plates, plate HEs have more geometric parameters than other heat exchangers. For this reason, many of the studies conducted on the application of nanofluids in plate HEs have focused on their geometric characteristics.

Seth et al. [146] carried out research that simulated the influence of magnetic field and volume fraction of CNTs on the flow over a stretched sheet in a rotating frame, which is pertinent to gas turbines. The study analyzed the behavior of a base fluid of water with both MWCNTs and SWCNTs, using a mathematical model developed by order of magnitude analysis. The research included numerical simulation of the fluid velocity, temperature, Nusselt number and skin friction coefficient for a set of values of different regulatory flow parameters to replicate the physics of the flow. The research concluded that the fluid velocities for SWCNTs and MWCNTs are lowered with incremental changes to the governing flow parameters, such as porosity and inertia coefficients. The investigators pointed out that nanoparticles used ranged in size from 1 to 100 nm.

Given that the thermal performance of conventional working fluids such as oil and water is poor, nanofluids with strong thermal properties can greatly improve thermal transfer. It was found that there was a reduction in primary fluid velocity with both SWCNTs and MWCNTs due to an increase in rotational parameters. It was also found that the fluid temperature was enhanced for both SWCNTs and MWCNTs with an increase in the nanoparticle volume fraction. The primary skin friction coefficient was also enhanced by strengthening the regulatory flow parameters. The study revealed that there was a significant increase in thermal energy irreversibility, as measured by the Bejan number, near the stretching surface. The conclusion drawn from these findings is that the presence of an applied magnetic field when using a nanofluid containing either SWCNTs and MWCNTs increases the thermal energy’s irreversibility in terms of Bejan number.

Hayat et al. [147] maintain that as CNTs comprise rolled graphene sheets, the material consists of carbon chains that do not pose a risk to the atmosphere. The hexagonal carbon molecules have significant properties that can be exploited for heat transfer, including thermal conductivity. They also exhibit high levels of corrosion resistance and significant mechanical strength, and electrical conductivity. These properties mean that CNTs are useful in a wide range of applications, such as catalyst supports and energy storage, sensors, and thermal material.

Arzani et al. [148] studied the thermophysical properties, including the heat transfer rate, of covalently functionalized MWCNTs-nanofluids in an annular heat exchanger as used in gas turbines. The study aimed to develop a novel method for improving their performance with respect to heat transfer effectiveness by preparing highly-dispersed carbon nanotubes. The project used aspartic acid, acting as a hydrophilic group, to functionalize the MWCNTs to produce a highly dispersed colloidal suspension covalently. The MWCNTs were stirred in a mixture of H_2_SO_4_–HNO_3_ acids with volume ratio of 1:1 for 24 h at a temperature of 60 °C. The solution was then filtered and rinsed with deionized water, after which the filtrate cake was dried. Then, 1 g of oxidized MWCNT and 1 g of aspartic acid were placed in 200 mL DMF with 10 mL toluene for 2 h and then stirred for 12 h, using ZrCl_4_ (5% mol) as a catalyst to increase the rate of the reaction. The resulting suspension was then filtered and rinsed with deionized water and dried in the oven for 48 h at 50 °C. To synthesize the MWCNT-Asp/water nanofluids, the MWCNT-Asp was initially dispersed in deionized water for 1 h using an ultrasonic probe. Arzani et al. [148] assessed the dispersion state as well as the long-term stability of the MWCNT-Asp/water coolants for different weight fractions and concluded that there is an insignificant decrease in the relative concentration of MWCNT-Asp over time, which, they argued, confirmed the suitable dispersibility of MWCNT-Asp in water.

The results also showed that the density of the prepared MWCNT-Asp/water nanofluid increased with increased temperature and concentration. The results again confirmed that using a nanofluid improved heat transfer performance compared to water. Generally, the thermal conductivity of the nanofluids improved with increasing MWCNT-Asp, and it was also evident that as the temperature increased, there was a corresponding increase in thermal conductivity.

Imtiaz et al. [149] also examined the flow and heat transfer between two rotating and stretchable disks using nanofluids containing SWCNTs and MWCNTs with water as base fluid. The simulations assumed convective boundary conditions between the two disks. It was concluded that the magnitude of the radial velocity of the fluid decreased near the surface of the disks for increasing values of Reynolds number. It was also noted that water-based SWCNTs produced less drag and exhibited a higher heat transfer rate than water-based MWCNTs. However, there was a greater enhancement in fluid temperature for the increased volume fraction of the MWCNT nanoparticles than for SWCNTs.

Karthikeyan et al. [150] investigated the wetting behavior of MWCNTs. The study analyzed thermophysical properties such as surface tension, stability, acidity, thermal conductivity, and viscosity. The surface tension and wetting behavior were investigated for MWCNT nanofluids dispersed in reverse osmosis water and 99% anhydrous ethanol. The main conclusions from this study were the surface tension, and wetting behavior of the stable aqueous and ethanol-based nanofluids containing plasma functionalized MWCNTs were unaffected by MWCNT loading of up to 0.012 and 0.021 vol.%, respectively. With MWCNT-ethanol-based nanofluids, there was a linear increase in surface tension with over 0.02 vol.% of MWCNT. However, the nanofluid’s stability and the stabilization method used were important parameters in determining the wetting behavior of the nanofluids at low temperatures and high concentration levels.

**Figure 49:**
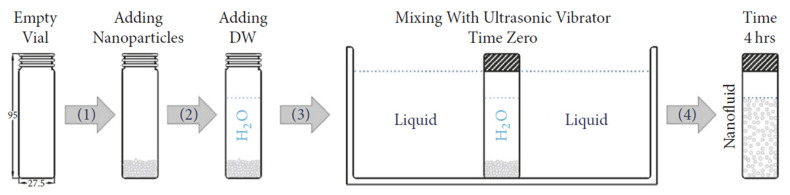
Flow chart of two-step nanofluid preparation [135].

Ali et al. [135] confirmed that a major factor affecting the thermophysical performance of nanofluid is its stability and this is strongly affected by the fabrication method and presented a summary of most methods used in the fabrication of nanofluids. They showed that most investigators and engineers used the two-step procedure to prepare nanofluid using aqueous media. The main steps of this procedure are shown in Figure 49. Ali et al. [135] also measured the stability and other thermophysical properties of a range of nanofluids. It was found that stability increased when using very small particles in the nanofluid. Stability improved in high ambient temperatures and decreased in low. As the acidity of aqua-nanofluids increased, the stability decreased, and sedimentation occurred, which led to the need to increase pumping power, a major disadvantage.

A recent study by Sajid and Ali [151] discussed advances in the application of nanofluids in heat transfer devices; their advantages and drawbacks were given and analyzed. This was a critical review of the heat transfer applications of nanofluids from numerous studies and included the effects of nanoparticle concentration, size, shape, the effect of nanofluid flow rate on Nusselt number, heat transfer coefficient, thermal conductivity, thermal resistance, friction factor, and pressure drop. Also reviewed were the effects of various geometric parameters on the enhancement of heat transfer in heat exchangers. Various correlations used for experimental validation or developed in reviewed studies were presented, compared, and analyzed. It was concluded that the heat transfer and pressure drop characteristics resulting from the application of nanofluids are subject to a large number of parameters, including particle morphology (shape and size), nanoparticle type, nanofluid preparation method, surfactants, stability of nanoparticles in the base fluid and, more significantly, on volume concentration of nanoparticles and flow rate of the nanofluid. It appears there is an optimum concentration of nanoparticles that leads to optimum stability, heat transfer, pH, and thermal conductivity.

Most investigators have indicated an improvement in heat transfer rate with an increase in flow rate of the nanofluid due to a boost in forced convection due to increased turbulence in the fluid, better mixing, and inter-collision of nanoparticles. A few studies recommended using very small nanoparticles, claiming better effect heat transfer performance and less friction. The friction factor is increased with the increased density and viscosity of the nanofluid. It is apparent from the above studies that nanofluids have massive potential in practical applications. However, many aspects require more in-depth research, such as the effects of the addition of surfactant on the heat transfer characteristics of nanofluids, which presently are not well understood or explained. Measured accurate thermal conductivity, stability, pH, density, and nanofluid viscosity are not yet possible.

## 7. Gaps in Present Knowledge

To produce compact, high-performance heat exchangers suitable for use in the propulsion system of a marine gas turbine, it is necessary to enhance the internal heat transfer of the coolant fluid. Although many research studies have investigated the use of nanofluids in heat exchangers to enhance thermal conductivity, there exists plenty of controversy and inconsistencies among the reported results. Many challenges face the successful applications of nanofluids.

The conclusion of most heat transfer investigations that used nanofluids is that the understanding of the behavior of nano-particles in base-fluids is still quite limited, especially their stability and thermo-chemical properties. Also, the most suitable concentration of nanoparticles to use with a particular base fluid needs investigation, as does the optimum velocity flow in given heat exchangers, such as those that might be applied to WR-21 gas turbines. Other questions that need answering include: what nanofluids can be safely used in an intercooler, what is the optimum concentration of nanoparticles, what corrosion is a particular nanofluid likely to cause, what operating conditions will cause the least fouling, what measures can be taken to minimize fouling when using nanofluids, what should be the flow Re number, what the best intercooler effectiveness to use with nanofluids? Also, the effect of nanoparticles on the friction factor of flow through intercooler tubes needs significant experimental investigation.

The intercooler technologies used in gas turbines are well-established, but there is still an ongoing demand for more compact, low-pressure loss, more effective heat exchangers. Intercoolers are mainly deployed in stationary and marine gas turbines, but bulky intercooler installations limit their applications. Increasing the size of an intercooler may require an increase in space occupied by the engine this can increase installation losses and add to shaft lengths, which increases the likelihood of vibration problems.

In most of the literature reviewed, one way was used to produce the nanofluids to be used as a heat transfer medium. The preparation is crucial as it can result in a completely different nanofluid in terms of its thermophysical properties (i.e., acidity, settling behavior, and particle agglomeration) but it is noted that there is a lack of agreement on controlling the temperature of the base fluid containing the nano-particles during the preparation phase when using an ultrasonicator. Using an ultrasonicator for fabricating nanofluids generally produces a gradual increase in the temperature of the prepared fluid, which is strongly influenced by the surrounding ambient temperature, which may vary with time of day and season of the year. This variation can result in the production of nanofluids of diverse thermophysical properties.

A key finding in most of the literature reviewed was that the use of nanofluids in applications at elevated temperatures occasionally resulted in ‘nano-fouling’ the deposition of the nanoparticles on the surface in contact with the fluid. This type of particle build-up has a strong impact on both wettability of the surface, its thermal conductivity, and surface roughness. The latter can directly impact the pumping pressure and can be a big problem. Therefore, an investigation is required of nanofluid fouling problems as a function of the fabrication method, particularly by controlling the operating temperature. Experimental research using simulated rigs or directly with large-scale equipment such as a WR-21 engine is needed to assess whether some nanofluid particles will build up and accumulate on the surface of the HE tubes after a short time of operation. It is also required to research and fabricate other types of nanofluids that can be used safely and effectively in intercoolers and recuperators with high operating temperatures.

There is a limited number of investigations in the reviewed literature regarding CNT nanofluids, with little in terms of the practical application of CNTs as working nanofluids in intercoolers after the first compressor. Reports show CNT nanofluids lead to significantly higher heat transfer enhancement than other nanofluids and could maximize efficiencies when used in the intercoolers of a WR-21. Studies of CNTs in plate-fin HEs are still needed to better understand the thermophysical processes involved. There have been some recent studies into heat pipes and shell-and-tube HEs that can be applied to recuperators. Many researchers have used Cu, Fe_3_O_4_, and Al_2_O_3_ particles as secondary materials in HEs operating in low-temperature ranges.

Questions regarding using CNT nanofluids in the intercooler of a WR-21 still need to be posed and answered. The reported research strongly suggests that using nanofluids can improve thermal transfer, reduce pressure drop, while they remain stable. The impact depends on the vol.% of nanoparticles in the nanofluid and the base fluid density.

Finally, it is clear there is a significant gap in current knowledge on the use of nanofluids as a secondary heat transfer material, especially in intercoolers (plate-fin HE) used in the application of marine gas turbine power plants. It is hoped that this review has, at least, addressed part of this gap. So, a primary aim of current research being undertaken at Cranfield University, School of Aerospace, Transport and Manufacturing will be to investigate experimentally and numerically the application of CNT nanofluids to convective heat transfer in plate-fin HEs (intercoolers) for use in marine gas turbine power plants operating at high temperatures.

## 8. Conclusions

This review has covered aspects of gas turbine technology, mainly the introduction of intercoolers with advanced working fluids in the form of nanofluids to the cycle. As such, the main conclusion is divided into three parts, as follows:

### 8.1. Gas Turbine Technology

Designing a complete marine gas turbine system is complex due to the many interactions involving consideration of different and sometimes conflicting physical, chemical, material, and design requirements. Military and commercial competition ensures continuous improvement in performance and operating conditions and present designs are the result of decades of research and development and contain several commonly used arrangements, including the simple cycle, which remains the standard layout; the intercooler cycle, which adds an intercooler to the standard cycle for increased performance; the ICR cycle, which uses a recuperator to recover heat from the exhaust of the gas turbine; the reheat cycle, which adds a reheat combustor to the standard cycle; an intercooled reheat cycle, which is a combination of intercooled and reheat cycle configurations; and combined cycle. The review found that the ICR is optimal for marine applications and is used in the WR-21. On the other hand, Gradient-based optimization techniques seem to provide a solution. These techniques rely on gradient information; how the performance changes when infinitesimal changes to the design variables are made [152].

Most previous works were based on increasing compression, turbine inlet temperature, and overall pressure ratio. But these factors are limited because they depend on the specifications of the materials used, cooling technology, and cost. However, analytical results showed that maximum thermal efficiency required entropy generation per unit work output to be a minimum, which required improvement in the Brayton cycle operation and design parameters such as the effectiveness of the intercoolers and recuperators. It has been shown that small value of recuperator effectiveness means the thermal efficiency of the cycle would also be small.

Using an intercooler in marine gas turbine propulsion plants is very effective due to its direct effect on the overall efficiency of the cycle, whereas decreasing the work input to the compression process requires very high recuperator effectiveness. However, using intercooling in a gas turbine propulsion system without reheat causes a decrease in efficiency (at least for small pressure ratios) due to the drop in air temperature after the compressor. Overall results show that reducing the net specific work input required for a given pressure ratio improved the performance of the cycle. As a general case, the intercooler benefits the Brayton cycle gas turbine power plant by decreasing the compression work of the high-pressure compressor, which allows for higher pressure ratios, thus increasing overall efficiency.

The current direction of marine power system development is taking a different direction such as design specification, manufacturing efficient energy systems, and using better operating conditions.

The WR-21 is a development of the aero and industrial RB211 family of engines and meets current Royal Navy ICR requirements. New designs based on the WR-21 using efficient, super materials for the gas turbine blades will enable higher engine capacities for cargo vehicles. An essential element in developing the WR-21 could be to use nanofluids to improve thermo-fluid flow performance and reduce overall costs.

### 8.2. Heat Exchanger

The gas turbine plays a key role in power generation, with its basic cycle involving three main components: compressor, combustion chamber, and turbine. The overall performance of the gas turbine depends on the efficiency of each component and the working temperature of the turbine. An attraction of such a design is that while each component affects the efficiency of the overall unit, it is possible to develop the different components gas turbine separately. For example, improving the performance of the heat exchangers (e.g., intercooler and recuperator) will increase the power output and efficiency. However, any changes of an individual component will impact the overall performance, such as increasing design complexity, weight, size, cost, and raising manufacturing, maintenance, and safety issues.

An important element in the intercooler is choosing the heat exchanger from various types, materials, and configurations. The selection depends on the heat transfer rate required and the base fluid. Considerable research has been carried out to identify and select the optimal type of heat exchanger. However, the process is complex, and further research is needed to reduce the HE weight and size while simultaneously improving its efficiency, especially in marine and aero-technology applications. The main factor for classifying a HE is its effectiveness rating used to estimate its thermo-hydraulic performance. The design parameters of HEs have usually been based on heat exchanger geometry and material properties. Recently operating conditions such as working fluid flow rate, temperature, thermal conductivity, stability, and viscosity (pressure drop) have become important.

There are different ways to increase HE performance, such as increasing the heat transfer rate between the two sides in indirect systems using one of the following: increasing the heat transfer coefficients, increasing exchanger surface area, increasing working fluid thermal conductivity, and increasing the temperature gradient. For example, a shell-and-tube HE increases the tube-side heat transfer coefficient by increasing the fluid velocity or increasing the number of tube passes. The shell-side heat transfer coefficient can be increased by decreasing baffle spacing and/or baffle cut. It may be possible to increase the heat transfer surface area by increasing the length of the HE, increasing the shell diameter, or using multiple shells in series. On the other hand, using alternative working fluids is very effective, especially nanofluids where Fe_3_O_4_, Cu, CNTs, etc., nanoparticles are added to a working fluid to improve its thermophysical properties.

Extensive experimental and theoretical efforts have been made to obtain solutions for turbulent forced convection heat transfer and flow friction problems in HEs. There are many empiric correlations available in the literature for fully developed (hydro-dynamically and thermally) turbulent single- and two-phase flow with constant physical properties of the fluids. These existing correlations can be used in the design of HEs subject to the limitations imposed on their applicability. Also, much scientific effort has been put into determining empirical correlations that give good agreement with experimental data and can be used to estimate heat transfer coefficients and the effectiveness of HEs directly. In addition, applying these correlations should not increase the pressure drop across the HE. Reducing the pressure drop can be achieved by decreasing the number of tube passes, increasing tube diameter, decreasing tube length, using very smooth material, optimizing the nanofluid concentration, and/or minimizing the Renumber.

Most published literature covers methods of increasing HE effectiveness. Still, the main limitation of heat transfer enhancement techniques is the poor thermal performance of the working fluids, obstructing increases in performance and compactness of HEs. The fundamental thermophysical properties of fluids are convective heat transfer, thermal conductivity, viscosity, and specific heat at constant pressure. Of the available literature, most studies have focused on forced convective heat transfer coefficient and Nusselt number, which depends on the Prandtl and Reynolds numbers of nanofluids in HEs consisting of circular tubes. The convective heat transfer coefficient depends on thermal conductivity, specific heat capacity, viscosity, flow rate, and density of the fluids. The fluid flow regime and temperature are of utmost importance, and studies have been conducted for both laminar and turbulent flow regimes.

### 8.3. Nanofluids Technology Subsection Shell-and-Tube Heat Exchangers

Most nanofluids used in heat transfer and energy conversion systems such as HEs are used to enhance the thermophysical properties of the working fluids such as water, air, and oil. The added nanoparticles improve the thermophysical properties such as thermal conductivity, specific heat, and heat capacity. Still, they can have negative effects by increasing the viscosity of the base fluid, increasing the friction factor, and fouling the system with sediment, all of which increase the pressure drop across the heat exchanger and require additional pumping power to circulate the working fluid at the required rate.

There has been significantly less attention given to investigating the convective heat transfer of nanofluids and possible enhancement factors needed to enable the practical application of nanofluids to heat exchangers with high working temperatures and pressures such as in intercooler-recuperators of gas turbine systems. Of the limited number of available studies, most report relative enhancement of convective coefficients with respect to thermal conductivities for the same nanofluid. This is a strong indication that other, possibly as yet unknown, factors influence the enhancement of heat transfer obtained by using nanofluids. It is observed that all experimental studies of convective heat transfer studied nanofluid flow through circular tubes and agreed that the particle volume fraction influences thermal performance. However, higher particle concentrations contribute to a higher pressure drop across the HE for the same fluid flow rate, which carries the inconvenience of increased pumping requirements. So, using nanofluid in the intercooler-recuperator of a Brayton gas turbine cycle will require considerable investigation, especially practical studies.

### 8.4. Future Work

Based on the literature review, researchers should focus on measurement methods and technologies for the accurate measurement of the thermophysical properties of nanofluids. Also, simulations should be carried out based on the measured values and more realistic assumptions to better determine the main factors that affect working fluid behavior.

## Figures and Tables

**Table 1:** Pressure ratio with turbine inlet temperature for compressor inlet temperature 288.15 K [8].

TIT (K)	${\dot{W}}_{net}-PR$		$\eta_{cyc}\left(\%\right)$
	${\dot{W}}_{net}\left(kW\right)$	*PR*	
1000	222.355	6	31.42
1100	280.741	8	34.63
1200	341.54	10	36.93
1300	404.124	12	38.65
1400	466.891	16	40.94
1500	528.603	18	41.75
1600	586.712	22	43.00

**Table 2:** Turbine and compressor inlet temperatures for maximum thermal efficiency [8].

CIT (K)	${\dot{W}}_{net}-TIT$		$\eta_{cyc}\left(\%\right)$
	${\dot{W}}_{net}\left(kW\right)$	TIT (K)	
273.15	36.3	1100	284.25
283.15	35.72	1100	271.31
273.15	36.96	1200	347.350
283.15	36.49	1200	334.376
273.15	37.22	1300	408.411
283.15	36.82	1300	395.404
273.15	37.18	1400	466.085
283.15	36.83	1400	453.045
273.15	36.9	1500	518.860
283.15	36.59	1500	505.792

**Table 3:** A summary of the reviewed literature on the parameters affecting gas turbine performance (thermal efficiency).

Reference	Study Method	Studied Parameters	Concluding Remarks
Cetin [4]	Numerical	Compressor inlet temperature, turbine inlet temperature, maximum pressure and temperature, and heat exchanger effectiveness	Decreasing the air inlet temperature increased the net power of the simple cycle, and increasing the inlet turbine temperature improved the plant’s thermal efficiency.
Xie et al. [7]	Numerical	Inlet temperature, pressure ratio, gas specific heat, and gas turbine performance	Decreasing compressor work by reducing the specific volume of inlet air to increased net power of the cycle and increased thermal efficiency.
Kurt et al. [8]	Numerical	Pressure ratio, thermal efficiency, and power output of the cycle	The studied parameters have significant effects on gas turbine performance, such as net power output, thermal efficiency, and fuel consumption of the cycle
Shah [9]	Numerical	The thermal efficiency of microturbines	Using a recuperator with an effectiveness of 87% raised the efficiency of the gas turbine system to 30% approximately
Shi and Che [6]	Experimental	Gas turbine inlet temperature, condenser pressure, fuel gas heating temperature	Heating the fuel resulted in higher gas turbine efficiency.
Barinyima et al. [11]	Experimental & Analytical	Inlet air temperature, fuel-specific heat, effectiveness, heat ratio, inlet turbine temperature, compressor efficiency, and turbine efficiency	To a large extent, the modified engine with unconventional components exhibited better thermal efficiency and specific fuel consumption performance than traditional simple-cycle engines.
Zhang and Zhou [12]	Experimental	The spray characteristics of an air-blast atomizer unit to minimize the emission of flue gases from a gas turbine combustor	The method reduced fuel consumption and increased the net power, directly improving the performance of the gas turbine.
Guven et al. [13]	Thermodynamic modeling	The effect of using different fuels in the gas turbine on the energetic and exergetic gas turbine performance	Methane had the maximum exergy efficiency and the minimum exergy destruction, while methanol had the minimum exergy efficiency and maximum exergy destruction
Wang and Pan [14]	Numerical	Gas turbine performance power output, thermal efficiency, air-fuel ratio, and heat transfer	The thermal efficiency was directly increased due to the increase in the turbine’s power. Generally, the high-pressure intercooled cycle gave a better performance than the simple Brayton cycle.
Gad-Briggs et al. [15]	Experimental & Analytical	The effect of cycle inlet temperature on the precooler and plant thermal efficiency	In the case of using the intercooler and recuperator, the thermal efficiency is higher than for the cases of IC alone and SCR.
Guo et al. [16]	Experimental	The intercooled Brayton gas turbine cycle’s irreversibility	Decreasing the specific heat of the fuel gases increased the intercooler’s pressure ratio, which affected the power output and performance.
Yang et al. [17]	Numerical & Analytical	The performance of a regenerative-intercooled Brayton gas turbine cycle	The study proposed analytical dimensionless formulas for exergetic efficiency.
Al-Doori [18]	Numerical	The effect of using an intercooler on the parametric performance of a gas turbine power plant	It was found that adding an intercooler increased the plant’s power output by decreasing the compressor input work, which resulted in increasing the thermal efficiency of the gas turbine power plant.
Wen and Dong [20]	Numerical	The intercooler’s performance was investigated based on its design and operational parameters	Increasing the effectiveness of the intercooler led to a decrease in compressor work. Moreover, turbine work was decreased due to reduced turbine inlet temperature, which then decreased thermal efficiency.
Ying et al. [21]	Numerical & Experimental	Thermodynamic parameters and performance factors for different marine gas turbines	An increase in flow rates of seawater and glycol-water solution coolants could improve the performance of the intercooled-cycle gas turbine, but the ratio of the flow rates should be chosen according to the atmospheric conditions and seawater temperature.
Arai et al. [24]	Numerical	Performance of a micro gas turbine system	Optimizing the angle of the impeller blades had a direct effect on the power extracted from the fuel gas, improving the power output of the cycle, increasing the thermal efficiency, and decreasing the specific fuel consumption.
Zhang and Gümmer [25]	Numerical & Experimental	Thermal efficiency	The thermal efficiency reached more than 60%.
Kim and Hwang [23]	Numerical	The performance of recuperated gas turbines considering engine configuration and operational strategy	Increasing the heat recovery from exhaust gases by using a recuperator device for turbines under part-load.
Ferreira and Nascimento [26]	Numerical	The performance of a gas turbine driven by biomass fuel	The performance changed when the engine ran on syngas, with the running line in the compressor characteristic moved towards the surge line.
Ji et al. [27]	Numerical	The phased missions of ships, operational flexibility, and mechanical and thermal constraints	The control strategy at steady-state conditions for the intercooled gas turbine should be a variable cycle control.
Soares [28]	Experimental	Factors affecting the WR-21 operation cycle, including ambient temperature, pressure ratio, air inlet temperature, and air mass flow rate	The exit temperature of the intercooler was increased by reducing the LP compressor surge margin due to decreased mass flow.
Maher et al. [29]	Mathematical	The parametric performance characterization of an irreversible gas turbine Brayton cycle	The exclusive effect of each operating parameter on each of the performance parameters is given in a general mathematical formulation.

**Table 4:** A summary of the reviewed literature on the parameters affecting the performance of different HEs.

Reference	HE Type	Studied Parameters	Remarks
Mukherjee [38]	Shell-and-tube	Thermal effectiveness of shell-and-tube heat exchangers	A design procedure for the shell, tubes, tube pitch, baffles, and layout patterns was presented.
Suri et al. [39]	Reviewed different HEs	Roughness elements used to enhance heat transfer rate	It was noted that many experimental and analytical studies in the literature concentrated on the analysis of heat transfer coefficient and pressure drop optimization.
Lunsford [40]	Shell-and-tube	Methods of increasing the performance of shell-and-tube HEs	If a larger pressure drop across the HE was permitted, augmented surfaces could be used to enhance heat transfer.
Zueco et al. [41]	Shell-and-tube	Performance factor	Software developed for for use in the design of shell-and-tube heat exchangers
Edwards [42]	Shell-and-tube	Thermal design parameters such as type of flow, tube arrangement, tube diameter, thickness and length, and shell size	The effect of the studied parameters on effectiveness, pressure drop, heat transfer coefficients, heat transfer rate, and fouling rate were reported.
Bouhairie et al. [43]	Shell-and-tube	Thermal design parameters	The study focused on the flow behavior in the window between baffle compartments to reveal the complexity of shell-side flow dynamics around the tube bundle.
Ghaith et al. [44]	Shell-and-tube	How to enhance the thermal design of a shell-and-tube heat exchanger	The results showed that the critical area to provide a thermal duty of 1.4 MW was about 1132 m^2^ with tube-side and shell-side heat transfer coefficients of 950 W/m^2^K and 495 W/m^2^K, respectively.
Wan et al. [45]	Shell-and-tube	Thermal failures generated in the tube-to-tube sheet joints of a shell-and-tube heat exchanger	The results showed that large tensile residual stresses above yield strength could be generated in the tube-to-tube sheet joints. The residual stresses at the bottom surface and the edge of the tube sheet are relatively small, even becoming compressive.
Pint et al. [48]	Plate-Fin	The effect of the water vapor found in exhaust gases on the oxidation resistance of materials commonly used for recuperators	The report recommended the use of expensive superalloys for manufacturing heat recuperators used at very high temperatures or used with water on either side to minimize overall operating costs.
Lara-Curzio et al. [49]	Plate-Fin	Design of a recuperator that operates at high temperatures	Different materials were tested, and the final material recommended for manufacturing microturbines is a ceramic.
Fabio et al. [50]	Plate-Fin	Types of working fluids	It was found that the working fluid should be of low viscosity to decrease the pump power needed for circulation, that using very smooth material for the plate HE decreased the pressure drop and reduced flow distribution problems, allowing the use of a working fluid of greater viscosity.
Arsenyeva et al. [51]	Plate-Fin	Analyzing heat transfer within channels of complex geometry	The study concluded that it is difficult to achieve optimal performance with the plates currently on the market and as such, further research is required into the design of these exchangers.
Bichnevicius et al. [52]	Plate-Fin	The performance of a louvered plate-fin heat exchanger, comparing traditionally manufactured and additively manufactured HEs based on the same design.	The study found additively manufactured HEs varied in performance even when printed using the same digital model.
Karvinen and Karvinen [53]	Plate-Fin	Effects of geometry to maximise heat transfer	A new method for determining plate-fin geometry for maximizing heat flux was presented.
Kim and Rhee [54]	Plate-Fin	The heat transfer performance and space efficiency of compact heat exchangers	The research found that the optimum cross-cut length for wavy fin heat exchangers varied with the corrugation angle; an increase of corrugation angle had a negative impact on the overall thermal performance in laminar flow.
Manglik et al. [55]	Plate-Fin	The effect of pins on the performance of a plate-fin HE	Increasing fin density and using shorter fins produced a convection performance that is virtually the same as that with 100% fin efficiency.
Han et al. [56]	Fin-and-tube	The effect of different shaped tubes on the fluid flow and the heat transfer characteristics of finned tubes heat exchanger	The overall conclusion was that an oval fin-tube reduced flow resistance and improved the heat transfer capacity of the heat exchangers, with a corresponding improvement in fin efficiency.
Nasrabadi et al. [57]	Plate-Fin	The effect of thermo-hydraulic parameters on the efficiency of three heat exchangers	The fin plate heat exchanger was the optimum choice for gas turbine power plants.
Tarrad et al. [58]	Plate-Fin	Predicting the air-side heat transfer coefficient	The study found that a crimped spiral fin had higher heat transfer and pressure drop than the other types of fins
Yang et al. [46]	Plate-Fin	Heat transfer performance	The study indicated that it was not possible to attain a uniform heat transfer coefficient over all the surfaces in the fin channel, which is the basis of conventional concepts.
Zhou and Yu [59]	Plate-Fin	The heat transfer characteristics of a falling film type plate-fin heat exchanger	Different important factors were considered; liquid film thicknesses, local heat transfer coefficients of oxygen and nitrogen, the total local heat transfer coefficient, and the effects of inlet mass flow rate of oxygen liquid over the base plate to that over the fin surface, on the wetted length of the heat transfer surfaces.
Chordia et al. [60]	Plate-Fin	Designing and testing a high-temperature plate-fin HE	Measured results for the recuperator confirmed the predictions of the heat transfer models used.
Abou Elmaaty et al. [61]	Plate-Fin	Reviewed the performance of plate-fin HEs used as recuperators with gas turbines	It was concluded that plate-fin HEs work efficiently in both single and two-phase flows, though two-phase flow still requires considerable further research.

**Table 5:** A summary of the reviewed literature on the parameters affecting the performance of different HEs.

Reference	Nanofluid	Studied Parameters	Remarks
Zhao et al. [73]	Al_2_O_3_-water Cu-water	Heat transfer performance	It was found that the nanofluids improved the heat transfer performance compared to water.
Bahiraei et al. [77]	Different water-based nanofluids	Reviewed the effects of different nanofluids on heat exchanger performance	It was concluded that using nanofluids increased the heat transfer performance.
Hosseini et al. [79]	MWCNT-water	Thermal and hydrodynamic properties	The results indicated that the addition of a small fraction of MWCNTs into distilled water significantly enhanced the convective heat transfer coefficient.
Baba et al. [90]	Fe_3_O_4_–water	Forced convective heat transfer and pressure drop in a double tube counter flow HE	The results showed that the heat transfer rate increased by 80–90% for the same operating conditions when the finned tube HE replaced the non-finned tube HE.
Kumar et al. [83]	ZnO/water	The effect of chevron angle on heat transfer performance of a plate heat exchanger using a nanofluid	It was reported that there was an optimum enhancement in heat transfer rate ratio, heat transfer coefficient ratio, and optimum reduction in exergy loss obtained at angles of 60°/60° for 1.0% particle volume concentration of ZnO/water nanofluid.
Heris et al. [84]	CuO-water Al_2_O_3_/water	Heat transfer performance	The study found that the presence of nanoparticles enhanced the heat transfer process, with the heat transfer coefficient increasing with the increased concentration of nanoparticles in the nanofluid.
Tiwari et al. [85]	CeO_2_-water	Heat transfer performance and pressure drop	It was reported that the nanofluid in the plate HE achieved a 39% greater heat transfer coefficient than water at an optimum concentration of 0.75 vol.%. The pressure drop with the nanofluid was approximately the same as for water up to optimum volume concentration
Anoop et al. [86]	SiO_2_–water	The thermal performance and thermo-physical properties of nanofluids	The results showed that heat transfer coefficients for nanofluids depended on the flow rate through the heat exchangers and nanofluid concentration. Also, it was reported that replacing water as the working fluid with the nanofluid increased the pressure drop.
Malekan et al. [87]	Fe3O4-water	The effect of applying a magnetic field on the heat transfer of a magnetic nanofluid	The convective heat transfer coefficient of the nanofluid increased when the volume fraction of the nanoparticle increased, enhanced by turbulent flow induced by the magnetic field. However, increasing either the volume fraction or magnetic field increased the pressure drop and friction factor on the nanofluids side.
Maddah et al. [88]	Al_2_O_3_/TiO_2_-water	The thermal performance of a double pipe heat exchanger using a hybrid nanofluid	It was found that using nanofluids significantly enhanced the performance of the heat exchanger.
Nakhchi and Esfahani [89]	Cu-water	Heat transfer	Using nanofluids with higher Re numbers increased the rate of heat transfer between the two sides of the HE due to increased convective heat transfer and thermal conductivity.
Bahiraei et al. [90]	Graphene-water	Effects of using nanofluid on thermal and hydraulic characteristics of a spiral HE	The results showed that the heat transfer rate, overall heat transfer coefficient, and performance index increased as either Reynolds number or concentration increased.
Ionescu and Neagu [91]	Al_2_O_3_–water	The effect of using a water-based nanofluid on the performance of a cross-flow micro HE	It was found that the total heat flow rate, pressure drop, and friction factor increased with increase in nanoparticle volume concentration
Hosseini et al. [92]	CNT-water	Heat transfer performance of the nanofluid in a shell-and-tube intercooler in a LPG absorber tower	The results indicated that increasing either the nanofluid or hot fluid mass flow rates led to an increase in the heat transfer rate.
Albadr et al. [93]	Al_2_O_3_–water	Heat transfer performance	The results showed that the convective heat transfer coefficient of the nanofluid was slightly higher than that of the base fluid at the same mass flow rate and inlet temperature.
Zamzamian et al. [94]	Al_2_O_3_–water and CuO-water	Forced convective heat transfer coefficient in plate-fin and double pipe heat exchangers in turbulent flow regime	The results showed a significant increase in convective heat transfer coefficient when using nanofluids compared to the base fluid, and as the nanoparticles concentration increased, so did the convective heat transfer coefficient.
Rao et al. [95]	Al_2_O_3_–water	The heat transfer rate of nanofluids in a shell-and-tube HE	The result showed that the heat transfer rate increased with decreasing particle size in the nanofluid.

**Table 6:** Thermal conductivities of common solid materials suitable for use as nanoparticles [99].

Materials	Thermal Conductivity [W/(m.K)]
Diamond	2300
Carbon Nanotubes	2000
Silver	429
Copper	401
Aluminium	237
Silicon	148
Silicon Carbide (SiC)	120
Alumina (Al_2_O_3_)	40

## Data Availability

The data presented in this study are available on request from the corresponding author.
